# Isomerism and Relaxation Properties of Lanthanide(III) Complexes of a Ditopic Ligand with Two DO3A Units Bridged by a Methylene-bis(phosphinate) Spacer

**DOI:** 10.1021/acs.inorgchem.6c00452

**Published:** 2026-05-12

**Authors:** Adam Svítok, Carlos Platas-Iglesias, Petr Hermann

**Affiliations:** † Department of Inorganic Chemistry, Faculty of Science, Charles University, Hlavova 2030, 12843 Prague 2, Czech Republic; ‡ Centro Interdisciplinar de Química e Bioloxía (CICA) and Departamento de Química, Facultade de Ciencias, 16737 Universidade da Coruña, 15071 A Coruña, Galicia, Spain

## Abstract

A ligand with two DO3A units connected by a methylene-bis­(phosphinate) spacer (ligand **L2**) and a DOTA analogue with one methylene-bis­(phosphinate) pendant arm (ligand **L1**) were prepared, and their mono/dinuclear lanthanide­(III) complexes were studied. Their twisted-square antiprismatic (TSA)/square-prismatic (SA) isomers exhibit only one (*vertical*) configuration of the chiral phosphorus atom, leading to two/six major diastereo­isomers for the mono/dinuclear complexes, respectively. All isomers mutually exchange, but in the **L2** complexes, the TSA/SA exchange occurs only through macrocycle inversion. Phosphinate rotation exchanging the phosphorus atom chirality was not observed. In the Ln_2_-**L2** complexes, each unit behaves independently and has one coordinated water molecule up to the Er­(III) complex (as determined by Eu­(III) fluorescence and ^89^Y NMR). The Ln–Ln distance is about 5.7 Å. Per Gd­(III) ^1^H relaxivities of the mono/dinuclear complexes are almost identical, revealing no significant Gd–Gd interaction in the dinuclear complex. The molecular relaxivity of the Gd_2_-**L2** complex (14.4 mM^–1^ s^–1^, 0.94 T, 25 °C) is among the highest values measured for a monohydrated dinuclear complex and is almost the same at 0.94 and 7 T. Since the methylene group of the bridge can be synthetically modified, this paves the way to targeted magnetic resonance imaging (MRI) contrast agents (CAs) with high relaxivity at high magnetic fields.

## Graphical abstract

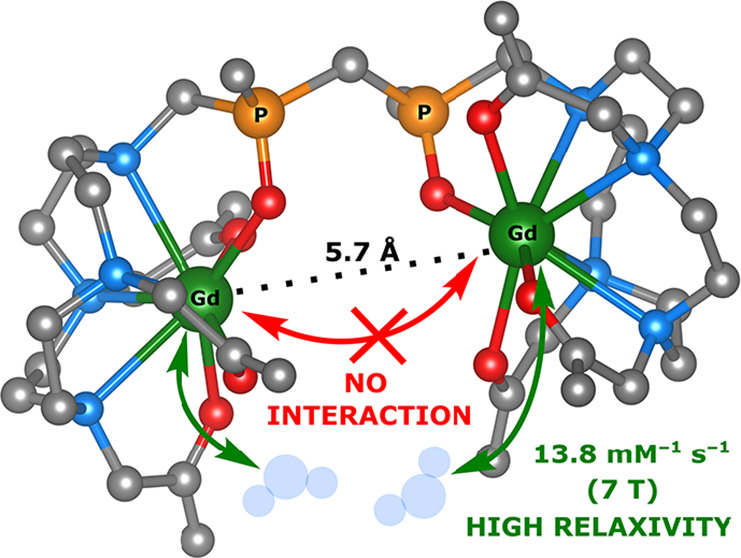

## Introduction

Magnetic resonance imaging (MRI) is a widely used clinical imaging method that provides valuable and precise diagnostic information. To further improve the resolution and diagnostic value of MRI, contrast agents (CAs) are used in approximately 40% of MRI clinical examinations.^1^ Most MRI CAs currently in use are based on Gd^iii^ complexes of DOTA derivatives (Figure 1) due to their high kinetic inertness and thermodynamic stability, which prevent the toxicity of free Gd^iii^ aqua-ion released upon complex dissociation.^1–3^ However, MRI scanners have rapidly evolved since these CAs were designed. Notably, the magnetic fields used in scanners have increased over the years. Initially, when Gd^iii^-DOTA-based CAs were developed, 0.47-T MRI scanners were commonly used. They were gradually replaced by 1.5-T and 3-T MRI scanners, and 7-T MRI scanners are now under development for clinical use.^4^ There have also been increasing concerns over potential long-term risks of using the Gd^iii^-based MRI CAs.^5–7^ Thus, more efficient CAs are highly desired as they would mitigate the risks of toxicity by reducing doses in clinical practice. Moreover, the use of MRI CAs is declining due to technological improvements in MRI scanners. On the other hand, responsive and/or targeted MRI CAs whose MRI signal intensity depends on factors such as metal ion concentration, pH, or receptor affinity are sought as they provide valuable information in the field of personalized medicine, such as early tumor detection or imaging of physiological processes.^8–11^ For such CAs, a high efficiency of water relaxation enhancement is also required.

**Figure 1:**
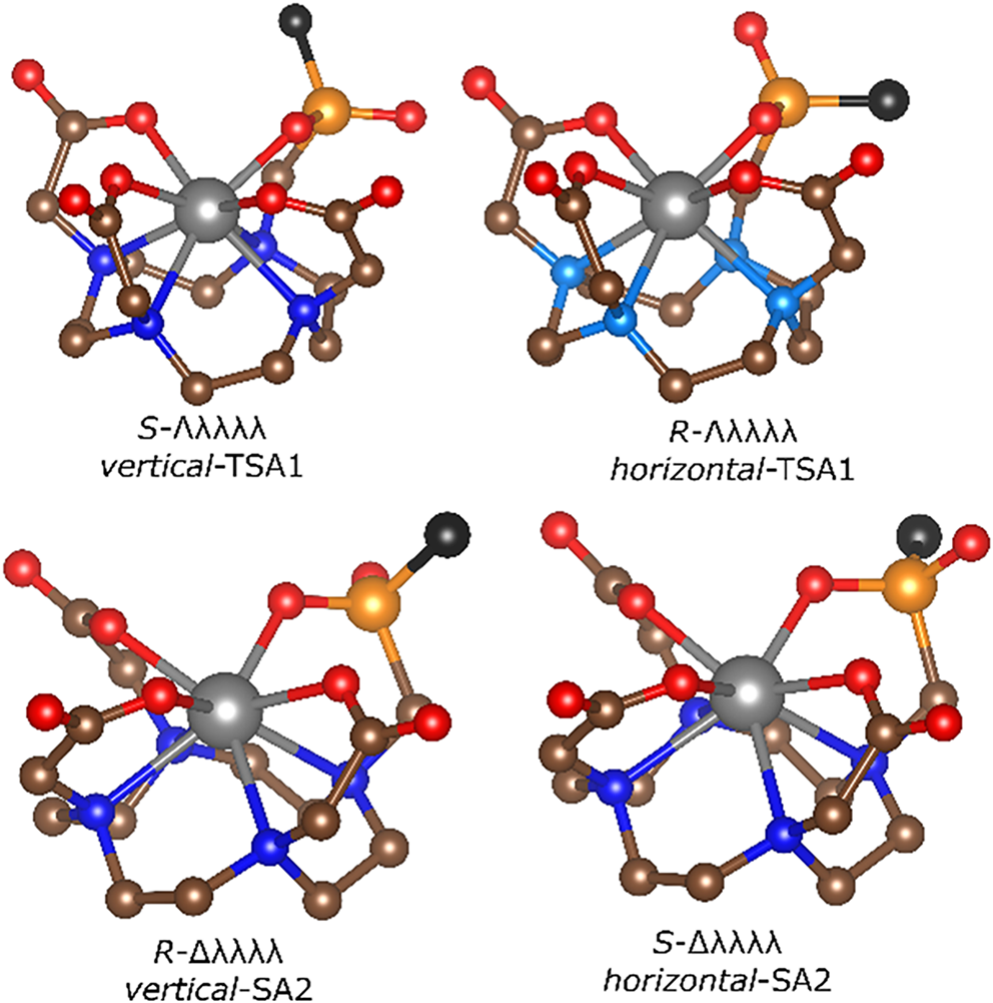
Schematic representation of four diasteroisomers (one enantiomer is always shown) of Ln^iii^ complexes of monophosphinate DOTA analogues showing a relative position of the phosphorus atom substituent (black) and the O_4_ plane (“*vertical*” and “*horizontal*” isomers).

Efficacy of ^1^H MRI CAs is assessed by their relaxivity, defined as an increase in the water *R*_1_ relaxation rate per 1-mm Gd^iii^ concentration. Many factors affect relaxivity, but only a few are important at high magnetic fields, and they are relatively well-predictable when designing new CAs.^1,12^ These parameters are the rotation correlation time, τ_R_, the water residence time, τ_m_, and the number of water molecules coordinated to the Gd^iii^ ion, *q*. Usually, one water molecule is coordinated to the central Gd^iii^ ion, but the number of water molecules in the second sphere, *q*_ss_, also has a significant effect in some cases.^13^ In most complexes used as MRI CAs in the clinic, one water molecule is directly coordinated; thus, *q* = 1. The optimal values of the rotational correlation times depend on the external magnetic field strength. For external magnetic fields up to ∼1.5 T, the relaxivity increases with long τ_R_ values that can be attained by attaching many small Gd^iii^ complexes to a macromolecule.^14,15^ However, for external magnetic fields above 1.5 T, there is an optimal value of the rotational correlation time, and both lower and higher values steeply decrease the relaxivity.^16^ For external magnetic fields in the range of 1.5–9 T, optimal values of τ_R_ are in the range of 0.5–1.5 ns.^17^ These values correspond to oligomeric complexes with a molecular weight of 1–3 kDa.^18,19^ Thus, unlike the simple Gd^iii^-DOTA-based complexes used as ^1^H MRI CAs in current medical practice, multimeric complexes are the “ideal” ones for the higher external magnetic fields now being employed. The higher relaxivities at higher magnetic fields have been confirmed for dinuclear and oligonuclear Gd^iii^ complexes of bis- and oligo-macrocyclic derivatives of DOTA with various linkers.^20–30^ For these multimeric complexes, rigidity of the linker is also a crucial parameter, as flexible linkers lead to rapid local motion of the ligand/complex subunits, shortening the local rotational correlation times.^20,29–31^ The increased rigidity of the CAs is often associated with a shorter Gd^iii^ –Gd^iii^ distance; if the ions are too close, their mutual electron-spin interaction increases, which changes the properties of the whole system, particularly at low external magnetic fields.^32^

The other tunable parameter is the water residence time τ_m_ (or water-exchange rate, *k*_ex_ = 1/τ_m_). This parameter also has different optimal values at different external magnetic fields, and a very fast water exchange increases relaxivity at high magnetic fields. To obtain MRI CAs effective at high magnetic fields, a complex of an oligomeric derivative should have an optimally fast water exchange. The Gd^iii^ complexes of DOTA-monoamides, which are the most used in dimeric/multimeric MRI CAs, exhibit nonoptimal (too long) water residence times,^12,20,29^ and thus, the maximal predicted relaxivity cannot be reached. It is also known that twisted-square antiprismatic (TSA) isomers of the complexes exchange coordinated water molecules faster than square-prismatic (SA) isomers.^33–36^ Thus, a high abundance of the TSA isomer is beneficial and can yield optimal values of τ_M_ for Gd^iii^ complexes. To reach the predicted high relaxivity, optimal water residence time must be coupled with optimal local/global molecular tumbling.^37^ Additionally, higher second-sphere hydration of the complexes is advantageous, as it can contribute up to 15% to the overall relaxivity.^12,38^ Despite not being fully optimally designed, gadoquatrane—a tetranuclear Gd^iii^ complex of pentaerythritol derivative with four DOTA-monoamide units, has recently entered clinical trials as an efficient MRI CA.^39,40^

The parameters named above may be improved by substituting one pendant arm in DOTA with a methylphosphinate/phosphonate group, yielding the DO3AP^R^ ligand family (Chart 1). Due to the bulkiness of the phosphonate/phosphinate moiety, the substitution shortens the water residence times and increases TSA abundance in the Gd^iii^ complexes.^37,38,41–45^ These phosphorus-containing groups are more hydrophilic than acetates, and thus, second-sphere hydration is also enhanced.^38,42,46^ Following these design principles, dinuclear Ln^iii^ complexes of a ditopic ligand CS­(DO3AP^ABn^)_2_ (Chart 1) formed by two DO3A units with 4-amino-benzylphosphinic acid pendants (DO3AP^ABn^, Chart 1), linked through a thiourea unit, were investigated.^24^ It was shown that this dinuclear Gd^iii^ complex has high relaxivity due to an almost optimal water residence time and increased second-sphere hydration. However, the structures of these ligands are difficult to modify synthetically for targeting purposes.

**Chart 1:**
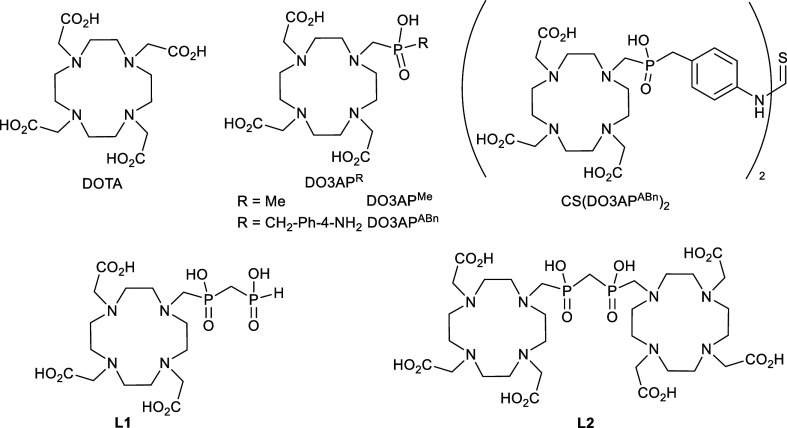
Ligands Discussed in the Text

This study aims to investigate the properties of a ditopic ligand **L2** with a short, modifiable methylene-bis­(phosphinate) linker (Chart 1) to further understand the behavior of oligonuclear Ln^iii^ complexes based on the DO3AP^R^ ligand family. We report here on isomerism, dynamics, and relaxation properties of these complexes. To better understand the properties, complexes of a structurally similar monotopic ligand (**L1**, Chart 1) were also investigated. Throughout the text, charges of the ligands and complexes are omitted unless they are necessary for comprehension.

## Results and Discussion

### Synthesis

The ditopic ligand **L2** was prepared according to Scheme 1. Diisopropyl methylene-bis­(phosphinate), **1**, prepared from CH_2_(PCl_2_)_2_ (see SI), was attached to *t*Bu_3_DO3A by a phospha-Mannich reaction in a toluene/pyridine mixture as a solvent. Pyridine is an effective solvent for this reaction^45^ and was diluted with toluene here to prevent de-esterification of the starting diester. To minimize the extent of side reactions, a relatively low temperature and a long reaction time were required. The fully esterified compound **2** was obtained as a mixture of two diastereo­isomers. After the deprotection of the carboxylates by trifluoroacetic acid, the intermediate was purified on a weak cation-exchange resin. Hydrolysis of the isopropyl phosphinate ester groups afforded ligand **L2** in approximately 15% overall yield (based on *t*Bu_3_DO3A). The low yield may be attributed mainly to competing reductive *N*-methylation and *P*-hydroxymethylation during the phospha-Mannich reaction, as well as losses during purification on the weak cation exchanger.

**Scheme 1:**
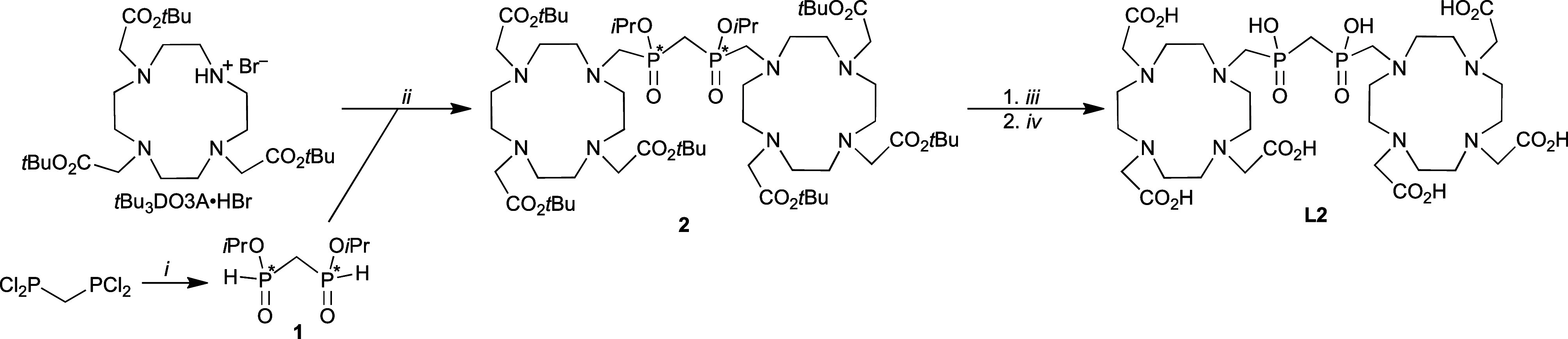
Synthesis of Ligand **L2**: (i) *i*PrOH (4 equiv)/py (2 equiv), Anhydrous THF, 0 °C to Room Temperature, 24 h; (ii) (CH_2_O)_*n*_ (∼5 + 1 equiv), py/Toluene (1:2), 30 °C, 12 d; (iii) TFA/CH_2_Cl_2_ (1:1), Room Temperature, 48 h; Amberlite CG50 Purification; (iv) aq. HCl 1:1, 80 °C, 2 d

Homodinuclear complexes of **L2** with all Ln^iii^ ions (except Pm^iii^) were prepared by a standard procedure using a slight excess of the Ln^iii^ chloride (2.1–2.3 equiv) at pH 5–6 and removing the excess Ln^iii^ as a hydroxide precipitate and/or by chromatography. The Gd^iii^ and Eu^iii^ monocomplexes of **L2** were prepared by reaction of the ligand with 1 equiv of the corresponding chloride and separating the ligand, mononuclear complex, and homodinuclear complex by chromatography on a weak cation-exchange resin. Heterodinuclear complexes were prepared by reacting the corresponding mononuclear complex with a slight excess (1.1–1.2 equiv) of a different LnCl_3_ salt. The monotopic model ligand **L1** was prepared following a known procedure,^47^ and its complexes were prepared by reacting **L1** with 1.1–1.2 equiv of LnCl_3_ and precipitating the excess of the Ln^iii^ ion as a hydroxide. Throughout this text, charges of the complexes, the coordinated water molecules, and charge-balancing cations will be omitted from the formulas, which will be used in the form [*n*Ln­(ligand)] (*n* = 1 and 2 for mono- and dinuclear complexes, respectively) unless a fully correct formula will be necessary for comprehensiveness.

### Solution Isomerism

First, aqueous solutions of the Ln^iii^ complexes were studied by NMR to obtain detailed information about their isomeric composition. Complexes of DOTA derivatives form two diastereo­isomers by combining two possible macrocyclic conformations (δδδδ/λλλλ) and two possible orientations of pendant arms (Λ\Δ). The diastereo­isomers are characterized by different coordination polyhedra: twisted-square antiprism (TSA) existing as two enantiomers, Λλλλλ (TSA1) and Δδδδδ (TSA2), and square antiprism (SA) existing as two enantiomers, Λδδδδ (SA1) and Δλλλλ (SA2). In complexes of monophosphinate derivatives of DOTA (e.g., **L1**), the phosphorus atom becomes chiral after coordination, and thus, the TSA/SA isomerism combines with two possible phosphorus atom configurations *R*/*S* to form four diastereo­isomers, each being a pair of enantiomers: *R*-Δλλλλ/*S*-Λδδδδ = *v*-SA, *S*-Δλλλλ/*R*-Λδδδδ = *h*-SA, *S*-Λλλλλ/*R*-Δδδδδ = *v*‑TSA, and *R*-Λλλλλ/*S*-Δδδδδ = *h*-TSA (Figure 1). The *v* and *h* labels correspond with *vertical* and *horizontal* configurations on the phosphorus atom, respectively, given by the relative position of the phosphinate substituent with respect to the O_4_ plane of the complex (Figures 1 and S1).^48^ In such a way, only one label is used for both spectrally nondistinguishable enantiomers of each diastereo­isomer.

For complexes of phosphinate DOTA analogues with *P*-alkyl substituents, only one, *vertical*, phosphorus configuration is preferred for each SA/TSA isomer.^43,46,50–52^ Here, the phosphorus substituent is an “alkyl” (i.e., the methylene group), and therefore, the diastereo­isomers are likely the *vertical* ones (i.e., *v*-TSA and *v*-SA, Figures 1 and S1). They were previously identified as the preferred diastereo­isomers in the [Ln­(do3ap^Me^)] complexes and are predicted as the more stable ones in complexes of all phosphinate DOTA analogues with a branched −CR_3_ group directly connected to the phosphorus atom.^48^

The *vertical* configuration is also expected in each subunit of the dinuclear [2Ln­(**L2**)] complexes, where only diastereo­isomers with a single phosphorus configuration were detected (see below). The possible isomers for these homodinuclear complexes are summarized in Figure 2 and Table S1. Combining the *v*-SA1, *v*-SA2, *v*-TSA1, and *v*-TSA2 configurations on each subunit, six diastereo­isomers are possible: two diastereo­isomers with both subunits having TSA geometry, two diastereo­isomers with both subunits in SA geometry, and two diastereo­isomers with mixed TSA/SA geometry. The diastereo­isomers with both subunits in the TSA or both subunits in the SA geometry are labeled “*sm*” (the “*same*”) or “*df*” (“*different*”) if the subunits are mutually identical or different enantiomers, respectively. The diastereo­isomers with the mixed TSA–SA arrangements are labeled as “*arm*” or “*cyc*” if they present the same pendant “*arm*” orientation or the same “*macrocyclic*” conformations in each subunit, respectively. All diastereo­isomers, except *df*-TSA and *df*-SA being present as *meso*-forms, exist as two enantiomers (Figure 2 and Table S1). These isomers, along with the complete description of their stereochemistry and the theoretical number of their ^31^P NMR signals, are illustratively shown in Figure 2.

**Figure 2:**
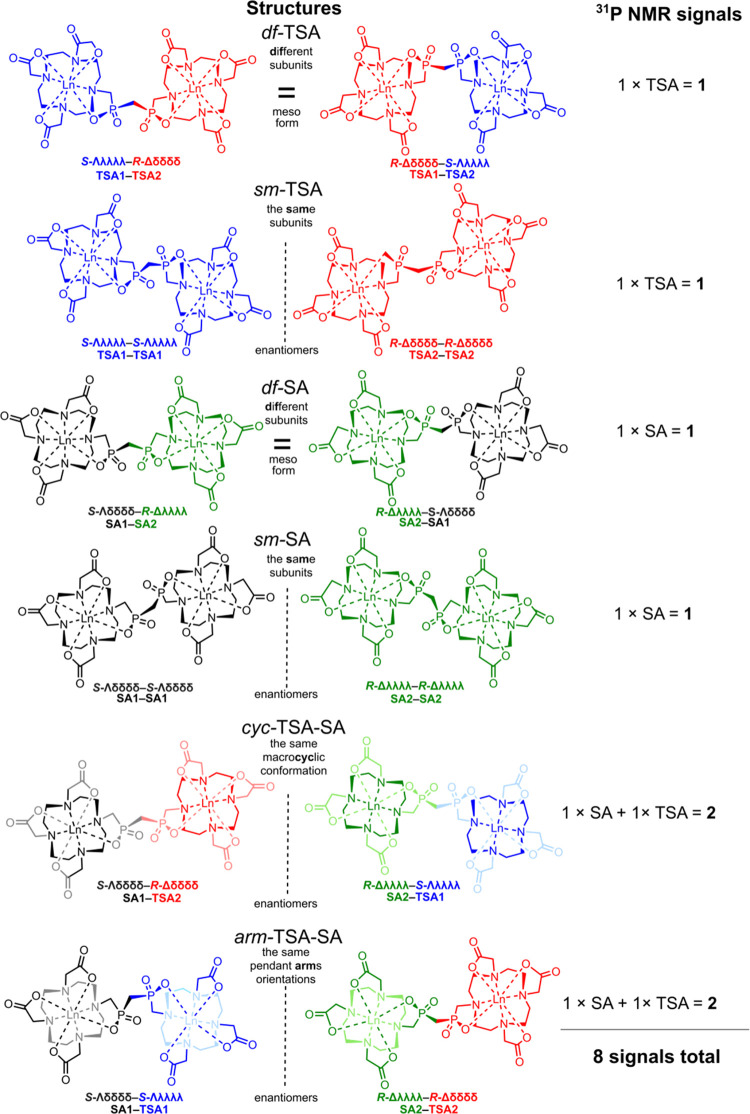
Six possible diastereo­isomers of the [2Ln­(**L2**)] homodicomplexes formed by combining the TSA1, TSA2, SA1, and SA2 subunits if only one possible (“*vertical*”) phosphorus atom configuration is considered.

The presence of only one configuration on the phosphorus atom was confirmed experimentally. Only two major diastereo­isomers could be reliably identified in ^1^H and ^31^P NMR spectra of the [Ln­(**L1**)] complexes (Figures 3, S2, and S3). By comparing the ^1^H NMR signals of “axial” protons (the closest ones to the pseudo-*C*_4_ axis and, thus, with the largest lanthanide-induced shift (LIS), see SI of ref 49 for details) with those of [Ln­(dota)], the two diastereo­isomers were identified as SA and TSA species, and they should exhibit a *vertical* phosphorus configuration. The two minor isomers, *h*-SA and *h*-TSA, could only be detected in complexes of **L1** with some Ln^iii^ ions at a very low (<2%) abundance (Figure 3), but they were mostly undetectable due to overlaps of their signals with other signals or with baseline distortions (Figures S2 and S3).

**Figure 3:**
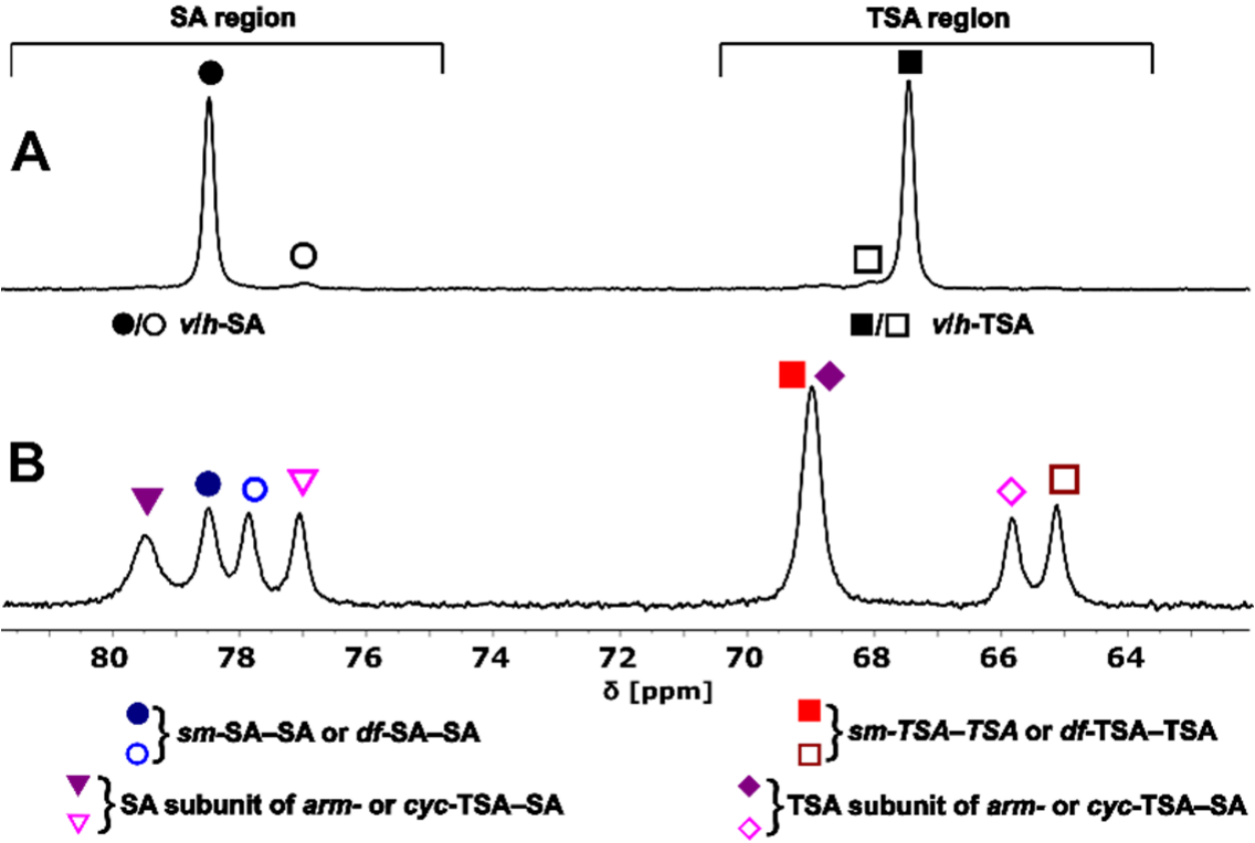
Comparison of parts of the ^31^P NMR spectra containing (A) signals of coordinated phosphinate group of the four diastereo­isomers of the [Eu­(**L1**)] complex (*vertical*/*horizontal*-SA/TSA, two and two signals in SA and TSA region, respectively) and (B) signals of the phosphinate group of all diastereo­isomers of the [2Eu­(**L2**)] complex (four and four signals in TSA and SA regions, respectively).

Similarly, only the two *vertical* diastereo­isomers, i.e., *v*-TSA and *v*-SA, were found in ^1^H and ^31^P NMR spectra of the mononuclear [1Eu­(**L2**)] complex. The presence of all six diastereo­isomers in solution was confirmed by ^1^H and ^31^P NMR spectroscopy in various [2Ln­(**L2**)] complexes, as shown in Figures 3, S4, and S5. The signals could be assigned to the diastereo­isomers (i.e., to the SA-SA, TSA-TSA, or TSA-SA species) by comparing the ^1^H NMR chemical shifts of the “axial” protons of the isomers to the chemical shifts in analogous complexes of DOTA-like ligands^45,48,49,53^ and by comparing relative integral intensities in the ^1^H and ^31^P NMR spectra. However, the *df*/*sm* and *arm*/*cyc* pairs could not be distinguished. The ^31^P NMR spectra of the mixed *arm*-TSA-SA and *cyc*-TSA-SA isomers each contain two peaks in regions of chemical shifts typical for the TSA and SA isomers. The *sm*-TSA, *sm*-SA, *df*-TSA, and *df*-SA contain only one ^31^P NMR signal with chemical shifts in the corresponding regions (Figure 3).

The change in the TSA/SA ratio along the lanthanide series was determined from the ^31^P and/or ^1^H NMR relative integral intensities of the resonances of the [Ln­(**L1**)] and [2Ln­(**L2**)] complexes at 5 °C, where signals are best resolved as the dynamic processes are sufficiently slow (Figure 4). For the homodinuclear [2Ln­(**L2**)] complexes, the sum of signals corresponding to *sm*-TSA, *df*-TSA, and the TSA subunits of *arm*/*cyc*-TSA–SA was used as the TSA abundance (and analogously for the SA isomers). Moreover, the abundance of the TSA is given by a sum of nona-coordinated TSA and octacoordinated anhydrous TSA′ species, which are in equilibrium and are usually indistinguishable by NMR.^54^ The diastereo­isomer ratio dependence is analogous to that of the Ln^iii^ complexes of DOTA and its monophosphorus acid-containing derivatives.^38,42,45,49,55,56^ The ligand cavity size decreases in the order TSA > SA > TSA′.^55,57^ As smaller Ln^iii^ is located deeper in the ligand cavity, the abundance of diastereo­isomers with a smaller ligand cavity increases. Thus, the La^iii^ and Ce^iii^ complexes of the title ligands are present in solution only as the TSA diastereo­isomers, and with decreasing Ln^iii^ size, the abundance of the SA species increases until the Er^iii^ complex. For complexes of the smallest Ln^iii^ ions, the abundance of the SA isomers decreases due to the gradually higher abundance of the anhydrous TSA′ isomer. However, the decrease of TSA abundance with decreasing Ln^iii^ size is less significant than that of the [Ln­(dota)] complexes and is similar to that of complexes of other monophosphorus acid DOTA analogues.^38,42,45,49^ The differences are caused by the bulkiness of the phosphonate/phosphinate group. The water-exchange rate is much faster for the TSA diastereo­isomer,^33,34^ and thus, knowledge of the fraction of the TSA species for the Gd^iii^ complexes of the title ligands is important for a modulation of relaxivity. The TSA fraction in [Gd­(**L1**)] and [Gd­(**L2**)] complexes, ∼42% at 25 °C, was obtained by interpolation of the TSA abundances of the Eu^iii^ and Tb^iii^ complexes. This value increases with temperature and is comparable to values observed for complexes of other monophosphorus acid DOTA derivatives.^38,45,49^ The value is higher than that for the [Gd­(dota)] complex (∼15% of TSA, 25 °C),^55^ which also contributes to the high relaxivity of the title complexes (see below). The “hydration break” (i.e., an onset of presence of the anhydrous TSA′ isomer) is shifted here to the Er^iii^ complexes from the Tb^iii^–Ho^iii^ complexes of methylphosphinate,^48^ monoethylphosphonate,^38,49^ or dibenzylamino-methylphosphinate^45^ derivatives of DOTA, likely due to the larger bulkiness of the methylene-bis­(phosphinate) group decreasing the TSA′ stability.

**Figure 4:**
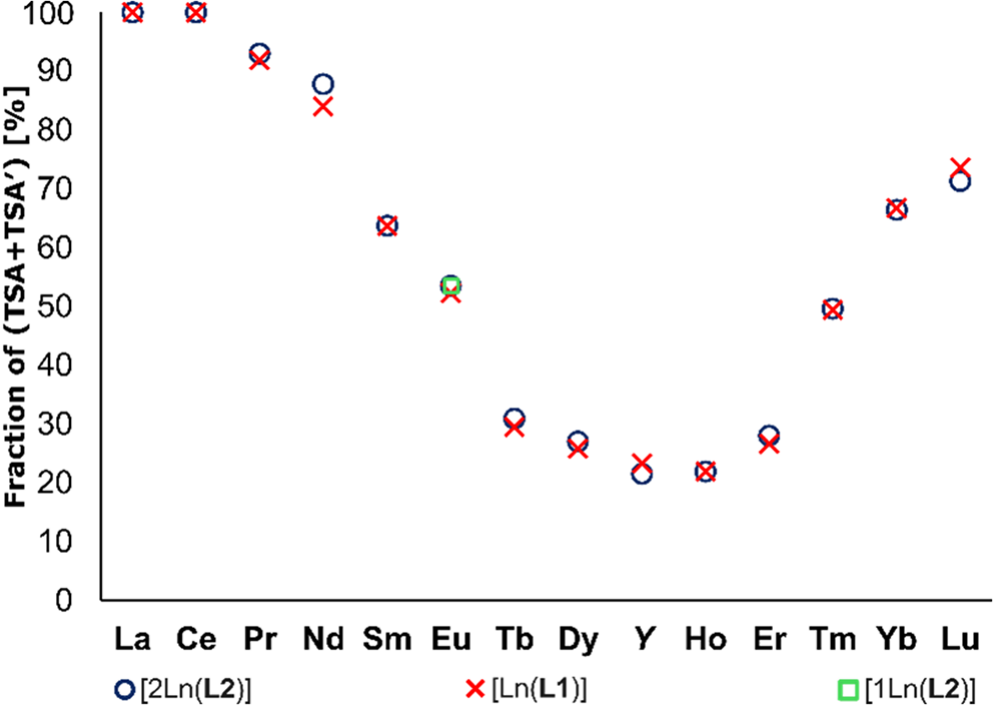
Sum of the molar fractions of TSA + TSA′ diastereo­isomers along the lanthanide series for the mononuclear [Ln­(**L1**)], [1Eu­(**L2**)] complexes, and homodinuclear [2Ln­(**L2**)] complexes and the corresponding Y^iii^ complexes at 5 °C in D_2_O.

The diastereo­isomer abundance is the same for the complexes of the monotopic and ditopic ligands, and thus, it is not affected by the presence of the other subunit in the dinuclear complexes. This confirms that the dinuclear complexes may be treated as two independent DO3AP^alkyl^/**L1** subunits. Combinations of two types of TSA (TSA1/TSA2) and two types of SA (SA1/SA2) diastereo­isomers lead to six diastereo­isomers of [2Ln­(**L2**)] complexes (see above), whose relative abundance differs. Even for each pair of *sm*/*df*-TSA, *sm*/*df*-SA, and *arm*/*cyc*-TSA–SA isomers, one diastereo­isomer is preferred. However, no method could be devised to reliably distinguish the signals of these diastereo­isomeric pairs.

### Solution Structure of the Complexes

Aside from the diastereo­isomer abundance, the number of coordinated water molecules and the mutual orientation of the macrocyclic subunits are also expected to affect the overall relaxivity. Thus, the solution structures of the complexes were further studied. The number of water molecules coordinated to the Gd^iii^ ion, *q*, was estimated from the difference of excited-state lifetimes of the Eu^iii^ ion in the [Eu­(**L1**)] and [2Eu­(**L2**)] complexes measured in H_2_O, τ_H_, and in D_2_O, τ_D_, as H_2_O molecules are more effective luminescence quenchers.^58,59^ For the [2Eu­(**L2**)] complex, the number of coordinated water molecules determined for each Eu^iii^ ion was *q* = 0.9–1 (Figure S6). This value is typical for monohydrated Eu^iii^ complexes of H_4_dota, its phosphinate/phosphonate derivatives (Table S2 and Figure S2 and the corresponding text), and another bis-macrocyclic system bridged by piperazine.^60^ Surprisingly, the excited-state lifetime measured for the mononuclear [Eu­(**L1**)] complex (τ_H_ 1.63 ms, Figure S7) suggests the absence of a coordinated water molecule. To get more information about the coordination environment in these complexes, the fluorescence spectra of [Eu­(**L1**)], [Eu­(do3ap^Me^)], [1Eu­(**L2**)], and [2Eu­(**L2**)] complexes were measured. The shape of the spectra is identical for all complexes (Figure S9), suggesting that they have similar coordination environments.

To confirm the coordination of a water molecule in the complexes by a different method, we used ^89^Y NMR spectroscopy. The ^89^Y NMR shift is predictable by an empirical equation based on the number and type of coordinated groups, with the presence of H_2_O as a ligand changing the ^89^Y NMR shift by approximately 100 ppm.^61^ To simplify the system, we calculated (by DFT) the ^89^Y NMR shift for all four possible diastereo­isomers of the Y^iii^ complex of a mono­(methylphosphinate) derivative, DO3AP^Me^ (Chart 1), whose coordination sphere is expected to be identical to that of the dinuclear complex [2Y­(**L2**)] but without a free coordinating group present, unlike that in **L1**. The ^89^Y chemical shift can also be predicted by an empirical eq 1 ^61^

1 $$\delta \left(Y\right)=A-\left(S_{\mathrm{Nam}}n_{\mathrm{Nam}}+S_{\mathrm{OC}}n_{\mathrm{OC}}+S_{\mathrm{Ow}}n_{\mathrm{Ow}}+S_{\mathrm{OP}}n_{\mathrm{OP}}+...\right)$$

where *A* = 863 ppm is an empirical constant corresponding to a theoretical δ_Y_ without any ligands, *S*_Nam_, *S*_OC_, *S*_OP_, and *S*_Ow_ represent the shielding contributions of coordinated amine, carboxylate, phosphinate groups, or water, respectively, and *n*_X_ represents the number of the corresponding donor atoms. The values of *S*_Nam_, *S*_OC_, and *S*_Ow_ are known,^60^ and the value of *S*_OP_ may be determined as ∼82 ppm based on chemical shifts of Y^iii^ complexes of tetrakis­(methylenephosphinate) DOTA analogues published previously.^52^ The value calculated according to eq 2 agrees reasonably well with the δ_Y_ value measured for the monohydrated [Y­(H_2_O)­(dota)]^−^ complex having CN 9 (112 ppm).^52^

The experimental and calculated shifts for all diastereo­isomers are compared in Table S3. For the [Y­(do3ap^Me^)] complex, the major diastereo­isomer is the *v*-SA isomer, whose experimental ^89^Y chemical shift of 106.9 ppm agrees with that calculated for the structure containing the coordinated water molecule (117.6 and 119 ppm based on DFT and eq 1, respectively). However, the experimental chemical shift of *v*-TSA (157.8 ppm) lies between those of the diastereo­isomers with and without a coordinated water molecule, 102.2 and 192.6 ppm, respectively. This may be explained by an equilibrium between the TSA and TSA′ isomers, which is expected for the Y^iii^ complex, as the effective ionic radius of the octacoordinated Y^iii^ (1.019 Å, CN 8) is between those of Dy^iii^ and Ho^iii^ (1.027 and 1.015 Å for CN 8, respectively).^62^ Thus, the [Y­(do3ap^Me^)] complex is near the “hydration break” for the [Ln­(do3ap^Me^)] complexes.^48^ In the experimental ^89^Y NMR spectra of the dinuclear complex [2Y­(**L2**)], only two signals at 107.2 and 107.4 ppm are present (Figure S10), corresponding to the *v*-TSA and *v*-SA subunits. Unlike that for the [Y­(do3ap^Me^)] complex, both chemical shifts correspond to the species with a coordinated water molecule. This is consistent with the shift of “hydration break” in the complexes of **L2** (Figure 4) compared with that of complexes of DO3AP^Me^.^48^ Similarly, the ^89^Y chemical shifts of the *v*-TSA and *v*-SA diastereo­isomers of the heterodinuclear [GdY­(**L2**)] complex are 114.3 and 115.6, respectively, corresponding to species with a coordinated water molecule. The chemical shifts are slightly shifted compared with the homodinuclear complex due to the presence of Gd^iii^. Thus, coordination of one water molecule is expected in all diastereo­isomers of [Ln­(**L1)**] and [2Ln­(**L2)**] complexes for Ln^iii^ ions larger than Dy^iii^/Ho^iii^. For complexes of Er^iii^ and smaller Ln^iii^ ions, the anhydrous TSA′ isomers are present in solution, as it is also seen from the abundance of (TSA + TSA′) and SA isomers depicted in Figure 4. These data show that the Gd^iii^ complexes of the title ligand contain one coordinated water molecule.

Moreover, complexes of ditopic ligand **L2** contain the P–C–P bridge around which free rotation is allowed. Thus, information about the diastereo­isomers present in solution is insufficient to assess the solution structure of the complexes, as many mutual orientations of the macrocyclic subunits in each diastereo­isomer are possible. Thus, the dominant mutual orientation of the subunits in solution was determined using paramagnetic relaxation enhancement (PRE) of the ^89^Y NMR signal in the [GdY­(**L2**)] heterodicomplex. In the ^89^Y NMR spectrum of [GdY­(**L2**)], two broad overlapping signals of *v*-TSA and *v*-SA subunits were detected (114.3 and 115.6 ppm, see above). These two signals could not be fully separated, and thus, only an average value of ^89^Y *T*_1_ relaxation time was determined. The *T*_1_ relaxation time of 79 ms is close to the relaxation time of a similar dinuclear system [GdY­{CS­(DO3AP^ABn^)_2_}] (for the ligand structure, see Chart 1), where this method was used previously.^24^ Assuming that contact contribution to the PRE is negligible due to the large number of chemical bonds between the ions, the Y–Gd distance was determined from the paramagnetically enhanced relaxation time of ^89^Y, *R*_1,P_, by a simplified Solomon-Bloembergen expression (eq 2), considering only the dipolar contribution of the nearby Gd^iii^ to the ^89^Y relaxation rate, *R*_1,P_.^63^

2 $$R_{1,P}=\frac{2}{5}{\left(\frac{\mu_{0}}{4\pi }\right)}^{2}\frac{\mu_{\mathrm{eff}}^{2}\beta^{2}\gamma_{Y}^{2}}{r_{Y-\mathrm{Gd}}^{6}}\tau_{R}$$

Here, (μ_0_/4π) = 10^–7^ H m^–1^ is the vacuum permeability, μ_eff_ = 7.94 μ_B_ is the Gd^iii^ effective magnetic moment, β = 9.274·10^–24^ J T^–1^ is the Bohr magneton, and γ_Y_ = 1.3155·10^7^ rad T^–1^ s^–1^ is the ^89^Y magnetogyric ratio. The value of the rotational correlation time of the complex, τ_R_, was estimated to have an average value of 117 ps from ^13^C *T*_1_ relaxation of the cycle and arm −CH_2_– groups of the diamagnetic [2Y­(**L2**)] complex (see Table S4 and the corresponding text in SI). The short value of the rotational correlation time is close to the values determined previously for monomeric complexes^48,64,65^ and corresponds to the movement of the C–H vector. This value corresponds to a local rotational correlation time, indicating that the subunits of the dinuclear complex move independently of each other at a rate similar to that of simple monomeric systems. A rotational correlation time corresponding to the Y–Gd vector would be more suitable for eq 1, but it is difficult to determine experimentally. As the influence of the rotational correlation time on the overall PRE is much smaller than that of the distance (*r* ^*–*6^ dependence), the local rotational correlation time determined from ^13^C NMR was used as a suitable approximation. In a similar approach, a rotational correlation time corresponding to the movement of the water O–H vector was successfully used for this purpose previously.^24^ For [GdY­(**L2**)], the experimental Gd–Y distance determined from eq 2 was 5.7 Å.

The determined Gd–Y distance was compared with the results of DFT geometry optimizations. For this purpose, four diastereo­isomers were investigated by combining the *v*-TSA and *v*-SA geometries on the Gd^iii^ and Y^iii^ subunits while keeping only one (δδδδ) macrocycle configuration for simplicity. Based on the ^89^Y NMR shifts and fluorescence lifetimes (see above), all geometries were optimized with one coordinated water molecule. For each diastereo­isomer, the potential energy surface was analyzed as the dihedral angle defined by the P–C–P–O atoms was varied. The structures in local energy minima were then optimized, and their Gd–Y distances were compared with the experimental value. The experimental (5.7 Å) and DFT-calculated (5.9 Å) Gd–Y distances are in close agreement for the compact structure shown in Figure 5. The structure corresponds to the most abundant *v*-SA­(Y)/*v*-SA­(Gd) diastereo­isomer, as expected from the changes in isomer abundances along the lanthanide series (see above). Here, both subunits are close to each other, making this structure similar to the solution structure of the complexes of the ditopic ligand CS­(DO3AP^ABn^)_2_.^24^ These structures keep coordinated water molecules close to both Gd^iii^ ions in each [2Gd­(**L2**)] and [2Gd­{CS­(DO3AP^ABn^)_2_}] complex, which may contribute to their high relaxivity (see below). The structures of the remaining diastereo­isomers were also predicted by DFT calculations (Figure S11), confirming that all diastereo­isomers preferably form compact structures. The Gd–Y distances increase with the number of TSA subunits, which is consistent with SA geometries being more compact. The free energies calculated by DFT for different minima on the potential energy surface scanned by varying the P–C–P–O dihedral angle increase with increasing Y–Gd distance (Table S5). Thus, compact structures with short Gd–Y distances are energetically more favored. In these structures, the hydrophilic O_4_ planes of the subunits face each other, and the structure is stabilized by a network of hydrogen bonds (Figure 5) with the O–H···O distances in the range of 2.69–2.96 Å.

**Figure 5:**
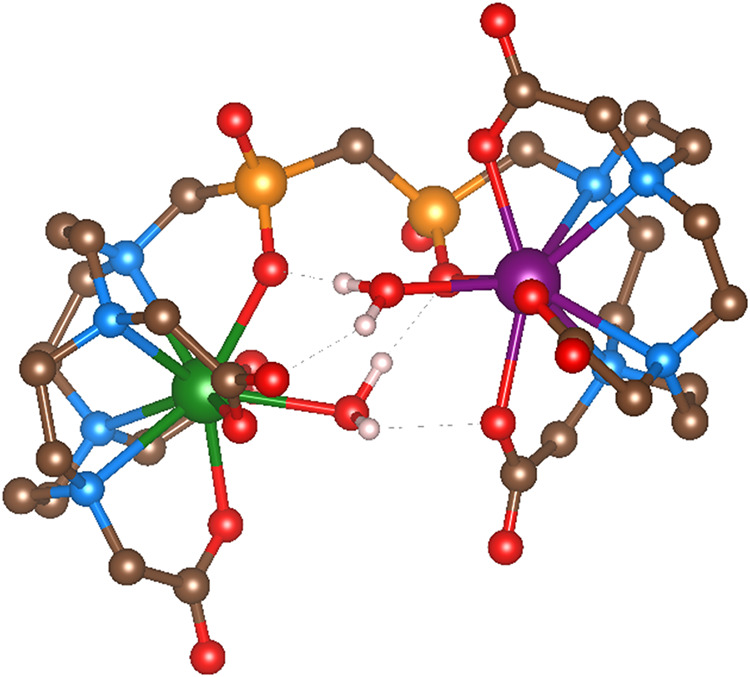
One of the optimized geometries for the [GdY­(**L2**)] complex, consisting of two *v*-SA subunits, that best matches the experimental data. For simplicity, C–H hydrogen atoms are not shown. Color code: hydrogen, white; carbon, brown; nitrogen, blue; oxygen, red; phosphorus, orange; yttrium, green; and gadolinium, purple. Hydrogen bonds between the coordinated water molecules and the opposite subunits are shown as gray dashed lines. The figure was prepared and visualized in Vesta.^66^

### Solution Dynamics

The TSA and SA diastereo­isomers of complexes with DOTA-like ligands mutually interchange in solution by pendant arms reorientation, i.e., through interchange of the Λ and Δ conformations, and/or by macrocycle inversion, i.e., through the interchange of the λλλλ and δδδδ configurations. Additionally, in complexes of monophosphinate DOTA analogues, *horizontal*/*vertical* diastereo­isomers may interchange by phosphinate rotation, a process that reverses the phosphorus *R*/*S* configuration.^48^ These interconversion mechanisms were studied by 2D ^31^P–^31^P and ^1^H–^1^H EXSY in the [Eu­(**L1**)] and [2Eu­(**L2**)] complexes; the *T*_1_ relaxation times of the Eu^iii^ complexes were long enough to allow EXSY measurements, despite the paramagnetic effects.

First, the mononuclear complex [Eu­(**L1**)] was studied. Its ^1^H–^1^H 2D EXSY (Figure S12 and the corresponding text) is analogous to that of the [Eu­(do3ap^Me^)] complex studied previously.^48^ All exchange processes occur, and they were identified based on the diastereo­isomers present, their mutual interchange, and whether they interchange “axial” and “equatorial” hydrogen atoms, as this process occurs only by macrocycle inversion. The *v*-TSA/*v*-SA and *h*-TSA/*h*-SA pairs differ only in macrocycle conformations, so these pairs interchange by macrocycle inversion. Similarly, the *v*-TSA/*h*-SA and *h*-TSA/*v*-SA pairs differ only in pendant arms orientation and mutually interchange by pendant arms reorientation. Analogously to other monophosphorus acid derivatives of DOTA,^48,49^ phosphinate rotation interchanges only the *h*-TSA/*v*-TSA pair, differing only in the phosphorus atom configuration (the *h*-SA/*v*-SA pair is not mutually exchanged). The macrocycle inversion between the *v*-TSA/*v*-SA forms the most intense EXSY cross-peaks as it exchanges two of the most abundant diastereo­isomers.

Next, complexes of ditopic ligand **L2** were studied. In the 2D ^1^H–^1^H EXSY of the [2Eu­(**L2**)] complex, the only detectable process is the macrocycle inversion. This process corresponds to two cross-peaks for each macrocycle signal (Figure 6). One cross-peak is found between “axial” and “equatorial” ^1^H signals and, thus, corresponds to the macrocycle inversion on the subunit containing the atoms (an active exchange). The other cross-peak corresponds to the macrocycle inversion on the other subunit, which does not contain the atom (a passive exchange; for further explanation, see Figure S13 and the corresponding text). This was also observed in 2D ^31^P–^31^P EXSY (Figure S14), where each phosphorus atom exchanges with only two other phosphorus atoms: phosphorus atoms of the *sm*-TSA, *df*-TSA, *sm*-SA, and *df*-SA isomers exchange only with both phosphorus atoms of one of the mixed *cyc*/*arm*-SA–TSA diastereo­isomers. Phosphorus atoms of the mixed *cyc*/*arm*-SA–TSA diastereo­isomers exchange only with phosphorus atoms of one of the *sm*-TSA/*df*-TSA isomers and one of the *sm*-SA/*df*-SA isomers. The pendant arms reorientation and phosphinate rotation were not detected due to the absence of isomers with minor *(horizontal*) phosphorus configurations, which would be involved in these processes. Thus, two groups of three diastereo­isomers may be defined: (i) *df*-SA, *df*-TSA, *cyc*-TSA–SA and (ii) *sm*-SA, *sm*-TSA, *arm*-TSA–SA. Individual diastereo­isomers that are members of only group (i) or only group (ii) differ only in macrocyclic configurations of one or both subunits. Thus, they interconvert rapidly via macrocycle inversion. However, diastereo­isomers which are members of separate groups (i) and (ii) also differ in their pendant arms orientation and/or phosphorus configuration. Thus, the exchange between diastereo­isomers from separate groups requires a combination of macrocycle inversion with at least one other process. These combined processes were not detected in 2D EXSY spectra of the [2Eu­(**L2**)] complex, which were measured at short mixing times due to the fast *T*_1_ relaxation (Figures 6 and S14). However, variable-temperature (VT) NMR experiments confirm that all diastereo­isomers interchange and eventually coalesce into a single signal (Figure S15). These findings are further supported by 2D ^1^H–^1^H EXSY of the [2Ce­(**L2**)] complex (Figure S16), where cross-peaks corresponding to the interchange of the “axial” and “equatorial” protons of *sm*-TSA and *df*-TSA, the only diastereo­isomers present, were identified. This interchange can only occur by combining all processes: macrocycle inversion, pendant arm reorientation, and phosphinate rotation. This combined process could be detected for the Ce^iii^ complex, unlike for the Eu^iii^ complex, likely due to all constituent processes being faster for large Ln^iii^ ions, as has been observed in Eu^iii^ complexes of DOTA derivatives previously.^49,67^

**Figure 6:**
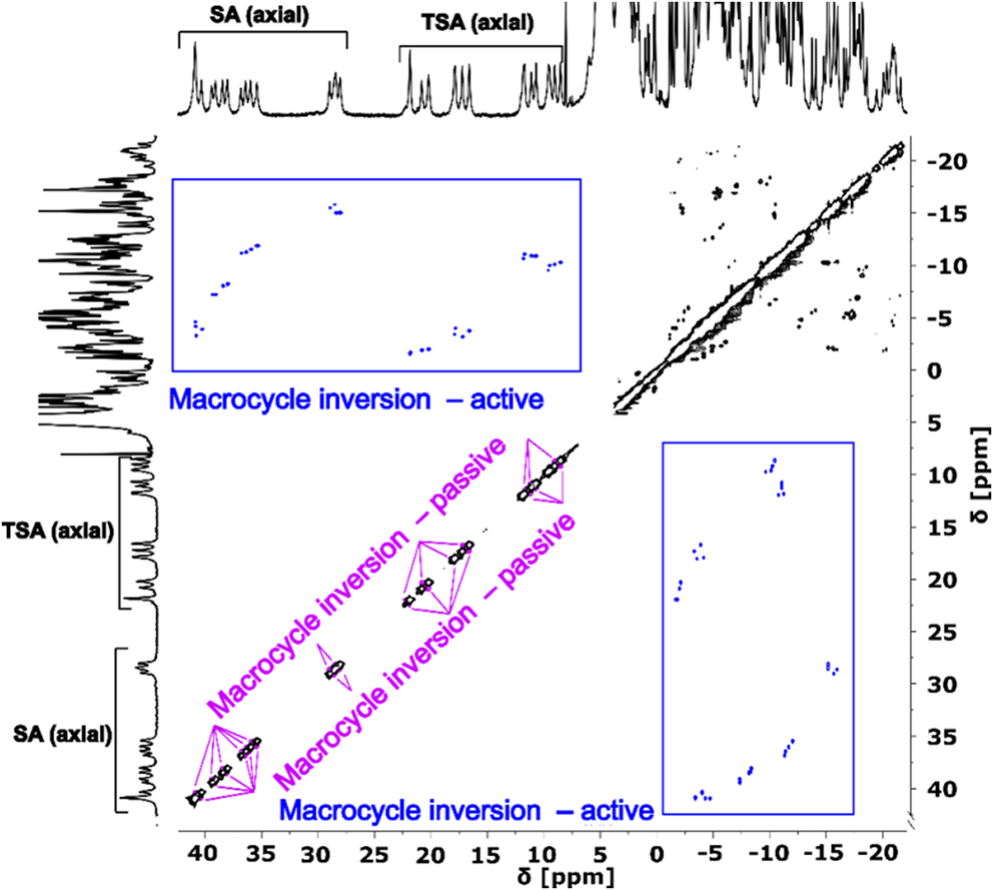
2D ^1^H–^1^H (600 MHz) EXSY spectrum of [2Eu­(**L2**)] at 5 °C, pD ∼ 7, and τ_m_ = 8 ms. Regions containing “axial” protons are labeled in 1D ^1^H NMR traces, and the cross-peaks are assigned to the dynamic processes.

### Relaxivities of Gd^iii^ Complexes

Relaxivities of the [Gd­(**L1**)], [1Gd­(**L2**)], and [2Gd­(**L2**)] complexes were determined at *B*_0_ = 0.94 T (42.5 MHz) and 7 T (300 MHz), *T* = 298 K, and pH 7 (Figure 7). The relaxivities of all complexes are higher than that of [Gd­(dota)] (*r*_1_ = 4.3 mm ^–1^ s^–1^; 298 K, 0.94 T)^20^ being close to that of complexes of other monophosphorus acid DOTA derivatives.^24,38,41–43^ This is a result of the phosphinate group bulkiness, which increases the TSA isomer abundance, leading to a faster water-exchange rate, and high hydrophilicity, which leads to a rich second-sphere hydration. For both [1Gd­(**L2**)] and [2Gd­(**L2**)] complexes, their relaxivity is approximately constant at pH 5–9 (Figure S17). The relaxivity of [Gd­(**L1**)] is higher at pH 5 than at pH 7–9. Reasons for this finding are unknown, but we can speculate that it is related to changes in the arrangement of the second hydration sphere of the complex with pH. The increase in relaxivity of all complexes at pH 3 may be attributed to a prototropic exchange due to a (partial) protonation of the complexes.

**Figure 7:**
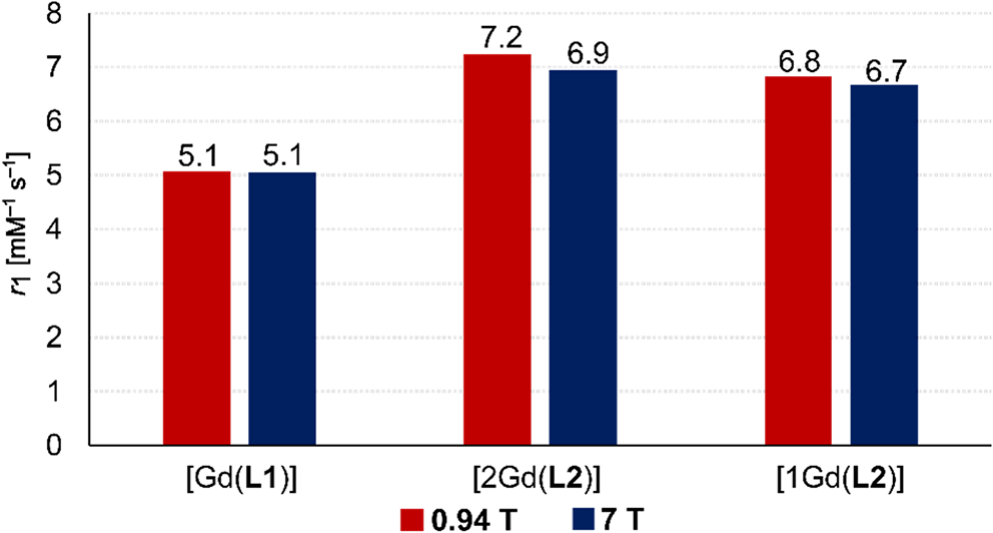
Relaxivities (in mm ^–1^ s^–1^; per Gd^iii^ ion) of the [Gd­(**L1**)], [1Gd­(**L2**)], and [2Gd­(**L2**)] complexes at 0.94 and 7 T (*T* = 298 K, pH 7).

The relaxivities (per Gd^iii^ ion) of both mononuclear [1Gd­(**L2**)] and homodinuclear [2Gd­(**L2**)] complexes are higher than that of the complex of the monotopic ligand, [Gd­(**L1**)], even though macrocyclic units in both ligands contain just one phosphinate pendant arm. This is expected due to the larger molecular size of the [1Gd­(**L2**)] and [2Gd­(**L2**)] complexes. The per-Gd^iii^ relaxivities of the mononuclear [1Gd­(**L2**)] and the homodinuclear [2Gd­(**L2**)] complexes are almost the same. Thus, the high relaxivity of the [2Gd­(**L2**)] complex is not affected by a Gd^iii^–Gd^iii^ interaction of the subunits, at least at the relatively high magnetic field strengths investigated here. The high relaxivity of mono- and dinuclear complexes of **L2** may be attributed to the expected extensive second-sphere hydration, phosphinate bulkiness (resulting in a fast water exchange), and a medium rotational correlation time. Importantly, relaxivities of the **L2** complexes at 0.94 and 7 T are similarly high (Figure 7), confirming the applicability of the title complexes at higher magnetic fields. This is likely the result of an “optimal” rotational correlation time, which, for high magnetic fields, is attained with midsize molecules.^68^ Relaxivities of the [1Gd­(**L2**)] and [2Gd­(**L2**)] complexes are among the highest values known for systems with *q* = 1, very likely due to the expected rich second-sphere contribution to relaxivity. The relaxivity of the [2Gd­(**L2**)] complex (molecular *r*_1_ = 14.4/13.8 mm ^–1^ s^–1^ at 0.94/7.0 T; 25 °C) is comparable to that of the clinically used gadopiclenol (Elucirem), a mononuclear MRI CA with two coordinated water molecules (*q* = 2), exhibiting *r*_1_ values of 12.2/∼11 mm ^–1^ s^–1^ at 1.41/7.0 T at 37 °C.^69^ The dinuclear complex relaxivity is only slightly lower than that of a bis­(benzyl)-thiourea-bridged dinuclear complex, [2Gd­{CS­(DO3AP^ABn^)_2_}], studied previously.^24^ However, the ligand **L2** contains the methylene-bis­(phosphinate) bridge with an easily modifiable −CH_2_– group between the phosphorus atoms; methylene-bis­(phosphinates) with the −CH­(R)– moiety containing various carboxy- or amino-alkyl groups are known.^69–72^ These substitutions allow for the introduction of other reactive groups, affording bifunctional ligands suitable for further conjugation to another molecule to create targeted or multimodal contrast agents with a high relaxivity.^73,74^

## Conclusions

The new ditopic ligand **L2** and its lanthanide­(III) mononuclear and homo- and heterodinuclear complexes were prepared and studied by multinuclear NMR spectroscopy and DFT calculations. The results were compared with analogous data on complexes of monotopic ligand **L1**. The solution studies showed that the homodinuclear complexes form up to six diastereo­isomers. All of them exhibit only one preferential arrangement on the chiral phosphorus atom, and thus, only various *vertical*-TSA and *vertical*-SA isomers were observed. These diastereo­isomers mutually exchange only by macrocycle inversion. Abundances of the TSA/SA isomers of complexes of **L1** and **L2** are the same for complexes of a particular Ln^iii^ ion. The solution dynamics and abundances of the isomers suggest that the complex units in the dinuclear complexes of **L2** act as two independent mononuclear moieties. Based on paramagnetic enhancement of ^89^Y relaxation times by a closely located Gd^iii^ ion in the heterodinuclear complex [GdY­(**L2**)] and on DFT calculations, it was determined that the dinuclear complexes adopt compact structures with the subunits facing each other by approaching their hydrophilic O_4_ planes. The ^89^Y NMR shift and fluorescence lifetime data of the Eu^iii^ complexes confirmed the presence of one Ln^iii^-coordinated water molecule for all complexes in the SA and for complexes of large Ln^iii^ in TSA arrangements.

The relaxivities (per Gd^iii^ ion) of the [1Gd­(**L2**)] and [2Gd­(**L2**)] complexes are virtually identical, confirming that relaxivity is not affected by a Gd^iii^–Gd^iii^ interaction. The relaxivity of the [2Gd­(**L2**)] complex is one of the largest reported to date for a dinuclear monoaquated system and remains very similar across magnetic fields ranging from 0.94 to 7 T, the highest field currently used in MRI. This high value likely results from the medium molecular size of the complex, the fast water-exchange rate (due to the high abundance of the TSA isomer), and extensive second-sphere hydration (due to the solution structure of the complex). The study confirmed that a combination of phosphinate pendant arms and medium molecular size yielded MRI CAs with high relaxivity, even at high magnetic fields.

As the methylene group connecting the phosphorus atoms of the ditopic ligand can be synthetically modified with an orthogonally reactive group, bifunctional ligands with this structural motif can be relatively easily accessible. As such modifications are far from the metal-binding site, the systems should retain the properties of the parent dinuclear complexes. This opens a way for high-relaxivity-targeted MRI CAs based on these systems.

## Experimental Section

### General Methods and Materials

All of the reagents and solvents were obtained from commercial sources and used without purification. Precursor *t*Bu_3_DO3A·HBr^75^ and ligand **L1**·3H_2_O^47^ were synthesized following published procedures, with minor modifications for **L1** (see SI). The phosphinate ester **1** was prepared by alcoholysis of Cl_2_PCH_2_PCl_2_ (Georganics, Slovakia), followed by partial hydrolysis of the prepared phosphite intermediate (see SI). The [Y­(do3ap^Me^)] complex was available from the previous study.^48^ The NMR spectra for characterization of the ligands and intermediates were measured by standard pulse sequences on Varian VNMR 300 (300 and 121 MHz for ^1^H and ^31^P, respectively) or Bruker Avance III 400 (400, 101, and 162 MHz for ^1^H, ^13^C, and ^31^P, respectively) spectrometers. The ^1^H and ^13^C NMR chemical shifts were referenced to the signals of the methyl group of *t*BuOH or TMS added to the samples in D_2_O or CDCl_3_, respectively. The ^31^P NMR chemical shifts were referenced to the chemical shift of 85% aq. H_3_PO_4_ in a coaxial insert tube. The NMR spectra for characterization of Ln^iii^ and Y^iii^ complexes and for special studies (EXSY, variable-temperature NMR, determination of rotational correlation time) were measured on a Bruker Avance III 600 spectrometer equipped with a broadband probe (600, 150, and 243 MHz for ^1^H, ^13^C, and ^31^P, respectively). Spectra of complexes were measured at 5 °C and spectra of ligands and intermediates at 25 °C, unless stated otherwise. For paramagnetic complexes, short values of acquisition times and relaxation delays were chosen to account for the paramagnetic relaxation (Table S6). The number of accumulated transients was chosen to get a satisfactory signal-to-noise ratio. The temperature of the NMR samples was calibrated through the difference of ^1^H chemical shifts of signals of a pure MeOH-*d*_4_ (0–40 °C)^76,77^ or 80% w/w ethylene glycol in dmso-*d*_6_.^78^

Mass spectra were measured in positive and negative modes on an ACQUITY QDA spectrometer (Waters) equipped with a quadrupole detector and an electrospray ionization source. The data were processed with Empower 3 software. Elemental analyses were carried out at IOCB (Prague, Czech Republic) by a combustion analysis for C–H–N content determination on a 2400 Series II Elemental Analyzer (PerkinElmer) and by an X-ray fluorescence analysis for P content on an XEPOS P spectrometer (Spectro). Thin-layer chromatography (TLC) was carried out on Silica Gel 60 F_254_ plates (Merck), and compounds were visualized by I_2_ vapor deposition applied for 10 min or by a 5% aq. KMnO_4_/K_2_CO_3_/NaOH 12:80:1 mixture (compounds with a P–H bond).

### Syntheses

#### Compound **2**

The *t*Bu_3_do3a·HBr (3.35 g, 5.62 mmol, 2.0 equiv) and diisopropyl methylene-bis­(phosphinate) (0.63 g, 2.7 mmol, 1.0 equiv) were dissolved in a pyridine/toluene mixture (1:2, 300 mL). Paraformaldehyde (0.42 g, 14 mmol, 5.1 equiv) was added, and the reaction mixture was stirred at 30 °C for 8 days in a flask closed by a stopper. Additional paraformaldehyde (0.09 g, 3.0 mmol, 1.1 equiv) was added, and the reaction continued for an additional 4 d. Volatiles were evaporated *in vacuo*. The residue was dissolved in CH_2_Cl_2_ (200 mL) and washed with 20% aq. K_2_CO_3_ (2 × 100 mL) and then with H_2_O (1 × 100 mL). The organic phase was dried with anhydrous Na_2_SO_4_. The drying agent was filtered off on a glass frit and washed with CH_2_Cl_2_ (50 mL). Volatiles from the filtrate were evaporated *in vacuo*. The residue was dissolved in a minimal amount of EtOAc and purified by column chromatography (neutral Al_2_O_3_, 300 g). Impurities were eluted with EtOAc, and the product was eluted with *i*PrOH; *R*_f_ ∼ 1. Volatiles from pure product-containing fractions were removed *in vacuo*, and the oily residue containing both diastereo­isomers of compound **2** was used in subsequent reactions without further purification. MS (ESI^+^): *m*/*z* 641.71 [M + 2H]^2+^. ^31^P­{^1^H} NMR (121 MHz, CDCl_3_) δ 44.2 and 44.5 (ratio ∼ 1:1.2).

#### Ligand **L2**

Compound **2** from the previous step was dissolved in a 1:1 TFA/CH_2_Cl_2_ mixture (∼100 mL), and the mixture was stirred at room temperature for 48 h. Volatiles were evaporated *in vacuo*, the residue was dissolved in a minimal amount of acetone, and the crude product was precipitated by adding Et_2_O (∼30 mL). The precipitate was filtered off and washed with Et_2_O (2 × 10 mL). The precipitated crude product was dissolved in water and purified on an Amberlite CG50 weak cation-exchange resin (H^+^-cycle, 8 cm × 5 cm resin bed, impurities were eluted by water, approximately 1000 mL, and the product was eluted by EtOH/conc. aq. HCl 1:1, approximately 300 mL). The volatiles were evaporated from the combined product-containing fractions. The residue was dissolved in water/conc. aq. HCl 1:1 (200 mL), and the solution was stirred at 80 °C for 2 days. The volatiles were evaporated *in vacuo*, and the residue was dissolved in water (50 mL). A small amount of activated charcoal was added to the solution, and the mixture was stirred at 80 °C for 30 min. The charcoal was filtered off using a 0.2 μm syringe filter, and the filtrate was evaporated to dryness *in vacuo*. The residue was dissolved in 0.1% aq. HCl (300 mL), and the solution was lyophilized to obtain the product as a waxy solid (**L2**·4HCl·8H_2_O, 0.5 g, 15% based on *t*Bu_3_DO3A·HBr). ^1^H NMR (600 MHz, D_2_O, 90 °C, pH 1.6) δ 2.50 (t, ^2^ *J*_PH_ 16.6 Hz, 2H, PCH_2_P), 3.27, 3.30, and 3.42 (bs, 3 × 8 H, NCH_2_CH_2_N), 3.51 (d, ^2^ *J*_HP_ 8.0 Hz, 4H, NCH_2_P), 3.55 (bs, 8 H, NCH_2_CH_2_N), 3.83 (s, 8H, NCH_2_CO_2_H), 3.96 and 3.83 (s, 4H, NCH_2_CO_2_H). ^13^C­{^1^H} NMR (151 MHz, D_2_O, 90 °C, pH 1.6) δ 37.5 (t,^1^ *J*_CP_ 81.3 Hz, PCH_2_P), 52.5, 52.8, 54.1, and 55.1 (s, NCH_2_CH_2_N), 57.1 (d, ^1^ *J*_CP_ 95.8 Hz, NCH_2_P), 57.4 and 57.9 (NCH_2_CO_2_H), 173.2 and 175.3 (s, CO_2_H). ^31^P­{^1^H} NMR (243 MHz, D_2_O, 90 °C, pH 1.6) δ 27.0 (s). MS (ESI^+^): *m*/*z* 431.46 [M + 2H]^2+^, 861.33 [M + H]^+^. Elem. analysis: found (required for **L2**·4HCl·8H_2_O): C 32.69 (32.36), Cl 11.09 (12.3), H 6.73 (6.83), N 9.60 (9.74), P 4.5 (5.3).

#### Complexes of **L1**

The ligand **L1**·3H_2_O (∼45 mg, 1 equiv) and LnCl_3_·*x*H_2_O (∼50 mg, 1.1–1.2 equiv) were dissolved in water (∼2 mL). The pH was adjusted to 6.5 and 7.5 with 1% aq. LiOH, and the solution was stirred at 60 °C for 72 h. The solution pH was increased to ∼11 by 1% aq. LiOH, and the mixture was stirred for 10 min to precipitate the Ln^iii^ hydroxide. The precipitate was filtered off with a syringe microfilter (PVDF, 0.2 μm). The filtrate pH was adjusted to 6.5–7.5 with 0.5% aq. HCl. Volatiles were evaporated *in vacuo*, and the residual water was coevaporated with MeOH. The oily residues were dissolved in MeOH (∼10 mL), and the complexes were precipitated by slow addition of acetone or Et_2_O (∼30 mL). The precipitates were collected on a fine glass frit, washed with Et_2_O (3 × 5 mL), and dried in air overnight, yielding the complexes as powders in 65–85% yields. For characterization of complexes, see SI (NMR data in Tables S7 and S8 and Figures S2 and S3; MS data in Table S11).

#### Homodinuclear Complexes of **L2**

The [2Ln­(**L2**)] and [2Y­(**L2**)] complexes were prepared by mixing the ligand **L2**·4HCl·8H_2_O (∼40 mg, 1 equiv) with the corresponding LnCl_3_·*n*H_2_O (∼40 mg, 2.2 equiv) in water and adjusting the solution pH value to 6.5–7.5 by 1% aq. LiOH. The mixture was then stirred at 60 °C for 3 days. The volatiles were evaporated *in vacuo*, and the residue was purified by column chromatography (SiO_2_, 10 g, *i*PrOH/conc. aq. NH_3_/H_2_O 4:1:2, *R*_f_ ∼ 0.4). Volatiles were removed from the pure product-containing fractions *in vacuo*; the residue was dissolved in MeOH (∼10 mL), and the complexes were precipitated by the addition of acetone or Et_2_O (∼30 mL) to give the complexes as white powders in 70–80% yields. For characterization of the complexes, see SI (NMR data in Tables S9 and S10 and Figures S4 and S5; MS data in Table S12).

#### Mononuclear Complexes of **L2**

The [1Ln­(**L2**)] complexes (Ln = Eu, Gd) were prepared by mixing **L2**·4HCl·8H_2_O (∼100 mg, 1 equiv) and the corresponding LnCl_3_·*n*H_2_O (∼40 mg, 1 equiv) in water and adjusting the solution pH to 6.5–7.5 by 1% aq. NH_3_. The mixture was stirred at 60 °C for 3 days. Volatiles were evaporated *in vacuo*, and the complexes were purified on a weak cation-exchange resin Amberlite CG50 (H^+^-cycle, 4 cm × 2 cm, elution with water, 3 mL fractions). The pure product-containing fractions were combined, pH was readjusted to 6.5–7.5 by 1% aq. NH_3_, and volatiles were evaporated *in vacuo*. The residue was dissolved in MeOH, and the complexes were precipitated with Et_2_O as white powders, yielding 35–45%. [1Eu­(**L2**)]: ^1^H NMR (600 MHz, D_2_O, 25 °C, pH ∼ 7): δ 9.3, 11.2, 16.7, 20.2 (axial hydrogen atoms of *v*-TSA), 25.8, 32.5, 34.6, 36.5 (axial hydrogen atoms of *v*-SA), −20 to 5 (remaining overlapping signals). ^31^P NMR (243 MHz, D_2_O, 25 °C, pH 11 adjusted by NH_3_) δ ∼ 30 (2 × bs, noncoordinated phosphinate group of the TSA and SA isomers) 68.2 (bs, coordinated phosphinate of the TSA isomer), 78.6 (bs, coordinated phosphinate of the SA isomer). MS­(ESI^+^): *m*/*z* 1011.37, 1009.49 ([^153/151^Eu­(**L2**) + H)]^+^, theor. 1011.25, 1009.25. MS­(ESI^–^): *m*/*z* 1009.22, 1007.26 ([^153/151^Eu­(**L2**)–H)]^−^, theor. 1009.23, 1007.23. [1Gd­(**L2**)]: MS­(ESI^+^): *m*/*z* 1016.13, 1014.33, 1017.64, 1015.25, 1012.71 ([^158/156/160/157^Gd­(**L2**) + H)]^+^, theor. 1016.25, 1014.25, 1018.26, 1015.25, 1013.25. MS­(ESI^–^): *m*/*z* 1014.42, 1012.61, 1016.56, 1013.56, 1011.16 ([^158/156/160/157/^Gd­(**L2**)–H)]^−^, theor. 1014.42, 1012.24, 1016.24, 1013.24, 1011.24.

#### Heterodinucelar Complex [GdY­(**L2**)]

The [1Gd­(**L2**)] complex (∼40 mg, 1 equiv) and YCl_3_·8H_2_O (∼20 mg, 1.1 equiv) were dissolved in water, and the solution pH was adjusted to 6.5–7.5 with 1% aq. NH_3_. The solution was stirred at 60 °C for 3 days. The [GdY­(**L2**)] complex was purified analogously to the homodinuclear complexes and was isolated as a white powder in 70% yield. ^31^P NMR (243 MHz, D_2_O, 25 °C, pH 7): δ 29, 34, 37, 43, 45, 46, and 47 (all very broad signals that could not be assigned to the diastereo­isomers). No signals could be identified by ^1^H NMR. ^89^Y NMR (29.4 MHz, D_2_O, 25 °C, pH 7): δ 114.3 (combined signals of all *v*-TSA isomers) and 115.6 (combined signals of all *v*-SA isomers); see also Figure S10. MS­(ESI^+^): *m*/*z* 1102.12, 1100.05, 1101.24, 1104.12, 1099.28 ([^158/156/157/160/155^Gd^89^Y­(**L2**) + H)]^+^, theor. 1102.14, 1000.13, 1101.14, 1104.14, 1099.13. MS­(ESI^–^): *m*/*z* 1099.97, 1097.83, 1098.89, 1012.12, 1096.00 ([^158/156/157/160/155^Gd^89^Y­(**L2**)–H)]^−^, theor. 1100.12, 1098.12, 1099.12, 1012.12.

### ^89^Y NMR Experiments

The ^89^Y NMR spectra were measured on a Bruker Avance III 600 spectrometer equipped with a broadband probe (29.4 MHz for ^89^Y) at 5 °C and a high sample concentration (approximately 0.1 m) due to the low sensitivity of the ^89^Y nucleus. For diamagnetic samples, a relaxation delay of 5 s was used, and 20,000–30,000 transients were accumulated to get the desired signal-to-noise ratio. For the paramagnetic [GdY­(**L2**)] complex, a relaxation delay of *d*_1_ = 2 s was applied, and 1000 transients were accumulated. The spectra were referenced to a 1-mm Y­(NO_3_)_3_ sample in D_2_O in a coaxial insert tube. The *T*_1_ relaxation times of the ^89^Y nucleus in the [GdY­(**L2**)] complex were determined by a standard inversion recovery pulse sequence with the relaxation delay of *d*_1_ = 0.5 s using 20 values of variable delay in the 0.01–0.25 s range and accumulating 6400 transients at each value. Then, the spectra were phase- and baseline-corrected and fitted to a three-parameter exponential in MestReNova.

### DFT Calculations

The structures of the [GdY­(**L2**)­(H_2_O)_2_]^2–^ and [Y(H_2_O)_*n*_(do3ap^Me^)]^−^ complexes were calculated using Gaussian 16 ^79^ with the wB97XD functional. For all nonlanthanide atoms, the def2-tzvp^80,81^ basis sets were used. For the Gd^iii^ ion, a large core effective potential of the Stuttgart type (the quasi-relativistic ECP53MWB, which includes 46 + 4f^7^ electrons in the core) was used along with a corresponding (6s6p5d)/[4s4p4d] + 2s1p1d basis set.^82,83^ For the Y^iii^ ion, an effective potential (the fully relativistic ECP28MDF, which includes 28 electrons in the core) with the corresponding ECP28MDF_VTZ basis set was used.^84^ To determine possible solution structures, a potential energy surface scan was employed, varying the dihedral angle between the two planes formed by the P–C–P–O atoms. Here, the wB97XD functional was used with a smaller def2-svp basis set. All structures corresponding to local minima on the potential energy surface were then optimized by using a larger def2-tzvp basis set. In all of these calculations, a polarized continuum model was used to account for solvent (water) effects, and the solute cavity was defined by the solvent accessible surface.^85^ All optimized geometries were then analyzed with frequency calculations, and their free energies were determined at the same level of theory.

The ^89^Y NMR shifts of [Y­(H_2_O)_*n*_(do3ap^Me^)]^−^ diastereo­isomers (*n* = 0, 1) were calculated in Orca 6.0,^86^ which incorporates the SHARK integral generation and digestion engine.^87^ Shifts were calculated by DFT using the RIJCOSX^88–90^ approximation with the TPSSh^91^ density functional and the def2-tzvpp basis set recontracted for ZORA calculations for the ligand atoms, and the SARC-zora-TZVPP basis for Y.^92^ The Def/J and SARC/J auxiliary base sets were used in conjunction with the RIJCOSX approximation. The relativistic effects of Y^iii^ were considered by zero-order regular approximation (ZORA). To account for origin-independence, the GIAO method was used.^93^ Solvent effects were included in the calculation by a conductor-like polarization model.^94^ NMR chemical shifts were obtained by calculating the absolute shielding of a Y^iii^ cation surrounded by 24 explicit water molecules reported previously,^95^ calculated at the same level of theory.

### Fluorescence Lifetimes

For fluorescence measurements, solid samples of the Eu^iii^ complexes (∼15 mg) were dissolved in water (1.0 mL) or D_2_O (1.0 mL). For samples in D_2_O, the solvent was evaporated *in vacuo*, and the samples were redissolved in D_2_O and evaporated *in vacuo* three times to remove any residual H_2_O. Then, the pH of the samples was adjusted to pH/pD ∼ 7/7.4 by 0.5% aq. HCl or aq. NaOH and by 0.5% DCl/D_2_O or NaOD/D_2_O for the H_2_O and D_2_O samples, respectively. Fluorescence spectra were measured on an Edinburgh FLS980 (λ_exc_ = 395 nm). The Ln-based fluorescence lifetimes were measured on an Aminco Bowman Series 2 spectrometer after Eu^iii^ excitation at λ_exc_ = 395 nm and at emission band with λ_em_ = 616 nm. The number of coordinated water molecules was calculated using eq 3,^58^ which contains empirical parameters *A* and α, previously determined for various Ln^iii^ ions (*A* = 1.11 ms and α = 0.31 ms^–1^ for Eu^iii^).^58^

3 $$q=A\left(\frac{1}{\tau_{H}}-\frac{1}{\tau_{D}}-\alpha \right)$$

### Relaxivity Determination

Relaxivity was determined by measuring the *T*_1_ relaxation time of the ^1^H water signal in samples of the Gd–**L1** and Gd–**L2** complexes on a Magritek 43 MHz or a Varian VNMR 300 spectrometer. The standard inversion recovery pulse sequence was used with 20 variable delay values in the range of 1 ms to 0.5 s, a repetition time of 2 s, and an acquisition time of 0.4 s. Both complexes were dissolved in deionized water to reach an approximately 1-mm Gd^iii^ concentration. The solution pH was adjusted by 0.1% aq. HCl or aq. NH_3_. The *T*_1_ values were determined by fitting the inversion recovery intensities *I* to eq 4 in MestreNova, where the spectra were also processed.

4 $$I=a+b\cdot \exp\left(-\frac{t}{T_{1}}\right)$$

Relaxivity was calculated from the determined *T*_1_ values using eq 5. The *T*_1_ value of water, *T*_1_(H_2_O), was determined by the same experiment on a sample of an analogous 1-mm La^iii^ complex in water. The exact Gd^iii^ concentration in the samples was determined by the Evans method^96^ using the bulk magnetic susceptibility of the samples (see SI, eq S4).

5 $$r_{1}=\frac{T_{1}-T_{1}\left(H_{2}O\right)}{c\left({\mathrm{Gd}}^{\mathrm{III}}\right)}$$

## Supplementary Material



## Notes

Additional synthetic experimental details, explanation of the complex isomerism, multinuclear NMR and MS data of the complexes, fluorescence data, additional solution dynamics and relaxation data, and characterization data of the complexes (PDF)
