# Supplementary material for: Isomerism and Relaxation Properties of Lanthanide(III) Complexes of a Ditopic Ligand with Two DO3A Units Bridged by a Methylene-bis(phosphinate) Spacer
Source: Inorg Chem. 2026 May 12;65(20):10993–1006. doi: 10.1021/acs.inorgchem.6c00452 (PMC13213905; doi:10.1021/acs.inorgchem.6c00452)
Supplement: Supplementary file 1 [file ic6c00452_si_001.pdf]

Supporting Information

for

Isomerism and relaxation properties of lanthanide(III) complexes of a ditopic ligand with two DO3A units bridged by a methylene-bis(phosphinate) spacer

Adam Svítok,[a] Carlos Platas-Iglesias,[b] Petr Hermann[a]*

[a]Department of Inorganic Chemistry, Faculty of Science, Charles University, Hlavova 2030, 12843 Prague 2, Czech Republic. petrh@natur.cuni.cz

[b]Universidade da Coruña, Centro Interdisciplinar de Química e Bioloxía (CICA) and Departamento de Química, Facultade de Ciencias, 15071, A Coruña, Spain.

Content:

Solution isomerism S3

Figure S1. Possible isomers of [Ln(do3ap^{R})] complexes S3

Table S1. Possible diastereoisomers of [2Ln(**L2**)] S4

NMR spectra of complexes S5

Figure S2. The ^{1}H NMR spectra of [Ln(**L1**)] S5–S7

Figure S3. The ^{31}P NMR spectra of [Ln(**L1**)] S8–S10

Figure S4. The ^{1}H NMR spectra of [2Ln(**L2**)] S11–S12

Figure S5. The ^{31}P NMR spectra of [2Ln(**L2**)] S13–S15

Fluorescence lifetimes of Eu^{III} complexes S16

Figure S6. The fit of the dependence of fluorescence intensity on time for [2Eu(**L2**)] S16

Figure S7. The fit of the dependence of fluorescence intensity on time for [Eu(**L1**)] S16

Table S2. Comparison of luminescence lifetimes of complexes. S16

Figure S8. Structure of ligands discussed in Table S2. S17

Figure S9. Fluorescence spectra of Eu(III) complexes. S17

^{89}Y NMR studies S18

Table S3. Calculated chemical shifts (DFT) of diastereoisomers of the [Y(do3ap^{Me})] S18

Figure S10. The ^{89}Y NMR spectra of [Y(do3ap^{Me})], [2Y(**L2**)] and [GdY(**L2**)] S18

Determination of the rotational correlation time S19

Table S4. The NOE factors, ^{13}C NMR R_1 relaxation times and τ_R of [2Y(**L2**)] S19

Figure S11. Calculated geometries for diastereoisomers of the [GdY(**L2**)] S20

Table S5. The DFT-calculated energies for structures of [GdY(**L2**)] diastereoisomers S21

Solution dynamics S22

Figure S12. The 2D ^{1}H–^{1}H EXSY of [Eu(**L1**)] S22

Figure S13. An example of chemical exchange by macrocyclic inversion in the [2Ln(**L2**)] S23

Figure S14. The 2D ^{31}P–^{31}P EXSY of the [2Eu(**L2**)] S23

Figure S15. Variable-temperature ^{31}P NMR spectra of the [2Eu(**L2**)] S24
Figure S16. The 2D ^{1}H–^{1}H EXSY of the [2Ce(**L2**)] S24
Relaxivity of the Gd^{III} complexes S25
Determination of the Gd^{III} concentration by Evans method S25
Figure S17. Relaxivities of the Gd^{III} complexes of **L1** and **L2** at different pH S25
Experimental data S26
Notes on the synthesis of **L1** S26
Synthesis of diisopropyl methylene-bis(*H*-phosphinate) **1** S26
Characterization of complexes S27
Table S6. Acquisition times and relaxation delays used to acquire the paramagnetic NMR spectra S27
Table S7. The ^{1}H NMR chemical shifts of the paramagnetic [Ln(**L1**)] S27
Table S8. The ^{31}P NMR chemical shifts of the [Ln(**L1**)] S28
Table S9. The ^{1}H NMR chemical shifts of some paramagnetic [2Ln(**L2**)] S28
Table S10. The ^{31}P NMR chemical shifts of the [2Ln(**L2**)] S29
Table S11. Values of *m*/*z* of the most intensive signals in mass spectra of the [Ln(**L1**)] S30
Table S12. Values of *m*/*z* of the most intensive signals in mass spectra of the [2Ln(**L2**)] S31–S33
References S34

Solution isomerism

The diastereoisomers result from a combination of three centres of chirality: macrocycle conformations, λλλλ/δδδδ, pendant arm orientations, Λ/Δ, and phosphorus atom configurations, *R*/*S*. Each diastereoisomer forms an enantiomeric pair. All possibilities are shown in Figure S1.

Figure S1. Possible isomers of the [Ln(do3ap^{R})] complexes (R = (substituted)alkyl) for major (*vertical*) and minor (*horizontal*) phosphorus atom configuration with different macrocycle conformations and pendant arms orientations.

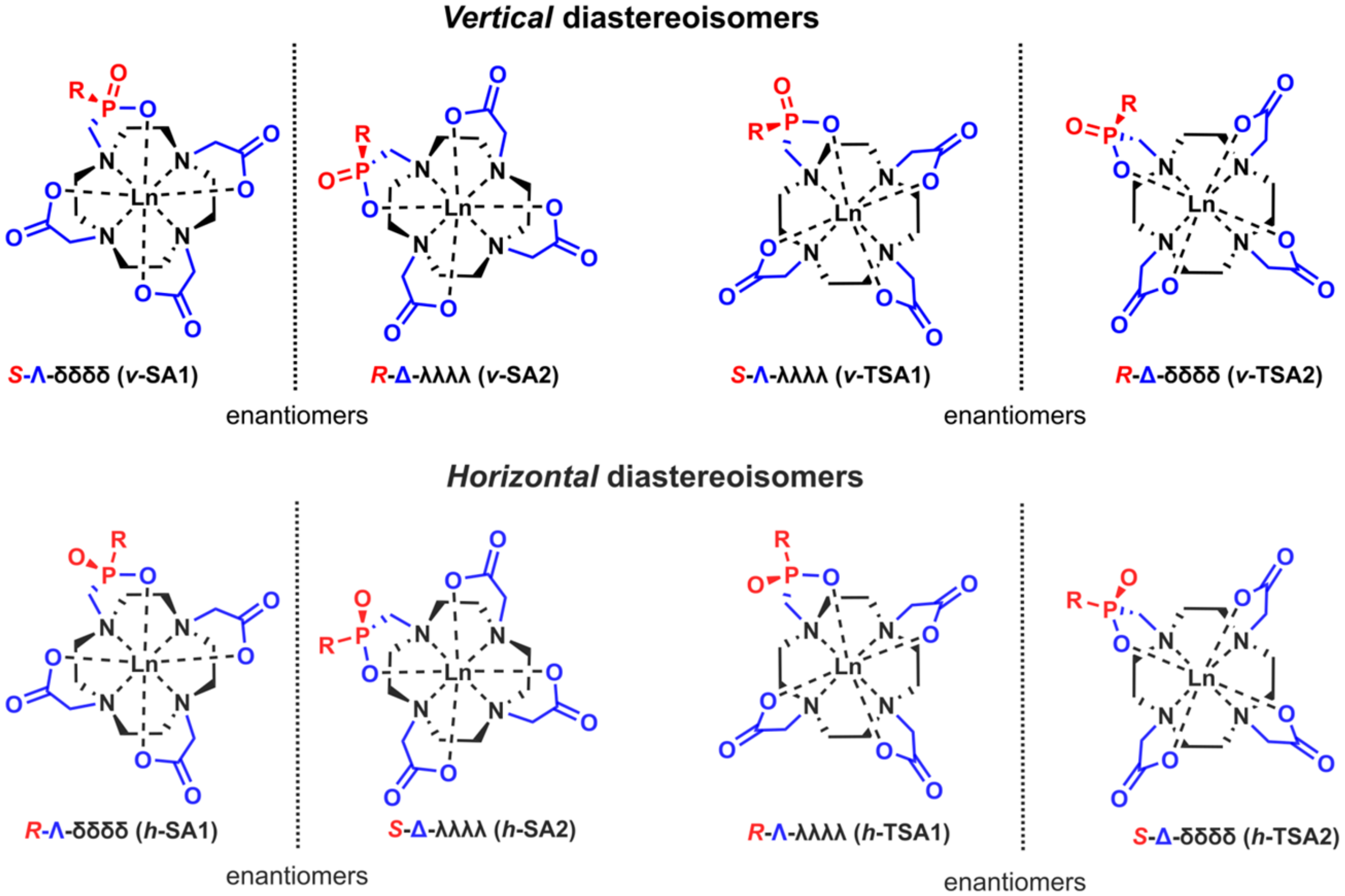

Table S1. Possible diastereoisomers of homo-dicomplexes of ligand **L2**, [2Ln(**L2**)], formed by combination of different subunit geometries; only *vertical* phosphorus atom configuration is considered (see also Figure 2).

Subunit 1	Subunit 2	Diastereoisomer[a]	**Label**
TSA1(*S*-Λλλλλ)<br>TSA2 (*R*-Δδδδδ)	TSA2 (*R*-Δδδδδ)<br>TSA1(*S*-Λλλλλ)	TSA1–TSA2 = TSA2–TSA1<br>(*S*-Λλλλλ–*R*-Δδδδδ) = (*R*-Δδδδδ –*S*-Λλλλλ)	***df*-TSA**
TSA1(*S*-Λλλλλ)<br>TSA2 (*R*-Δδδδδ)	TSA1(*S*-Λλλλλ)<br>TSA2 (*R*-Δδδδδ)	TSA1–TSA1 ≈ TSA2–TSA2<br>(*S*-Λλλλλ– *S*-Λλλλλ) ≈ (*R*-Δδδδδ–*R*-Δδδδδ)	**sm-TSA**
TSA1(*S*-Λλλλλ)<br>TSA2 (*R*-Δδδδδ)	SA1 (*S*-Λδδδδ)<br>SA2 (*R*-Δλλλλ)	TSA1–SA1 ≈ TSA2–SA2<br>(*S*-Λλλλλ– *S*-Λδδδδ) ≈ (*R*-Δδδδδ–*R*-Δλλλλ)	***arm*-TSA–SA**
TSA1(*S*-Λλλλλ)<br>TSA2 (*R*-Δδδδδ)	SA2 (*R*-Δλλλλ)<br>SA1 (*S*-Λδδδδ)	TSA1–SA2 ≈ TSA2–SA1<br>(*S*-Λλλλλ–*R*-Δλλλλ) ≈ (*R*-Δδδδδ–*S*-Λδδδδ)	***cyc*-TSA–SA**
SA1 (*S*-Λδδδδ)<br>SA2 (*R*-Δλλλλ)	SA1 (*S*-Λδδδδ)<br>SA2 (*R*-Δλλλλ)	SA1–SA1 ≈ SA2–SA2<br>(*S*-Λδδδδ–*S*-Λδδδδ) ≈ (*R*-Δλλλλ–*R*-Δλλλλ)	***sm*-SA**
SA1 (*S*-Λδδδδ)<br>SA2 (*R*-Δλλλλ)	SA2 (*R*-Δλλλλ)<br>SA1 (*S*-Λδδδδ)	SA1–SA2 = SA2–SA1<br>(*S*-Λδδδδ–*R*-Δλλλλ) = (*R*-Δλλλλ–*S*-Λδδδδ)	***df*-SA**

[a]Combinations corresponding to **one** diastereoisomer: "=" means fully equivalent structures (*meso*-forms, *df*-isomers) and "≈" denotes enantiomers (*sm*-, *arm*- and *cyc*-isomers).

Only the major phosphorus configuration (*vertical*) was considered as the minor diastereoisomers with the *horizontal* configuration were not present/observed in solution. The labels correspond to relationships between the subunits: *sm*-SA and *sm*-TSA correspond to diastereoisomers combining two subunits with the ***same*** macrocycle conformation, pendant arms orientation and phosphorus atom configuration; *df*-SA and *df*-TSA correspond to diastereoisomers combining two SA or TSA subunits, respectively, with a ***different***/opposite macrocycle conformation, pendant arms orientation and phosphorus atom configuration. The mixed diastereoisomers containing one SA and one TSA subunit are labelled *arm*-TSA–SA and *cyc*-TSA–SA; *arm*- and *cyc*- mark isomers with the same pendant **arms orientations** and the same **macrocycle conformations**, respectively.

NMR spectra of complexes

*Complexes of **L1**.*

The ^{1}H NMR spectra of [Ln(**L1**)] were measured at 600 MHz, pD ~7 and T = 278.2 K. For paramagnetic complexes, only spectra of the "axial" hydrogen atoms, the closest ones to the pseudo-C_4 axis of the complexes, are shown; these protons have the highest lanthanide-induced shifts (LIS) and are the most distinguishable (for more information, see SI of Ref.[1]). Signals were assigned to the major *v*-SA /*v*-TSA diastereoisomers, assuming an analogy between the ^{1}H NMR spectra of the [Ln(**L1**)] complexes and those of the [Ln(do3ap^{Me})] complexes.[2] However, the *h*-SA diastereoisomer has a very low abundance in the title complexes, and its ^{1}H NMR signals could not be identified in any of the complexes. Similarly, signals of the low-abundant *h*-TSA isomer could not be identified in most of the complexes. For the Sm^{III} complex, the entire spectrum is shown, but signals of the "axial" hydrogen atoms could not be assigned due to a combination of small LIS values and similar abundances of the two major diastereoisomers.

Figure S2. Selected regions of ^{1}H NMR spectra of the [Ln(**L1**)] complexes.

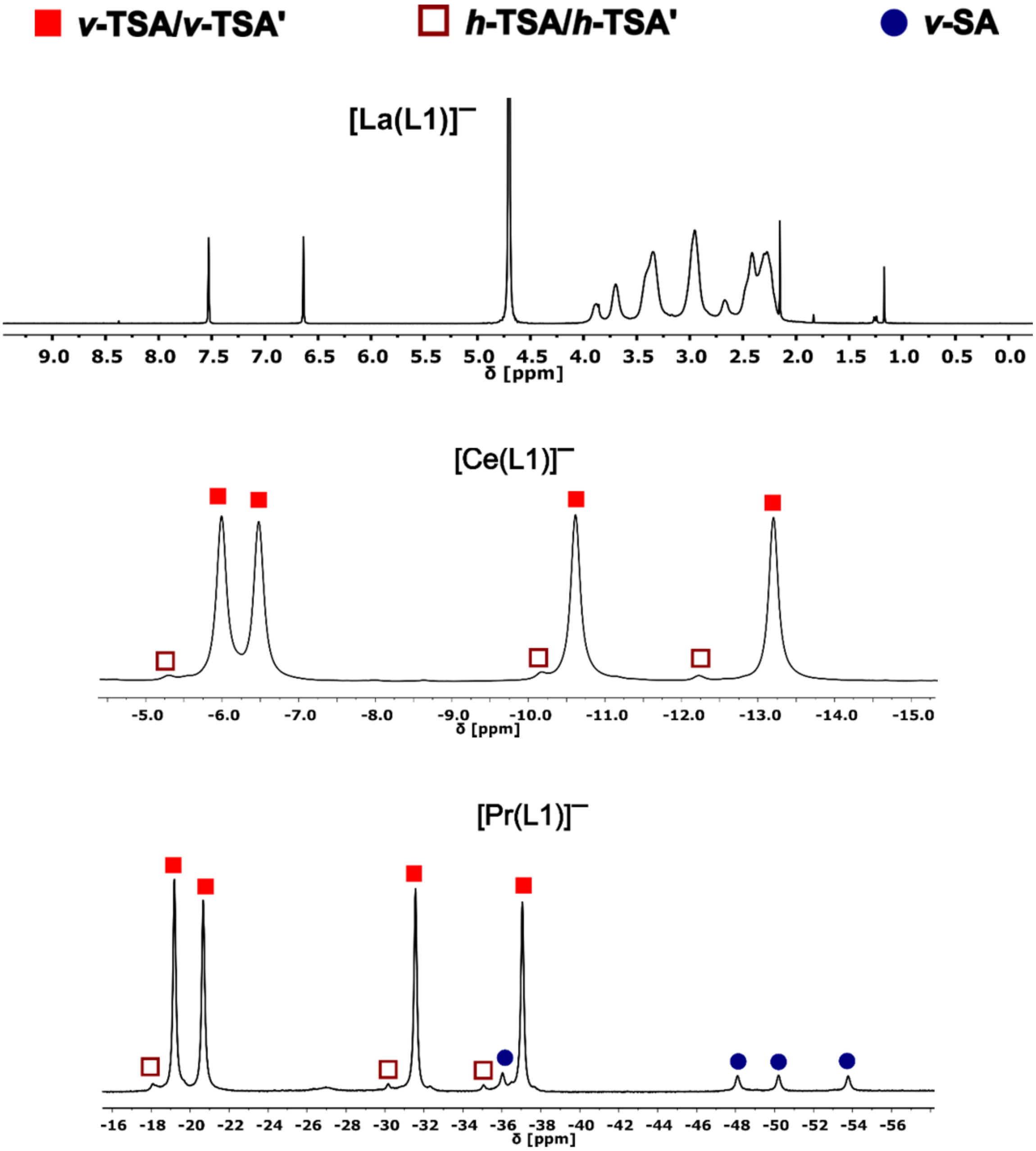

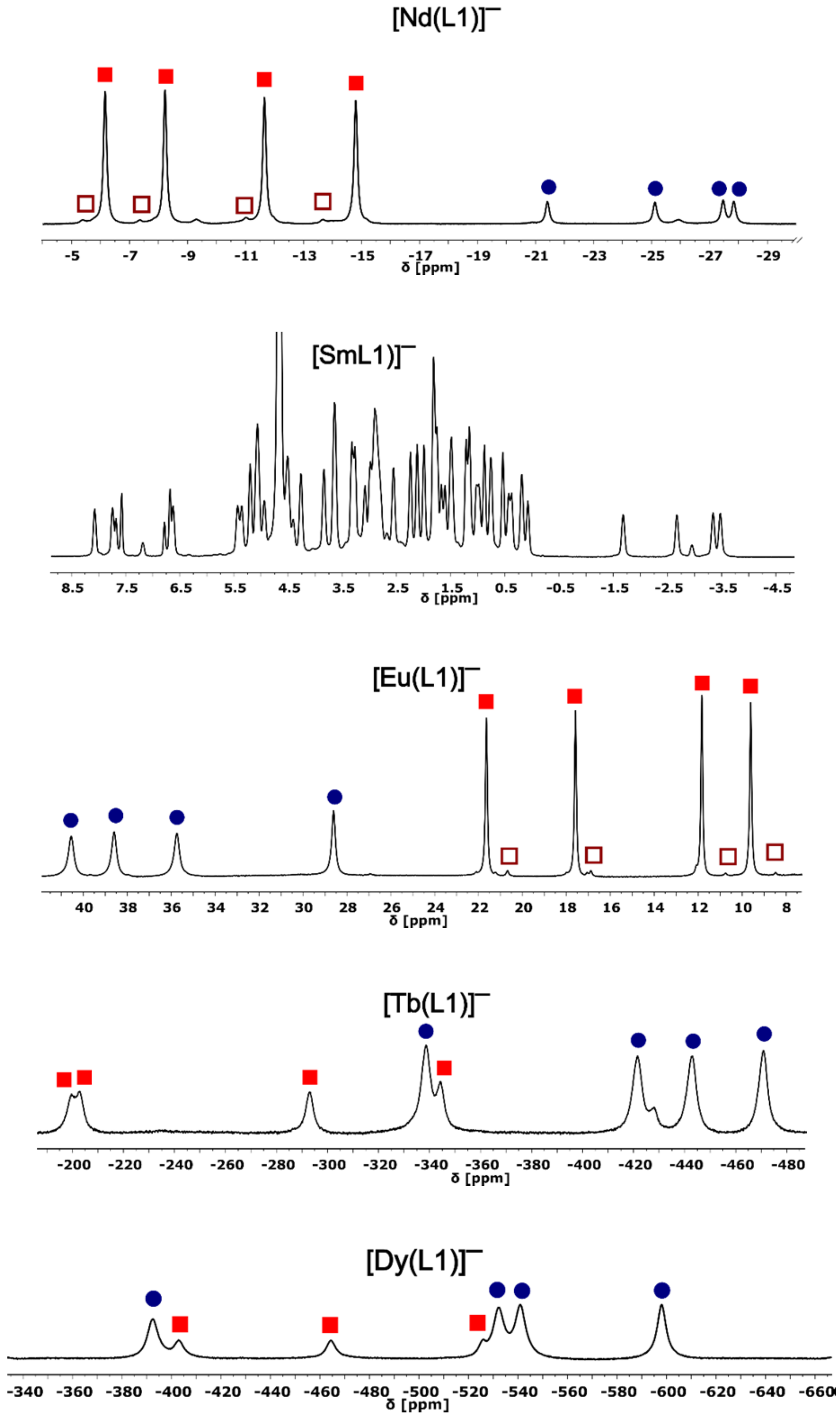

[Nd(L1)]–
δ [ppm]
[SmL1)]–
δ [ppm]
[Eu(L1)]–
δ [ppm]
[Tb(L1)]–
δ [ppm]
[Dy(L1)]–
δ [ppm]

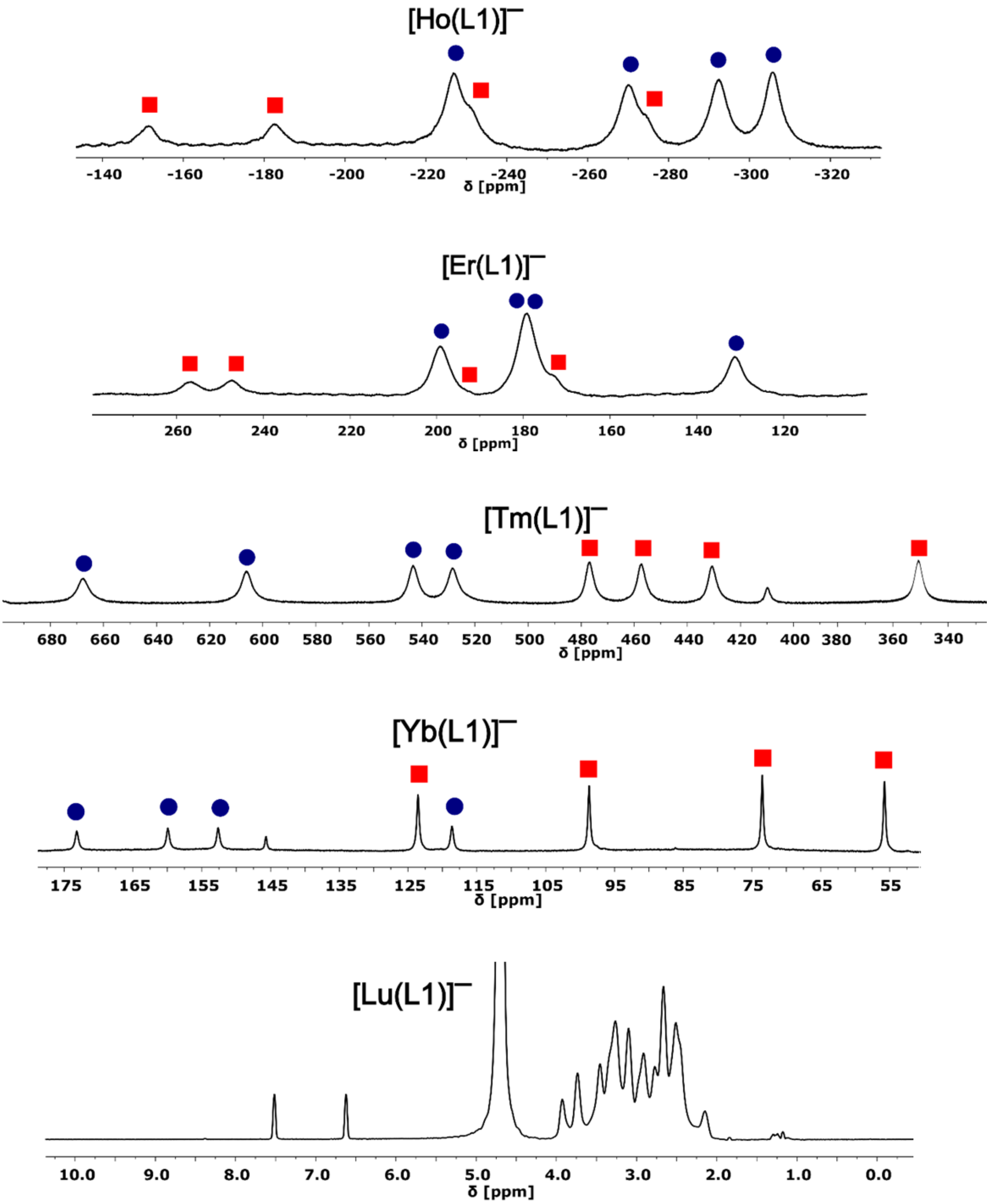
[Ho(L1)]⁻
-140 -160 -180 -200 -220 -240 -260 -280 -300 -320
δ [ppm]
[Er(L1)]⁻
260 240 220 200 180 160 140 120
δ [ppm]
[Tm(L1)]⁻
680 660 640 620 600 580 560 540 520 500 480 460 440 420 400 380 360 340
δ [ppm]
[Yb(L1)]⁻
175 165 155 145 135 125 115 105 95 85 75 65 55
δ [ppm]
[Lu(L1)]⁻
10.0 9.0 8.0 7.0 6.0 5.0 4.0 3.0 2.0 1.0 0.0
δ [ppm]

The ^{31}P NMR spectra were measured at 243 MHz, pD ~7 and T = 278.2 K. Signals of the phosphorus nucleus of the coordinated phosphinate groups were assigned to the major *v*-SA/*v*-TSA diastereoisomers by assuming an analogy between the ^{1}H NMR spectra of the [Ln(**L1**)] and [Ln(do3ap^{Me})] complexes.[2] The doublets correspond to signals of phosphorus groups with a P–H bond and were assigned by comparing their relative integral intensities. For most complexes, signals for *h*-TSA and *h*-SA with very low abundance could not be identified.

Figure S3. The ^{31}P NMR spectra of the [Ln(**L1**)] complexes.

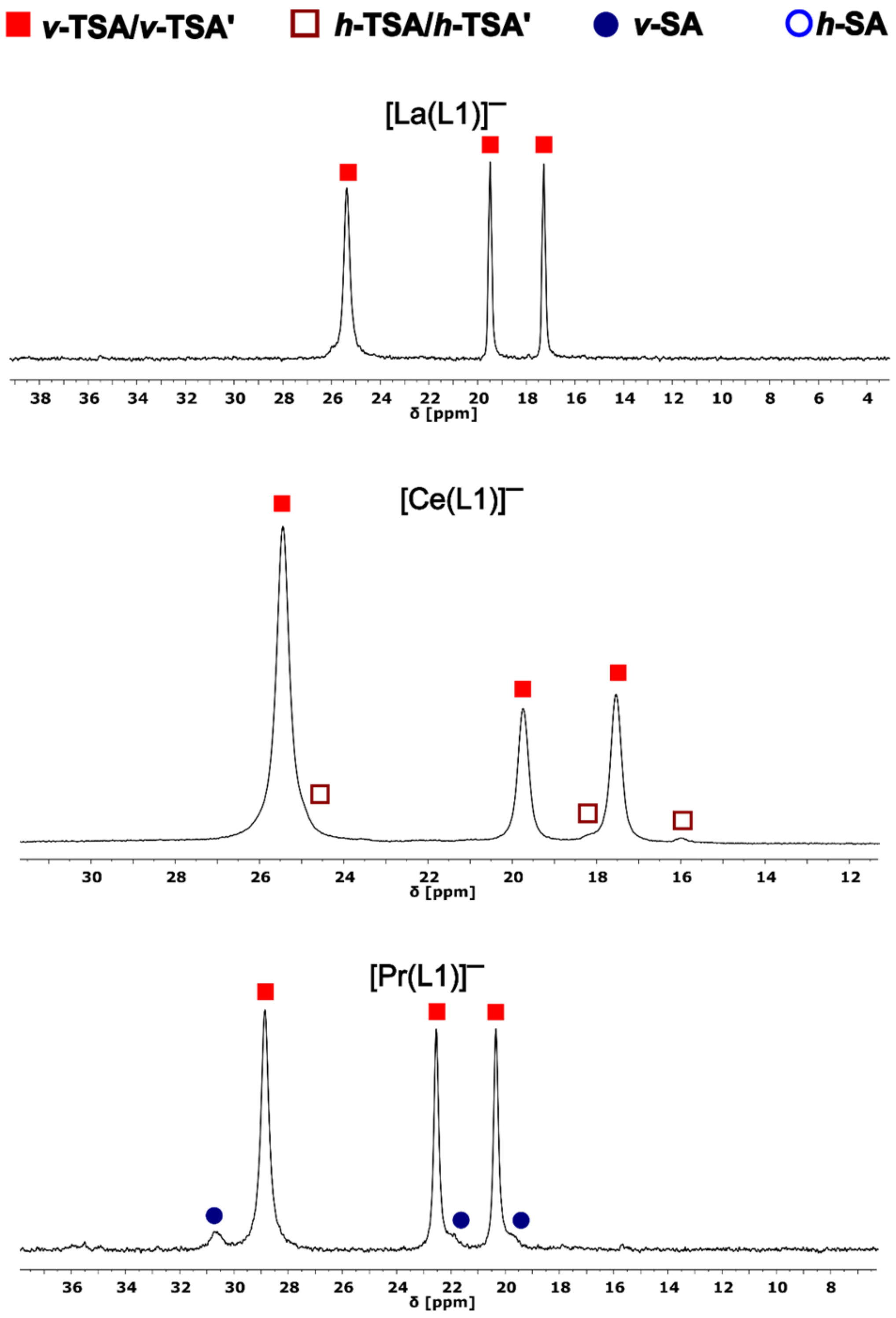

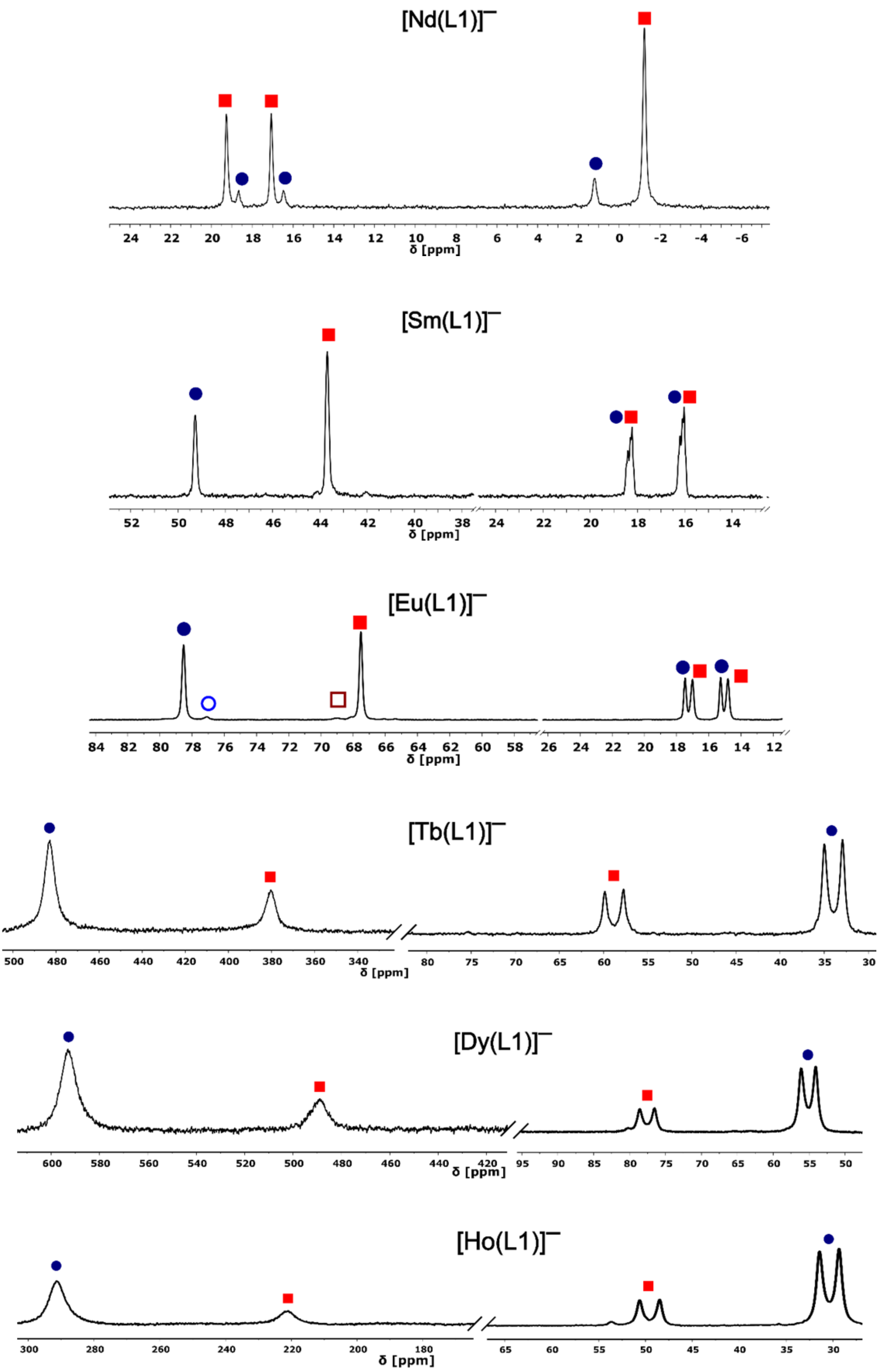
[Nd(L1)]⁻
δ [ppm]
[Sm(L1)]⁻
δ [ppm]
[Eu(L1)]⁻
δ [ppm]
[Tb(L1)]⁻
δ [ppm]
[Dy(L1)]⁻
δ [ppm]
[Ho(L1)]⁻
δ [ppm]

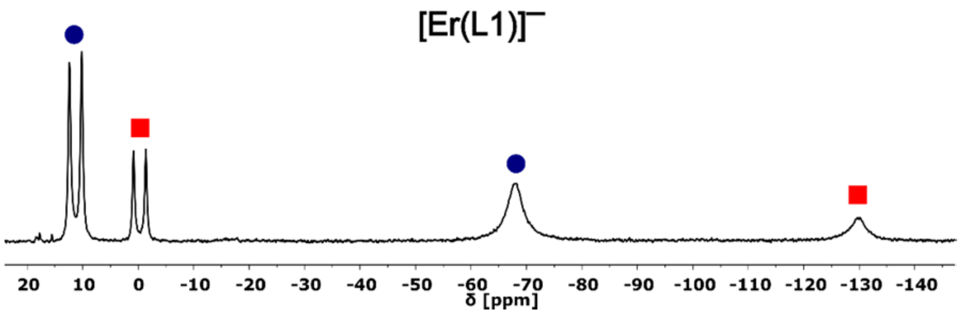
[Er(L1)]⁻
20 10 0 -10 -20 -30 -40 -50 -60 -70 -80 -90 -100 -110 -120 -130 -140
δ [ppm]

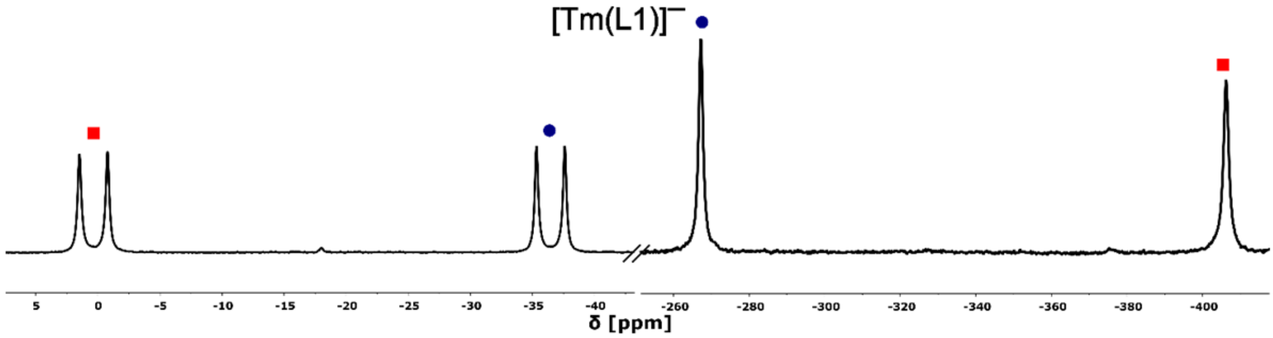
[Tm(L1)]⁻
5 0 -5 -10 -15 -20 -25 -30 -35 -40 -260 -280 -300 -320 -340 -360 -380 -400
δ [ppm]

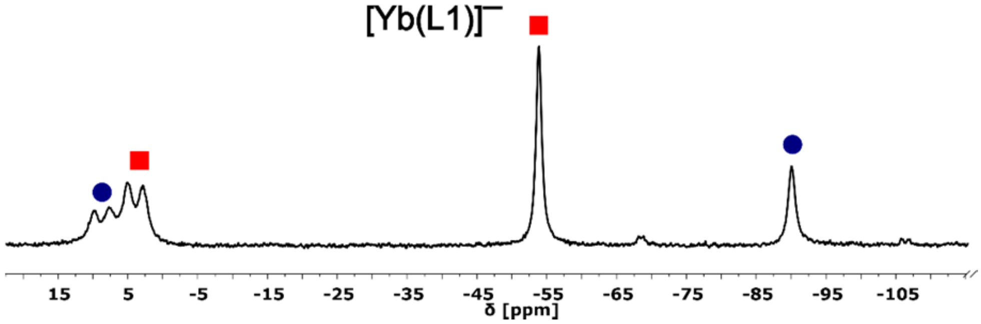
[Yb(L1)]⁻
15 5 -5 -15 -25 -35 -45 -55 -65 -75 -85 -95 -105
δ [ppm]

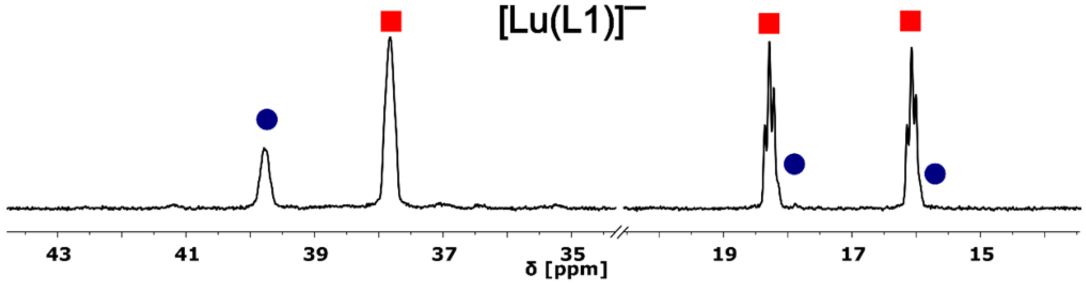
[Lu(L1)]⁻
43 41 39 37 35 19 17 15
δ [ppm]

Complexes of ***L2***.

The ^{1}H NMR spectra of the [2Ln(**L2**)] complexes were measured at 600 MHz, pD ~7 and T = 278.2 K. For the paramagnetic complexes, only spectra of the "axial" hydrogen atoms closest to the pseudo-C_4 axis are shown. Signals were assigned to the *v*-SA and *v*-TSA diastereoisomers by assuming an analogy with the ^{1}H NMR spectra of the [Ln(**L1**)] complexes and to the *sm*/*df*-TSA-TSA, *sm*/*df*-TSA and *arm*/*cyc*-TSA-SA isomers by comparing relative integral intensities of the signals and, in some cases, by comparing the spectra with ^{1}H–^{1}H EXSY or VT NMR spectra (where TSA abundance increases with temperature). Diastereoisomers in each pair of *sm*/*df*-TSA-TSA, *sm*/*df*-TSA or *arm*/*cyc*-TSA-SA could not be distinguished. For complexes of the strongly paramagnetic Tb^{III}, Gd^{III}, Dy^{III}, Ho^{III}, Er^{III} and Tm^{III} ions, spectra are not shown as the signals were too broad and could not be distinguished from baseline distortions. For the Sm^{III} complex, the whole spectrum is shown, but signals of "axial" hydrogen atoms could not be assigned.

Figure S4. Parts of ^{1}H NMR spectra of the [2Ln(**L2**)] complexes.

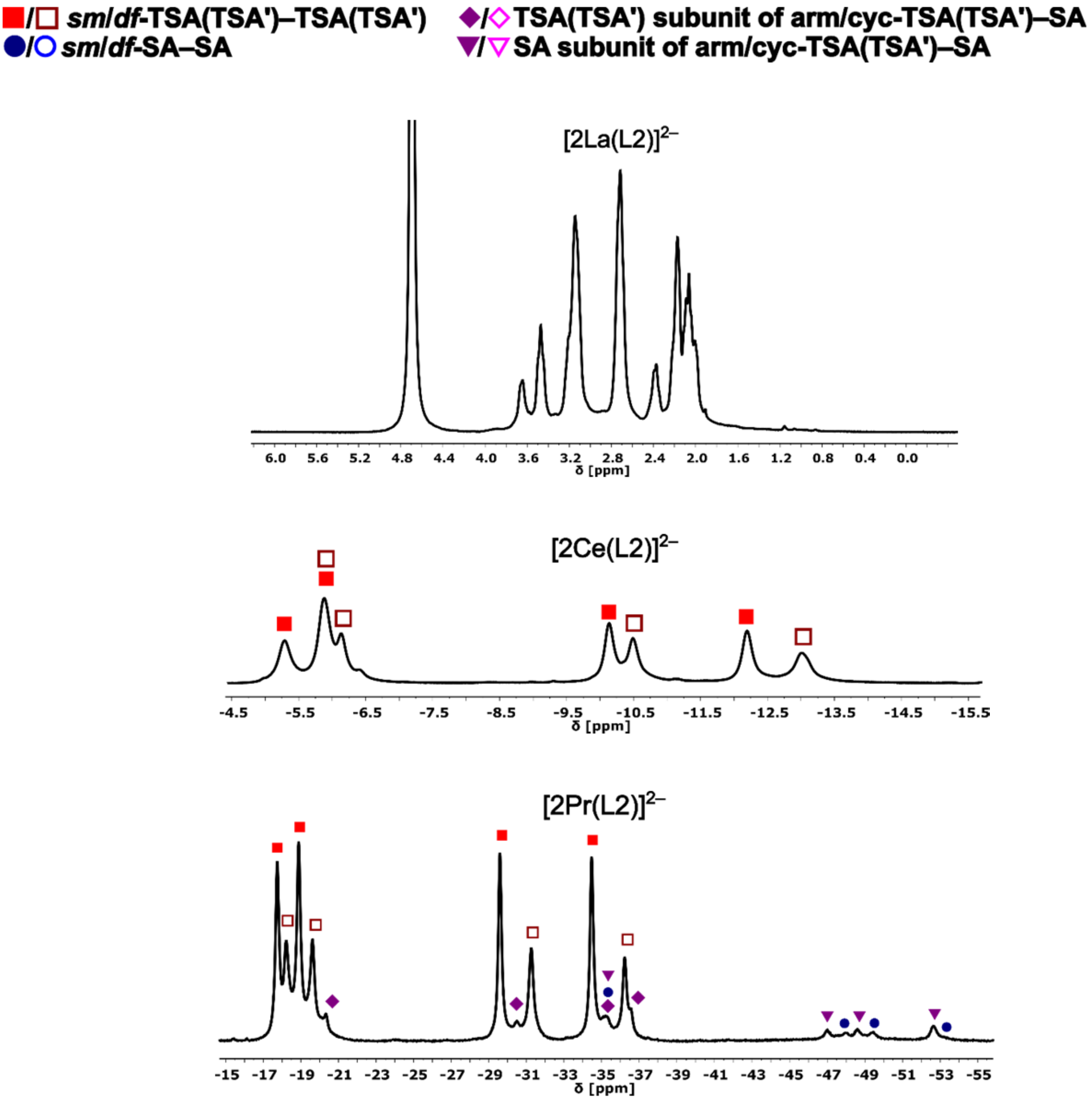

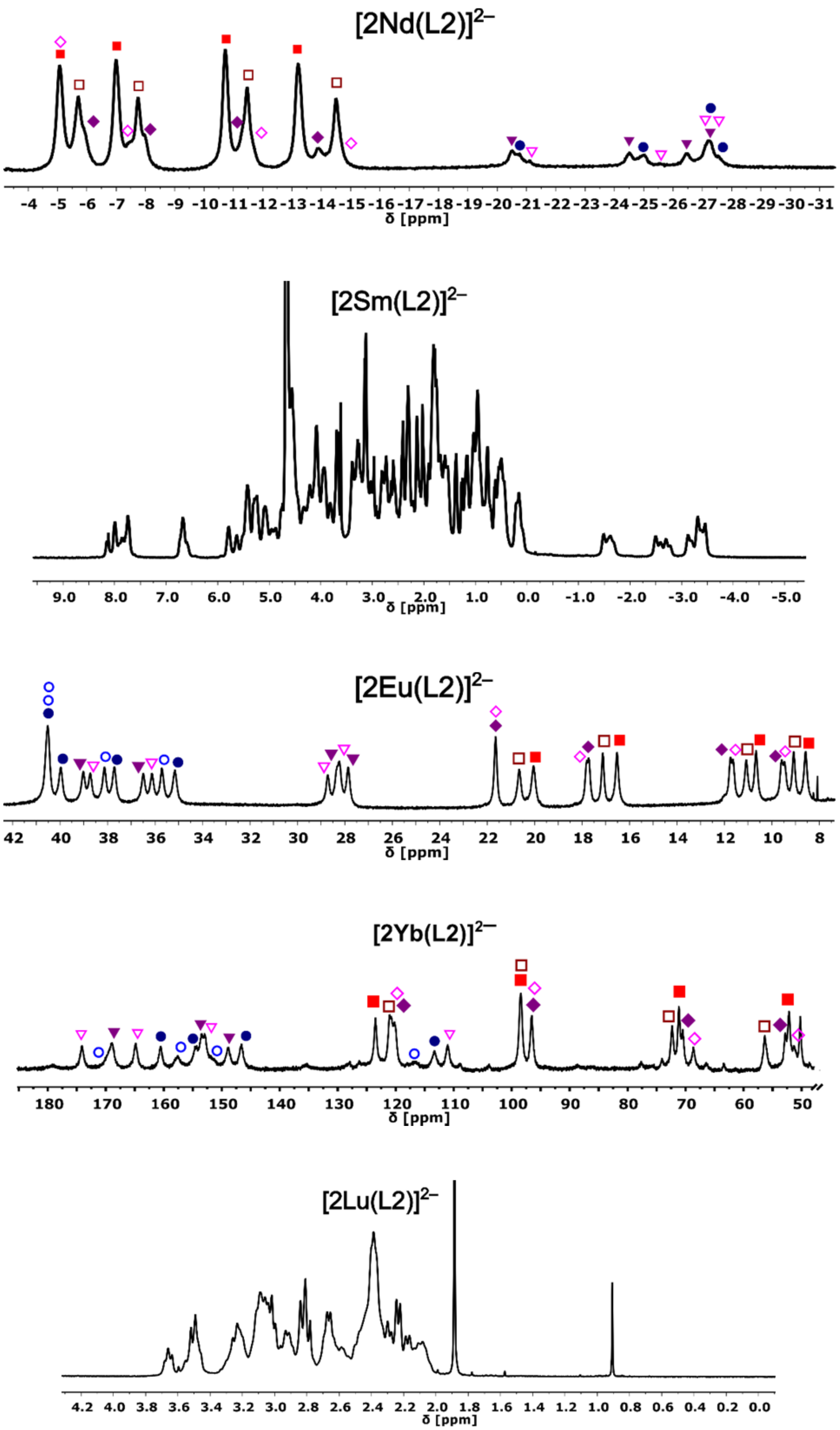
[2Nd(L2)]2–
-4 -5 -6 -7 -8 -9 -10 -11 -12 -13 -14 -15 -16 -17 -18 -19 -20 -21 -22 -23 -24 -25 -26 -27 -28 -29 -30 -31
δ [ppm]
[2Sm(L2)]2–
9.0 8.0 7.0 6.0 5.0 4.0 3.0 2.0 1.0 0.0 -1.0 -2.0 -3.0 -4.0 -5.0
δ [ppm]
[2Eu(L2)]2–
42 40 38 36 34 32 30 28 26 24 22 20 18 16 14 12 10 8
δ [ppm]
[2Yb(L2)]2–
180 170 160 150 140 130 120 110 100 90 80 70 60 50
δ [ppm]
[2Lu(L2)]2–
4.2 4.0 3.8 3.6 3.4 3.2 3.0 2.8 2.6 2.4 2.2 2.0 1.8 1.6 1.4 1.2 1.0 0.8 0.6 0.4 0.2 0.0
δ [ppm]

The ^{31}P NMR spectra were measured at 243 MHz, pD ~7 and T = 278.2 K. Signals were assigned to the *v*-SA and *v*-TSA diastereoisomers by assuming an analogy with ^{31}P NMR spectra of the [Ln(**L1**)] complexes and to *sm*/*df*-TSA-TSA, *sm*/*df*-TSA and *arm*/*cyc*-TSA-SA isomers by comparing relative integral intensities of the signals with relative integral intensities of ^{1}H NMR signals. In some complexes, signals from isomers with lower abundance could not be detected, likely due to overlap with those of more abundant isomers. For the Tb^{III}, Dy^{III} and Ho^{III} complexes, the overlapping broad signal could only be assigned to the SA/TSA geometry but not to a particular diastereoisomer.

Figure S5. The ^{31}P NMR spectra of the [2Ln(**L2**)] complexes.

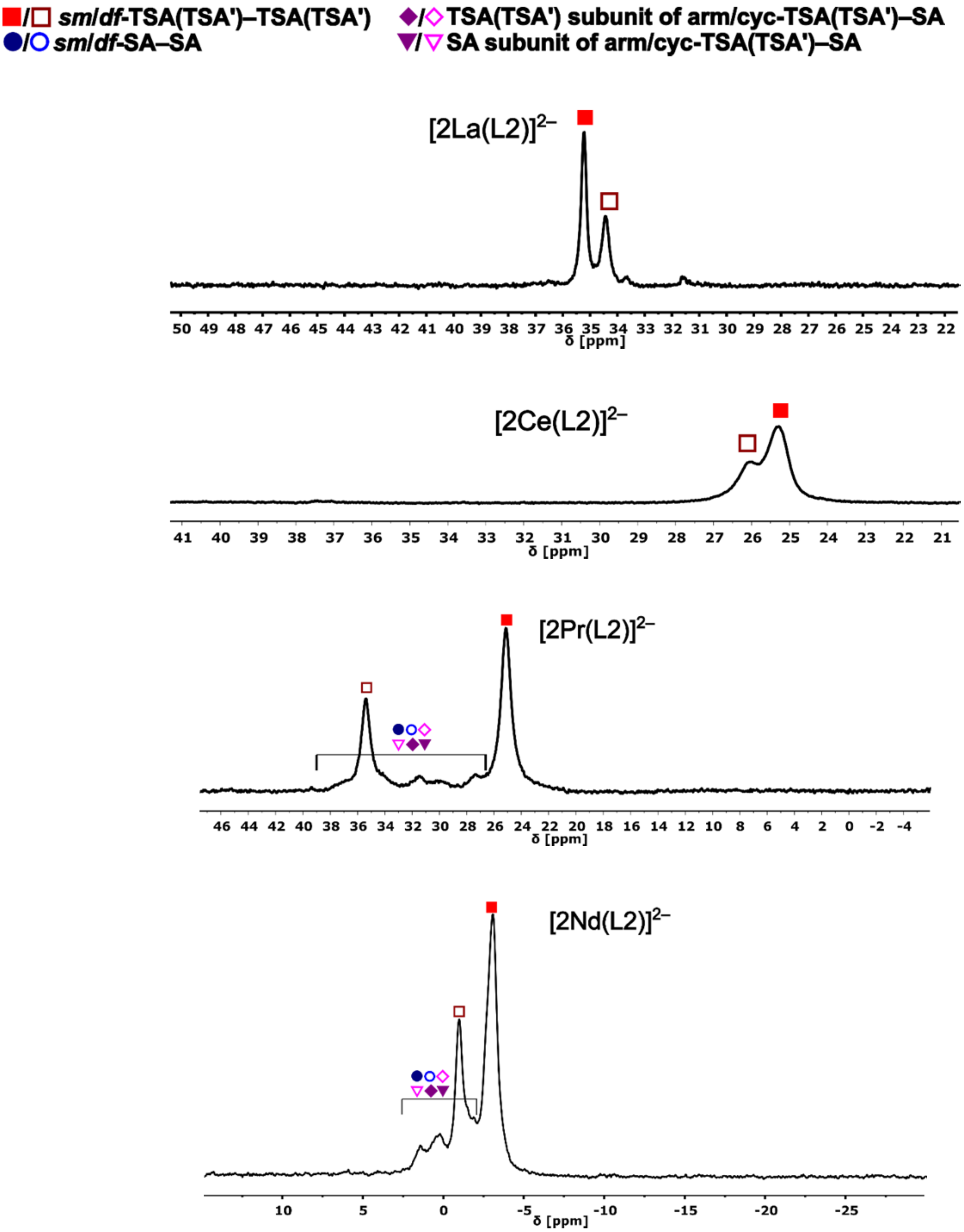

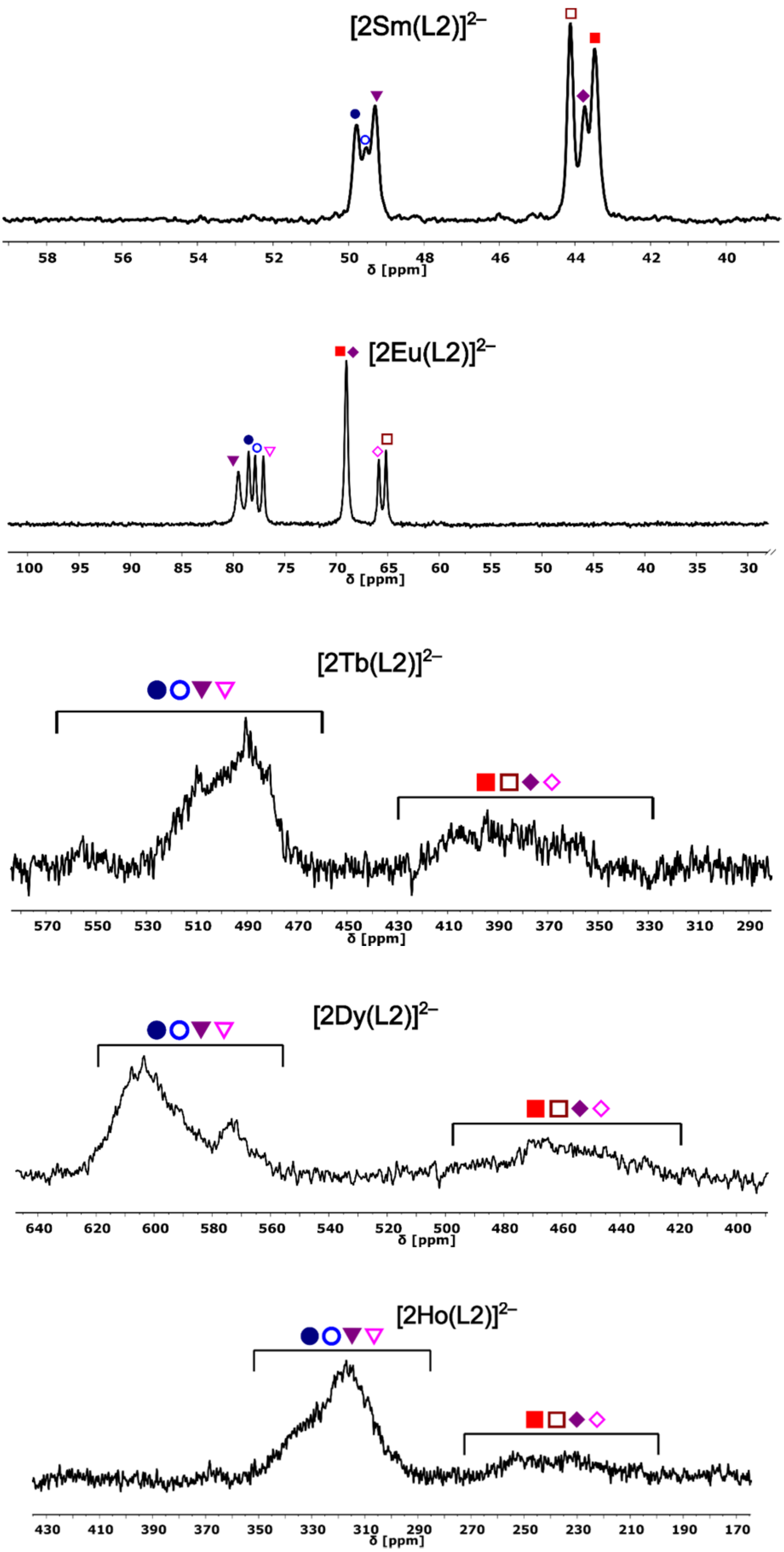
[2Sm(L2)]2–
δ [ppm]
[2Eu(L2)]2–
δ [ppm]
[2Tb(L2)]2–
δ [ppm]
[2Dy(L2)]2–
δ [ppm]
[2Ho(L2)]2–
δ [ppm]

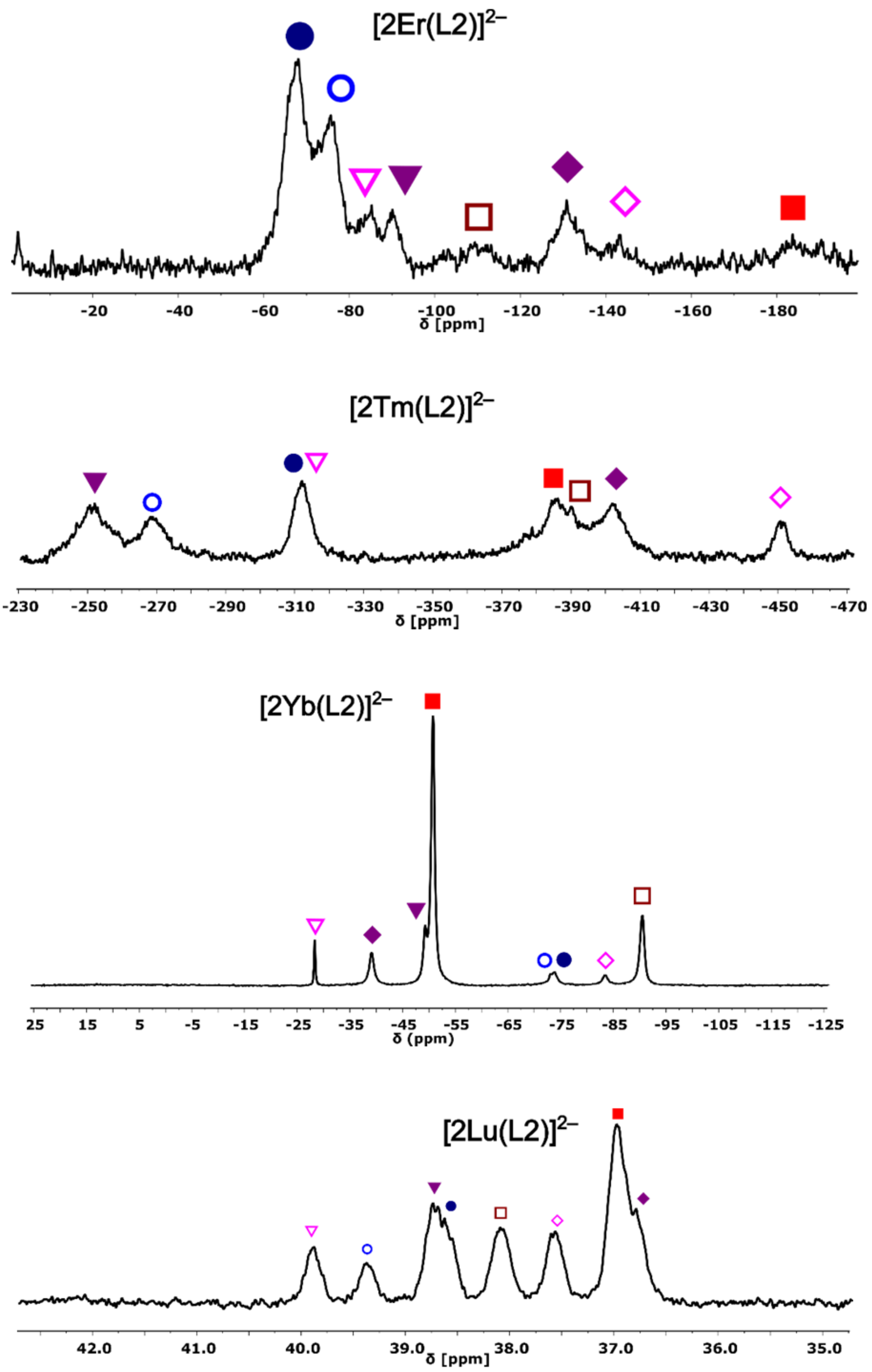
[2Er(L2)]$^{2-}$
-20 -40 -60 -80 -100 -120 -140 -160 -180
δ [ppm]
[2Tm(L2)]$^{2-}$
-230 -250 -270 -290 -310 -330 -350 -370 -390 -410 -430 -450 -470
δ [ppm]
[2Yb(L2)]$^{2-}$
25 15 5 -5 -15 -25 -35 -45 -55 -65 -75 -85 -95 -105 -115 -125
δ (ppm)
[2Lu(L2)]$^{2-}$
42.0 41.0 40.0 39.0 38.0 37.0 36.0 35.0
δ [ppm]

Fluorescence of Eu^{III} complexes

Figure S6. The best fit of the dependence of fluorescence intensity on time that was used to determine the excited-state lifetime for the [2Eu(**L2**)] at 25 °C.

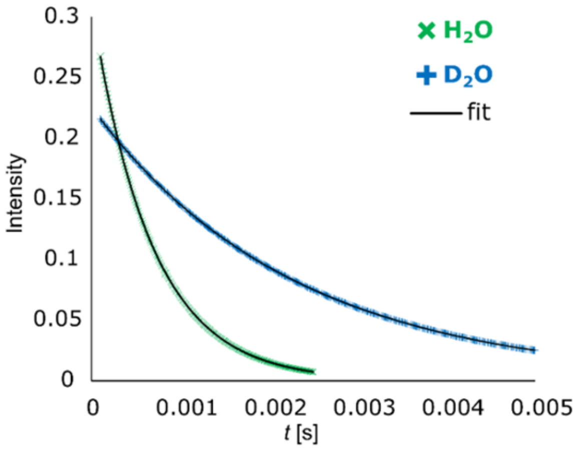

Figure S7. The best fit of the dependence of fluorescence intensity on time that was used to determine the excited-state lifetime for the [Eu(**L1**)] complex at 25 °C.

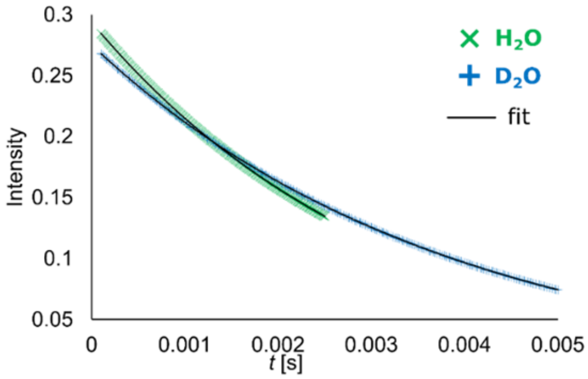

Comparison with similar systems

For the [2Eu(**L2**)] homo-dicomplex, the measured Eu^{III} luminescence lifetime is 0.667 ms. As is seen in Table S2, this value is close to those measured for Eu^{III} complexes of DOTA (0.637 ms)[3] and its monoethyl/diethylphosphonate (0.645/0.625 ms)[4] and tris(methylphosphinate) (0.68–0.76 ms)[5] derivatives. A similar value of excited state lifetime was also determined for a dimeric system consisting of two DO3A subunits bridged by piperazine (0.630 ms), where each Eu^{III} ion binds one water molecule[6].

Table S2. Comparison of luminescence lifetimes in the Eu^{III} complexes of **L1**, **L2** and other DOTA derivatives. Structures of the ligands are shown in Figure S8.

Ligand	**L1**	**L2**^{a}	DOTA	DO3AP$^{(OEt)x}$	DO3P^{Me}AM$^{RR'}$	pip-(DO3A)$_2$^{a}
τ_H [ms]	1.63	0.667	0.637[3]	(x = 1) 0.645 [4]<br>(x = 2) 0.625 [4]	(R = 1-$C_{10}H_7$, R' = Me) 0.73 [5]<br>(R = H, R' = H) 0.76 [5]<br>(R = Ph, R' = Me) 0.68 [5]	0.630 [6]

^{a}Dimeric homodicomplexes with two coordinated Eu^{III}.

Figure S8. Structures of DOTA-based ligands, whose Eu^{III} complexes are discussed in Table S2.

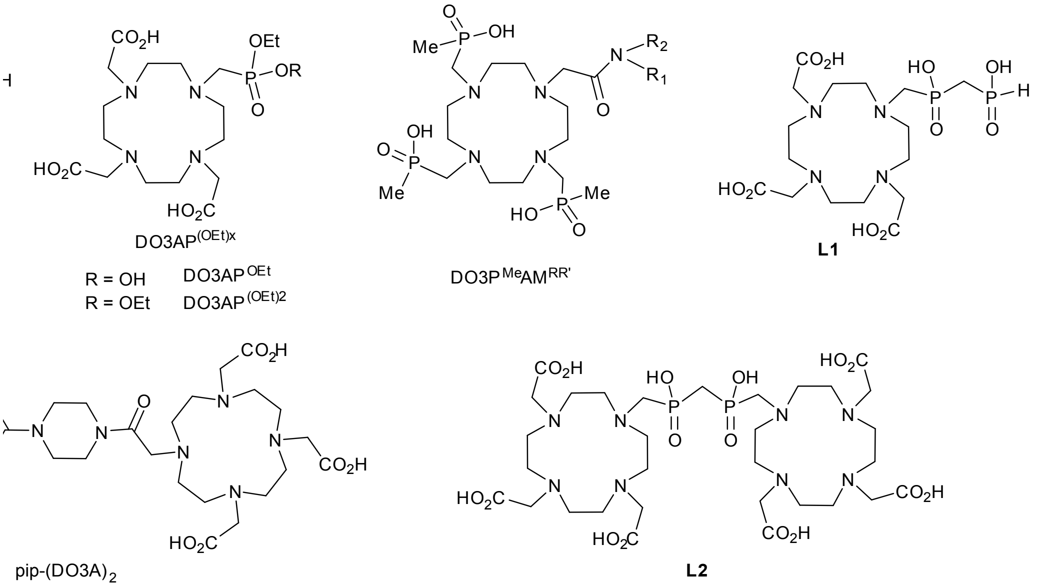

Figure S9. Flurorescence spectra of Eu^{III} complexes.

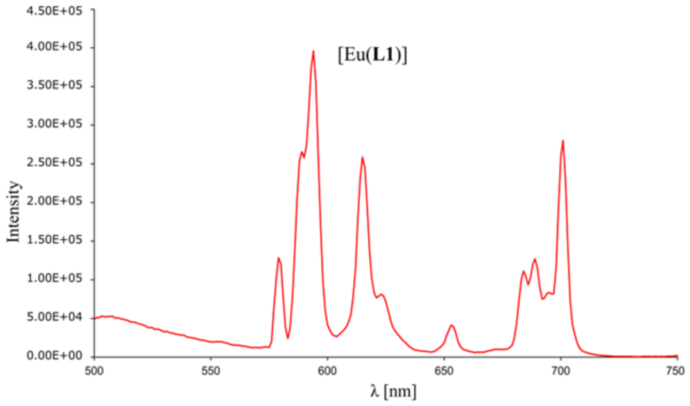

[Eu(**L1**)]

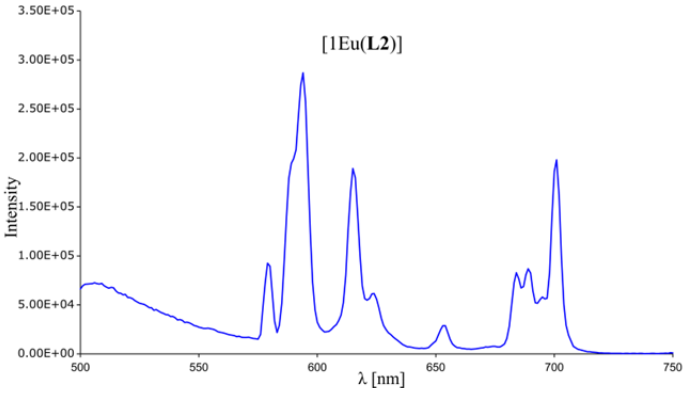

[1Eu(**L2**)]

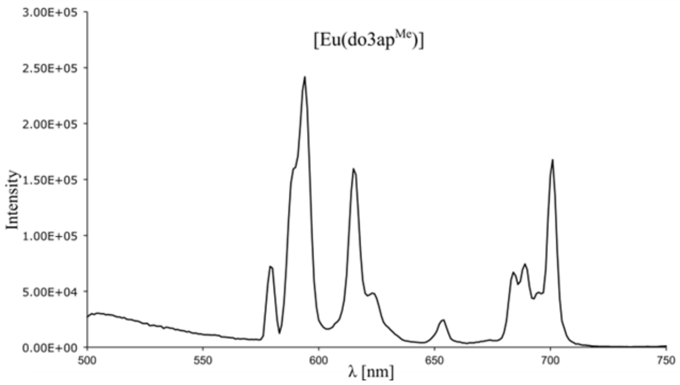

[Eu(do3ap^{Me})]

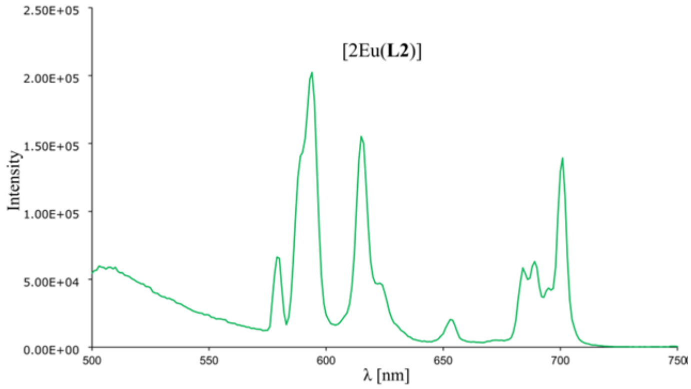

[2Eu(**L2**)]

^{89}Y NMR studies

Table S3. Calculated (DFT and empirical Equation 1) chemical shifts of diastereoisomers of the [Y(do3ap^{Me})] complexes with and without a single coordinated water molecule and their comparison with the experimental values.

Data	δ [ppm]			
	h-SA	*v*-SA	*v*-TSA	*h*-TSA
CN 8 (DFT)	196.1	197.1	192.6	194.2
CN 8 (empirical)[a]	227			
CN 9 (DFT)	118.5	117.6	102.2	103.4
CN 9 (empirical)[a]	119			
Experiment	–[b]	106.9	157.8	–[b]

[a]Determined from Equation (1) in the main text. [b]Diastereoisomer was not detected by NMR.

Figure S10. The ^{89}Y (29.4 MHz) NMR spectra of the [Y(do3ap^{Me})], [2Y(**L2**)] and [GdY(**L2**)] complexes at 5 °C showing signals of the *v*-TSA (major) and *v*-SA (minor) diastereoisomer.

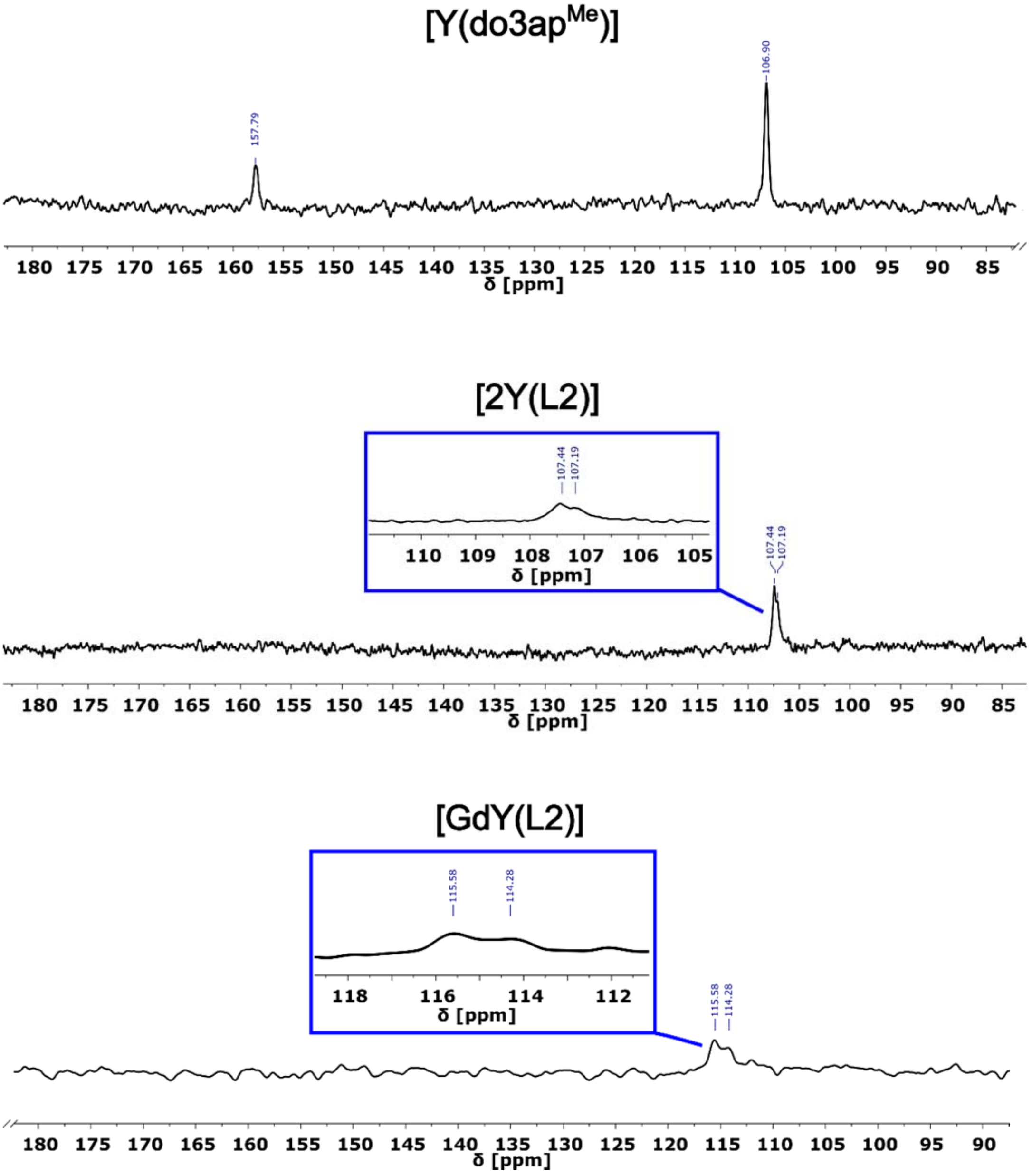

Determination of the rotational correlation time

Values of rotational correlation time, τ_R, at 5 °C, where dynamic processes are slow, were determined for the diamagnetic [2Y(**L2**)] complex and were considered to be the same for the [2Gd(**L2**)] complex. The time was determined using ^{13}C NMR R_1 relaxation rates and NOE factors of multiple CH_2 groups (Table S4) by an approximation used before for the complexes of DOTA and DO3AP^{Me}.[2,7,8] This approximation is based on a separation of a dipolar contribution to R_1 relaxation of carbon atoms bonded to hydrogen atoms, R_1^{DD}, by Equations S1 and S2. Here, NOE is the NOE enhancement factor, r_{CH} is the carbon-proton distance in $–CH_2–$ groups (value of 1.09 Å was obtained from DFT) and N(H) is the number of hydrogen atoms directly bound to the investigated carbon atom. The $\hbar$ = 1.0546·10^{-34} J s is the reduced Planck constant and γ_H = 2.675·10^{8} / γ_C = 6.728·10^{8} rad s^{-1} T^{-1} are the ^{1}H and ^{13}C magnetogyric ratios, respectively. The NOE factors were determined by comparing ^{13}C NMR signal intensities with and without NOE. These spectra were measured using a standard pulse-acquire sequence with a relaxation delay of d_1 = 3 s.

$$\tau_R = \frac{R_1^{DD} r_{CH}{}^6}{N(\mathrm{H})\,\hbar^2\,\gamma_H^2\,\gamma_C^2\,\hbar^2} \qquad \text{(S1)}$$

$$R_1^{DD} = R_1\,\frac{\mathrm{NOE}}{1.988} \qquad \text{(S2)}$$

The R_1 relaxation times of the macrocycle and arm $–CH_2–$ groups were determined by an inversion recovery measured at 20 values of variable delay exponentially distributed in the range of 0.06–1.5 s. The resulting intensities I were fitted to a three-parameter exponential (Equation S3) with A, B and R_1 as parameters.

$$I = A - Be^{-R_1 t} \qquad \text{(S3)}$$

Table S4. The NOE factors, ^{13}C NMR R_1 relaxation times and the corresponding rotational correlation times τ_R calculated from Equations S1 and S2 for an aqueous solution of the [2Y(**L2**)] complex at 5 °C.

δ_C [ppm]	NOE factor	R_1 [s^{-1}]	τ_R [ps]
55.7	1.54	6.71	121
55.3	1.56	6.89	126
55.2	1.36	6.51	104
54.9	1.54	6.72	121
54.7	1.54	6.90	124
54.5	1.37	6.82	110
65.3	1.59	6.35	118
65.5	1.55	6.36	115
65.7	1.53	6.34	113

Figure S11. Calculated geometries for different diastereoisomers of the [GdY(**L2**)] complex, where Gd–Y distances agree the best with the experimental value (5.7 Å). For simplicity, C–H hydrogen atoms are not shown. Colour codes: **hydrogen – white**, **carbon – brown**, **nitrogen – blue**, **oxygen – red**, **phosphorus – orange**, **yttrium – green** and **gadolinium - purple**. Visualized in Vesta[9].

(a) Geometry: Gd^{III} subunit – *v*-TSA, and Y^{III} subunit – *v*-SA. The DFT-predicted Y–Gd distance is 6.3 Å.

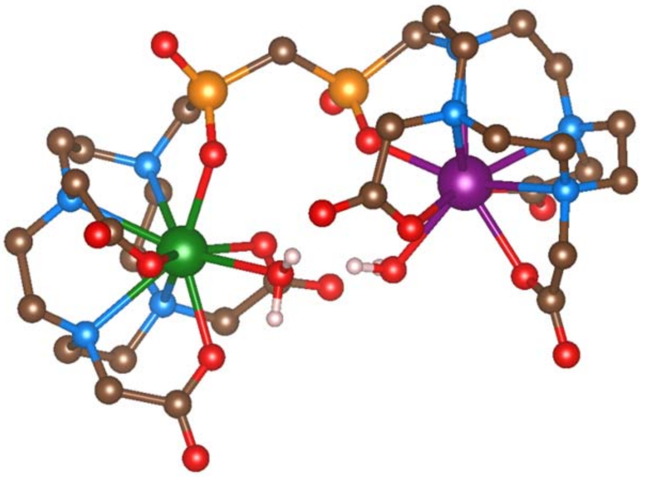

(b) Geometry: Gd^{III} subunit – *v*-SA, and Y^{III} subunit – *v*-TSA. The DFT-predicted Y–Gd distance is 6.2 Å.

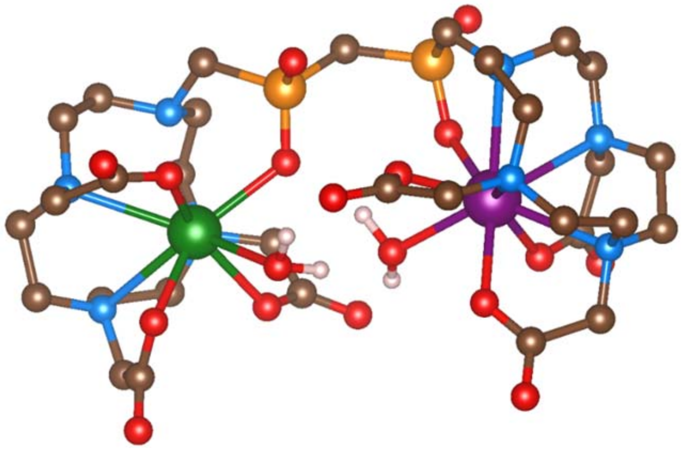

(c) Geometry: Gd^{III} subunit – *v*-TSA and Y^{III} subunit – *v*-TSA. The DFT-predicted Y–Gd distance is 7.2 Å.

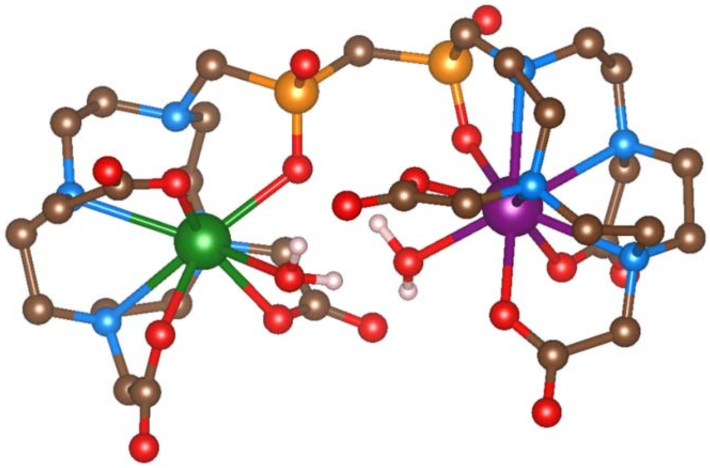

Table S5. The DFT-calculated energies of structures at minima on the potential energy surface, scanned by changing the P–O–P–C dihedral angle of the [GdY(**L2**)] complex for four diastereoisomers, compared with the corresponding Gd–Y distance. The lowest energy structures are in bold.

Diastereoisomer	*v*-SA(Gd-subunit) – *v*-SA(Y-subunit)			
Gd–Y distance	7.35	8.82	6.35	**5.90**
Energy [kJ mol^{-1}]	19.9	28.8	25.8	**0**
Geometry	*v*-TSA (Gd-subunit) – *v*-SA (Y-subunit)			
Gd–Y distance	6.51	**6.28**		
Energy [kJ mol^{-1}]	13.4	**0**		
Geometry	*v*-SA (Gd-subunit) – *v*-TSA (Y-subunit)			
Gd–Y distance	6.54	**6.20**		
Energy [kJ mol^{-1}]	14.6	**0**		
Geometry	*v*-TSA (Gd-subunit) – *v*-TSA (Y-subunit)			
Gd–Y distance	7.39	**7.19**		
Energy [kJ mol^{-1}]	64.0	**0**		

Solution dynamics

The 2D ^1H–^1H EXSY of the [Eu(**L1**)] complex (Figure S12) is fully analogous to the ^1H–^1H EXSY spectrum of the previously studied the [Eu(do3ap^{Me})] complex.[2] As expected, three types of dynamic processes were detected. The macrocycle inversion was confirmed by cross-peaks between signals of the "axial" and "equatorial" protons of the *v*-SA/*v*-TSA and *h*-SA/*h*-TSA pairs (**in blue**). The pendant arms re-orientation was confirmed by cross peaks between the "axial" protons of *v*-SA/*h*-TSA and *h*-SA/*v*-TSA pairs (**in red**). Finally, the "phosphinate rotation" was confirmed by cross peaks of "axial" protons of the *h*-TSA/*v*-TSA pair (**in green**). This process does not occur between the *h*-SA/*v*-SA diastereoisomers of [Eu(**L1**)]; analogously as was also found in complexes of other monophosphorus acid derivatives of H_4dota.[1,2]

Figure S12. The 2D ^1H–^1H EXSY (600 MHz) of [Eu(**L1**)] at 5 °C, pD ~7 and τ_m = 15 ms.

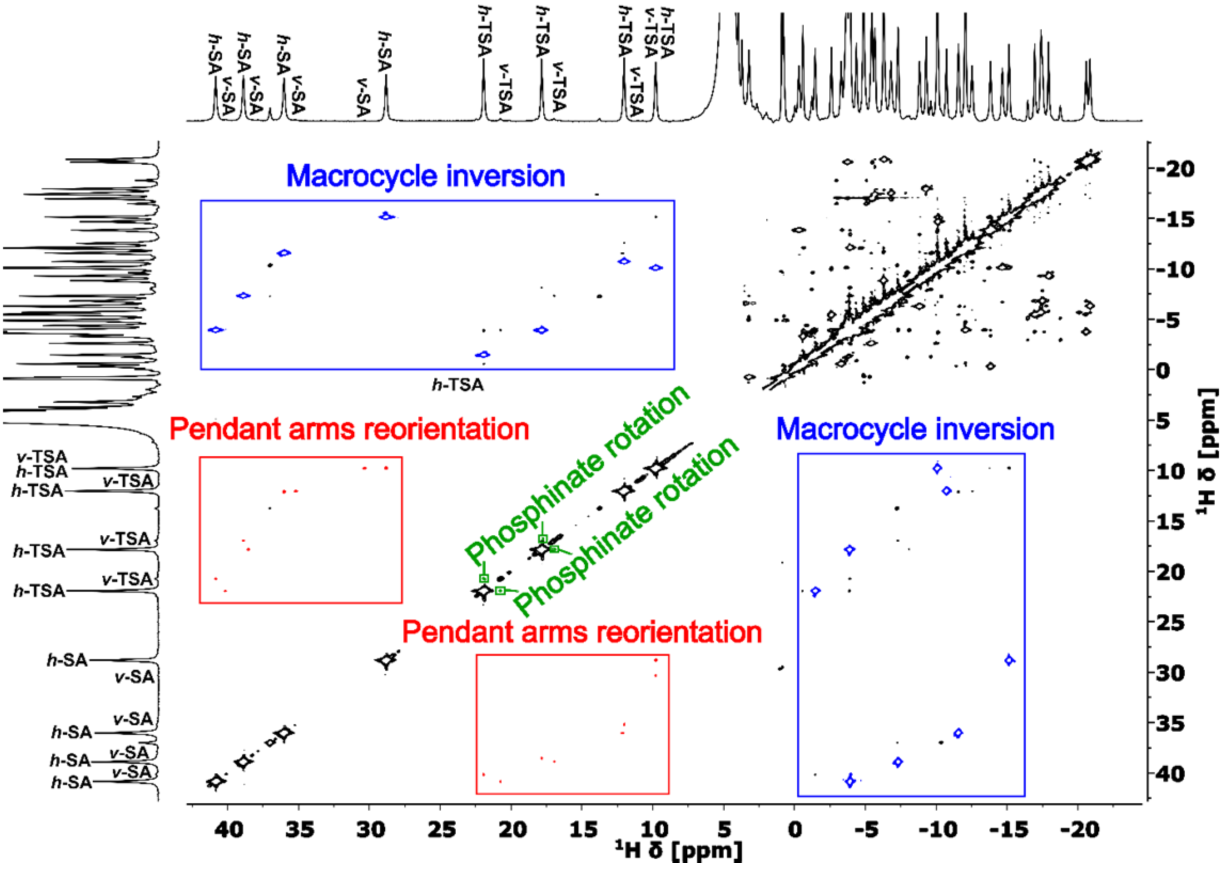

*Explanation for [2Eu(**L2**)] EXSY cross-peaks*

Figure S13 shows an example of chemical exchange affecting the macrocycle "equatorial" proton in the [2Ln(**L2**)] complexes. The only exchange process detected in the system is the macrocycle inversion with two possibilities for the selected proton: (*i*) It can be an *active* part of the inversion occurring on the same macrocycle subunit or (*ii*) it can be a *passive* process with the macrocycle inversion occurring on the other macrocyclic subunit. The process (*i*) exchanges the "equatorial" proton for the "axial" proton of a different diastereoisomer (in this example, an "equatorial" proton of the SA isomer changes to an "axial" proton of the TSA isomer). In process (*ii*), the "equatorial" proton does not exchange (this proton is on a subunit that does not change its geometry), but the geometry of the other subunit changes. The same applies to the phosphorus atom, which may be a part of the subunit where inversion occurs (active process) or part of the subunit that remains unchanged during the process (*passive* process). These types of processes were observed and distinguished in both 2D ^1H–^1H EXSY and 2D ^{31}P–^{31}P EXSY.

Figure S13. An example of chemical exchange by macrocyclic inversion in the [2Ln(**L2**)] complexes leading to two cross peaks for each signal: (*i*) an *active* exchange involving the subunit where the studied hydrogen/phosphorus atom is located and (*ii*) a *passive* exchange where exchange occurs on the subunit not containing the studied hydrogen/phosphorus atom.

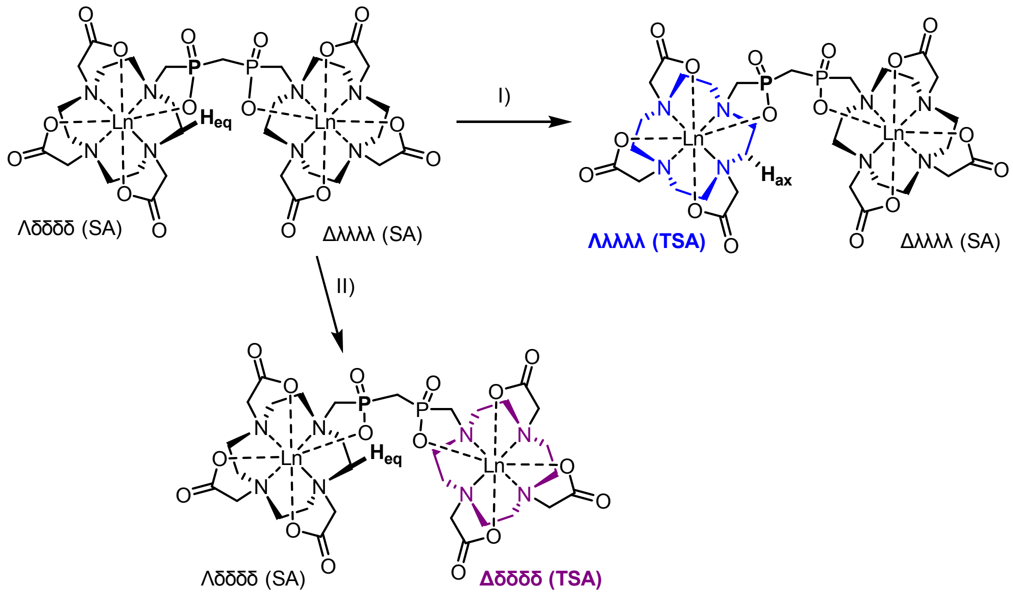

Figure S14. The 2D ^{31}P–^{31}P (243 MHz) EXSY of the [2Eu(**L2**)] complex at 5 °C, pD ~7 and τ_m = 15 ms.

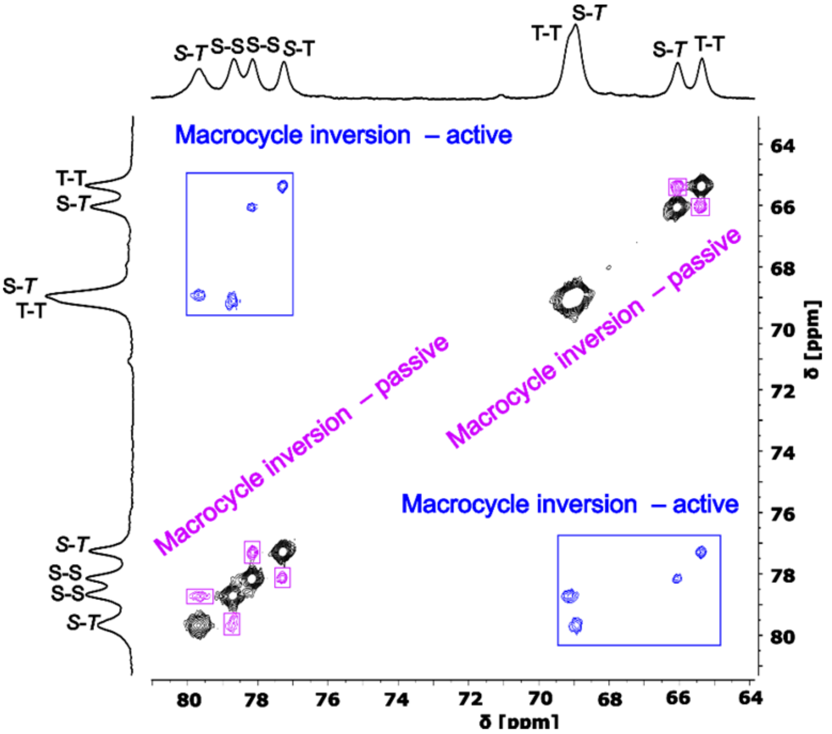

Signals in Figure S13 assigned to various types of diastereoisomers are labelled as follows: T-T – diastereoisomers consisting of two TSA subunits (*sm*/*df*-TSA), S-S – diastereoisomers consisting of two SA subunits (*sm*/*df*-SA) and T-S – diastereoisomers consisting of a mixture of one TSA and one SA subunit (*cyc*/*arm*-TSA–SA). Signals of the corresponding subunit are in italics.

Figure S15. Variable-temperature ^{31}P (243 MHz) NMR spectra of the [2Eu(**L2**)] complex measured at pD ~7.

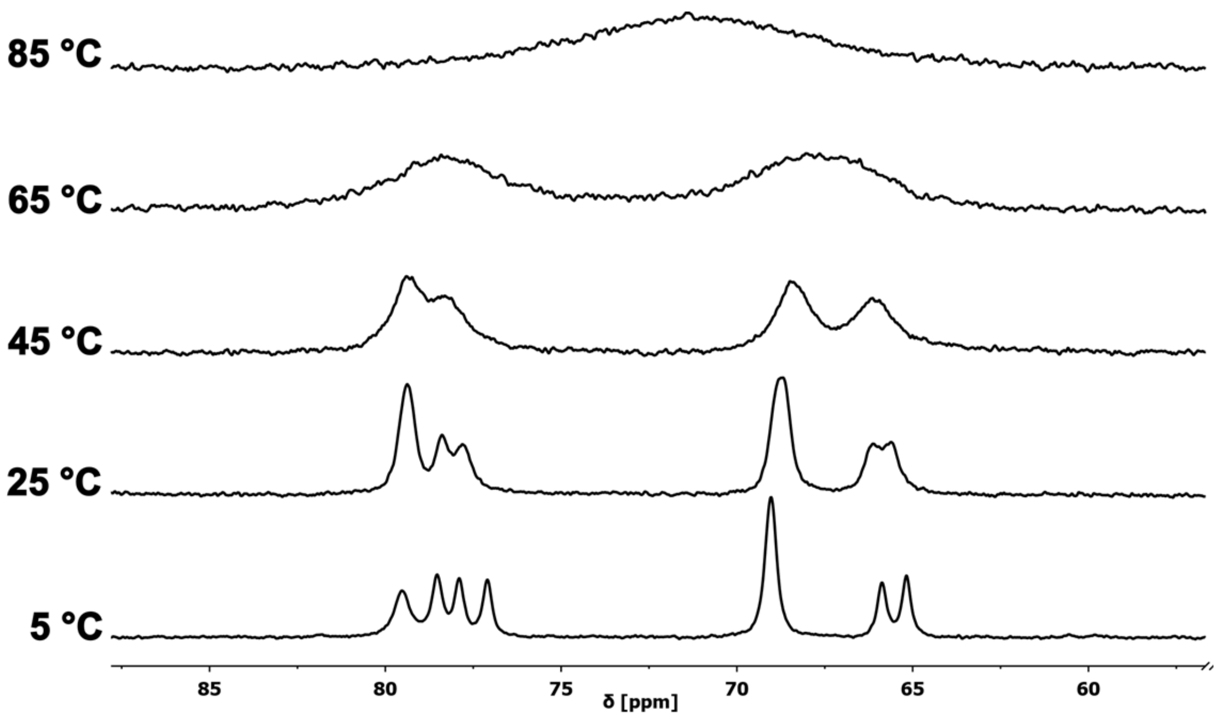

Figure S16. The 2D ^{1}H–^{1}H EXSY (600 MHz) of the [2Ce(**L2**)] complex at 5 °C, pD ~7 and τ_m = 15 ms. Cross-peaks corresponding to the *sm*-TSA/*df*-TSA isomer exchange are shown **in red**.

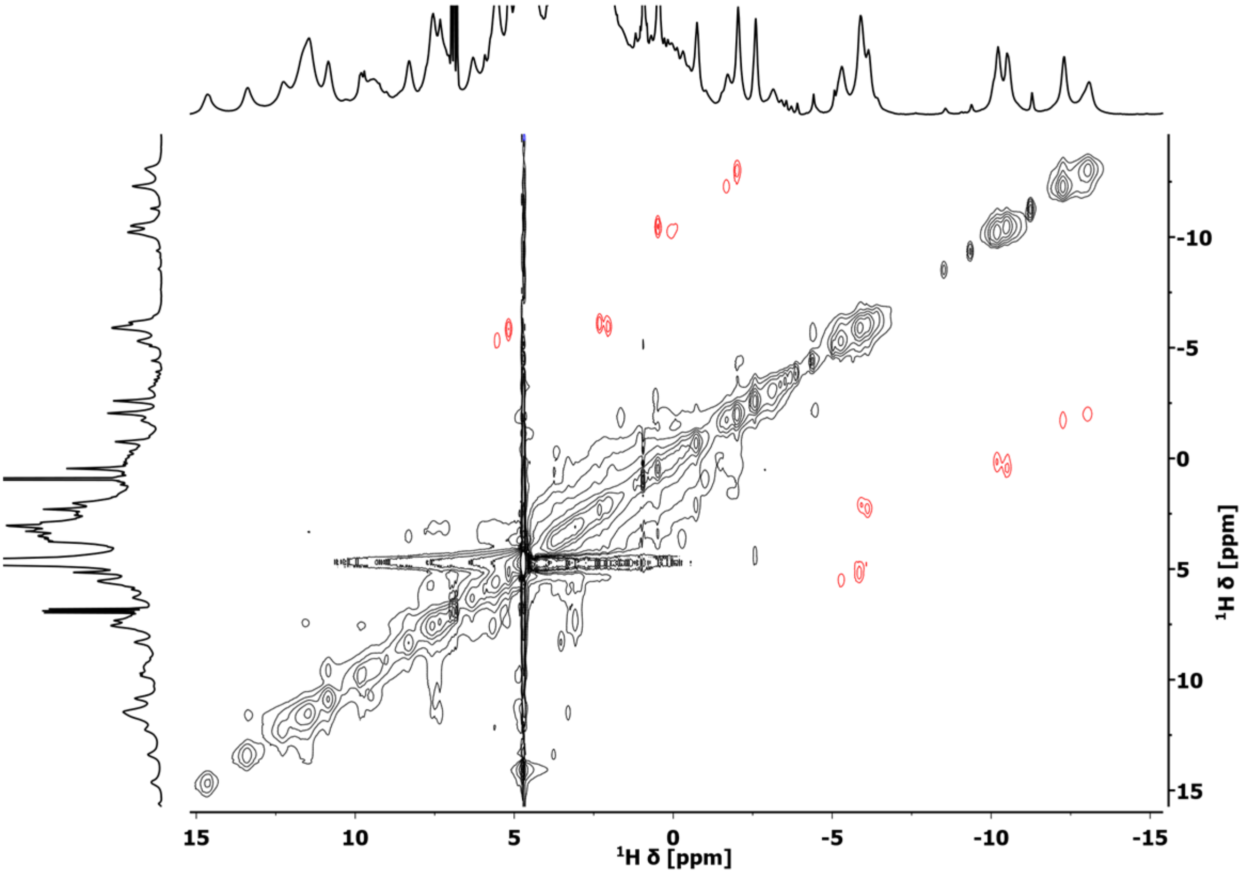

Relaxivity of the Gd^{III} complexes

Determination of the Gd^{III} concentration by Evans method

Samples of the [Gd(**L1**)], [1Gd(**L2**)] and [2Gd(**L2**)] complexes for relaxivity determination were prepared by dissolving the solid complexes in water (0.5 ml) to get an approximate concentration of 1 mM. The solution pH was adjusted to the desired value by 0.5% aq. NH_3 or 0.5% aq. HCl. The final concentration of Gd^{III} ion in the samples was determined from the difference in chemical shift of the ^{1}H signal of the methyl group of *t*BuOH added to the sample, and *t*BuOH dissolved in D_2O in a coaxial insert tube, Δδ. This difference results from the bulk magnetic susceptibility of the sample[10] which depends on the concentration of paramagnetic species according to Equation S4 (ref.[11]) where μ_{eff} = 7.94 BM is the effective magnetic moment of Gd^{III} taken from literature.[12]

$$c_{\mathrm{Ln}} = \frac{2{,}84^2 \cdot T \cdot \Delta\delta}{4\pi \cdot \left(\frac{1}{3} - \alpha\right) \cdot \mu_{\mathrm{eff}}^2} \qquad \text{(S4)}$$

Figure S17. Relaxivities (per Gd ion) of the Gd^{III} complexes of **L1** and **L2** (25 °C, 0.94 T) at different pH values.

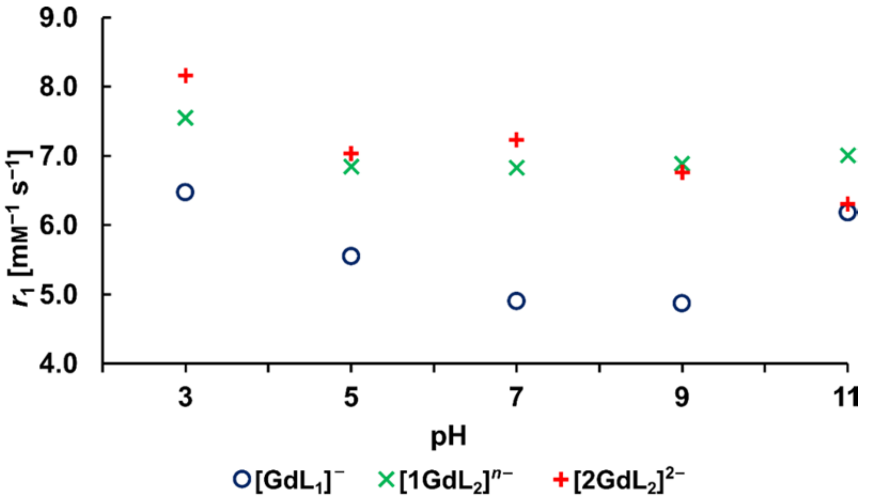

The pH was adjusted by aq. NH_3 (0.5%) or aq. HCl (0.5%). The difference in relaxivity of [Gd(**L1**)] at pH 5 and 7 (no protonation of the complex is expected in this pH range) could not be explained, but we propose a change in second-sphere hydration as the most likely explanation.

Experimental data

Notes on the synthesis of ***L1***

Ligand **L1** was synthesised by a modified literature procedure. Unlike in the literature procedure,[13] ligand **L1** was precipitated by dissolving the oily crude product in a minimal amount of water (approx. 10 ml per 0.5 g of **L1**) and adding CH_3OH (approx. 30 ml), and followed by acetone (approx. 30 ml) until the precipitation of a white powder with **L1**·$3H_2O$ composition. Thus, the composition of **L1** differs from that in the literature. Elem. analysis: found (required for **L1**·$3H_2O$): C 34.02 (34.54), H 6.83 (6.88), N 9.72 (10.07), P 9.7 (11.5). The solution NMR and MS data are consistent with the published data.[13]

Synthesis of diisopropyl methylene-bis(H-phosphinate) ***1***.

Under argon atmosphere, a solution of bis(dichlorophosphino)methane (19.0 g, 87 mmol, 1 equiv.) in anhydrous THF (cca 50 ml) was cooled to 0 °C in an ice/water bath. Next, a solution of anhydrous pyridine (14 ml, 0.19 mol, 2 equiv.) and anhydrous *i*PrOH (29 ml, 0.38 mol, 4.4 equiv.) in anhydrous THF (70 ml) was added dropwise into an intensively stirred solution of the chlorophosphine. A precipitation of pyridinium hydrochloride started immediately. This suspension was stirred at room temperature for 24 h. The precipitate was filtered off on a fine glass frit and washed with THF (3×15 ml). Volatiles were evaporated from the filtrate *in vacuo*. The residue was purified by column chromatography (SiO_2, 300 g, *i*PrOH; TLC: R_f ~0.5). After the evaporation of volatiles from the combined pure product-containing fractions, the product was isolated as a colourless liquid (16.36 g, 82%) and stored in a refrigerator. The product was obtained as a mixture of two diastereoisomers in approximately 1:1 ratio (^{31}P NMR).

^{1}H NMR (300 MHz, $CDCl_3$) δ 0.82 – 1.48 (m, 12H, OCH(C$\underline{H}_3$)$_2$), 2 × 2.32 (t, $^2J_{HP}$ 17.6 Hz, 2H, PC$\underline{H}_2$P), 4.38 – 4.58 (m, 2H, OC$\underline{H}$(CH$_3$)$_2$), 2 × 7.15 (dt, $^1J_{HP}$ 579 Hz, $^3J_{HH}$ 2.2 Hz, 2H, P–$\underline{H}$). Two sets of overlapping signals of the two diastereoisomers were present in the spectra, with chemical shift differences <0.01 ppm.

^{13}C{^{1}H} NMR (101 MHz, $CDCl_3$) δ 23.1 – 23.3 and 23.8 – 24.0 (m, OCH($\underline{C}$H$_3$)$_2$), 30.78 (t, $^1J_{CP}$ 83.3 Hz, P$\underline{C}$H$_2$P) and 30.82 (t, $^1J_{CP}$ 83.7 Hz, P$\underline{C}$H$_2$P), 72.37 – 72.90 (m, O$\underline{C}$H(CH$_3$)$_2$).

^{31}P{^{1}H} NMR (121 MHz, $CDCl_3$) δ 23.0 and 23.2.

TLC (*i*PrOH): R_f 0.55.

Characterization of complexes

Table S6. Acquisition times t_A and relaxation delays d_1 used to acquire the paramagnetic NMR spectra.

^{1}H NMR		
Complexes	t_A [ms]	d_1 [ms]
[2Ce(**L2**)], [2Yb(**L2**)], [Ce(**L1**)], [Yb(**L1**)]	500	200
[2Pr(**L2**)], [2Nd(**L2**)], [Pr(**L1**)] , [Nd(**L1**)]	200	200
[2Tb(**L2**)], [2Dy(**L2**)], [2Ho(**L2**)] [a]	30	50
[2Eu(**L2**)], [2Sm(**L2**)], [Eu(**L1**)], [Sm(**L1**)]	1000	300
[2Er(**L2**)], [2Tm(**L2**)], [Tb(**L1**)], [Dy(**L1**)], [Ho(**L1**)], [Er(**L1**)], [Tm(**L1**)]	50	50
^{31}P NMR		
Complexes	t_A [ms]	d_1 [ms]
[2Ce(**L2**)], [2Pr(**L2**)], [2Nd(**L2**)], [Ce(**L1**)], [Pr(**L1**)], [Nd(**L1**)], [Er(**L1**)], [Yb(**L1**)]	100	200
[2Eu(**L2**)], [2Sm(**L2**)], [Eu(**L1**)], [Sm(**L1**)]	300	200
[2Tb(**L2**)], [2Dy(**L2**)], [2Ho(**L2**)], [2Er(**L2**)], [2Tm(**L2**)], [Tb(**L1**)], [Dy(**L1**)], [Ho(**L1**)], [Er(**L1**)], [Tm(**L1**)]	50	50
[2Yb(**L2**)]	80	100

[a]The ^{1}H NMR signals were very broad and could not be distinguished from the baseline distortions.

Table S7. The ^{1}H NMR chemical shifts of the "axial" protons of the paramagnetic [Ln(**L1**)] complexes at 5 °C, along with their assignment to the TSA/SA diastereoisomers. For a more specific assignment of the signals to the diastereoisomers in some complexes, see Figure S2.

Complex	δ_H [ppm]	
	v-TSA/ TSA′	*v*-SA
[Ce(**L1**)]	−13.2, −10.6, −6.5, −6.0	–[a]
[Pr(**L1**)]	−37.1, −31.6, −20.7, −19.2	−53.7, −50.3, −48.0, −36.0
[Nd(**L1**)]	−14.7, −11.6, −8.2, −6.1	−27.8, −27.4, −25.1, −21.3
[Eu(**L1**)]	9.8, 12.0, 17.8, 21.9	28.8, 36.0, 38.9, 40.9
[Tb(**L1**)]	−360, −308, −202, −199	−487, −459, −438, −355
[Dy(**L1**)]	−526, −464,−403[b]	−598, −541, −532, −392
[Ho(**L1**)]	−306, −293, −270, −227	−274, −231, −182, −150
[Er(**L1**)]	173, 196, 248, 258	131, 180 (2×), 200
[Tm(**L1**)]	527, 542, 604, 665	349, 428, 456, 475
[Yb(**L1**)]	56, 74, 99, 124	119, 153, 160, 173

[a]SA diastereoisomer is not present. [b]A signal of the diastereoisomer could not be distinguished due to an overlap.

Table S8. The ^{31}P NMR chemical shifts of the [Ln(**L1**)] complexes at 5 °C, and their assignment to the TSA/SA diastereoisomers. For a more specific assignment of the signals to diastereoisomers in some complexes, see Figure S3.

Complex	δ_P [ppm]			
	NCH$_2$P		P–H	
	v-TSA/ TSA′	*v*-SA	*v*-TSA/TSA′	*v*-SA
[La(**L1**)]	35.5 (bs)	–[a]	16.9 (dtd, $^1J_{PH}$ = 536, $^2J_{PH}$ = 17.8, $^3J_{PP}$ = 4 Hz)	–[a]
[Ce(**L1**)]	25.4	–[a]	18.7 (d, $^1J_{PH}$ = 536 Hz)	–[a]
[Pr(**L1**)]	28.9	30.7	21.5 (d, $^1J_{PH}$ = 535 Hz)	20.8 (d, $^1J_{PH}$ = 535 Hz)
[Nd(**L1**)]	−1.3	1.2	18.1 (d, $^1J_{PH}$ = 535 Hz)	17.5 (d, $^1J_{PH}$ = 534 Hz)
[Sm(**L1**)]	43.7	49.3	17.1 (d, $^1J_{PH}$ = 535 Hz)	17.3 (d, $^1J_{PH}$ = 535 Hz)
[Eu(**L1**)]	67.5	78.5	15.9 (d, $^1J_{PH}$ = 535 Hz)	16.4 (d, $^1J_{PH}$ = 537 Hz)
[Tb(**L1**)]	381	483	59 (d, $^1J_{PH}$ = 509 Hz)	34 (d, $^1J_{PH}$ = 496 Hz)
[Dy(**L1**)]	592	486	78 (d, $^1J_{PH}$ = 494 Hz)	56 (d, $^1J_{PH}$ = 481 Hz)
[Ho(**L1**)]	291	221	50 (d, $^1J_{PH}$ = 514 Hz)	30 (d, $^1J_{PH}$ = 504 Hz)
[Er(**L1**)]	−130	−68	1 (d, $^1J_{PH}$ = 540 Hz)	11 (d, $^1J_{PH}$ = 535 Hz)
[Tm(**L1**)]	−406	−267	0 (d, $^1J_{PH}$ = 548 Hz)	−36 (d, $^1J_{PH}$ = 549 Hz)
[Yb(**L1**)]	−54	−90	4 (d, $^1J_{PH}$ = 533 Hz)	8.9 (d, $^1J_{PH}$ = 529 Hz)
[Lu(**L1**)]	37.8 (bs)	39.8 (bs)	17.2 (dt, $^1J_{PH}$ = 537, $^2J_{PH}$ = 16.8)	17.0 (dt, $^1J_{PH}$ = 537)[b]

[a]SA diastereoisomer is not present.

Table S9. The ^{1}H NMR chemical shifts of the “axial” protons of some paramagnetic [2Ln(**L2**)] complexes at 5 °C, along with their assignment to the TSA/SA diastereoisomers. For the Gd^{III}–Tm^{III} complexes, signals could not be reliably distinguished from baseline distortions. For a more specific assignment of the signals, see Figure S4.

Complex	δ_H [ppm]	
	TSA/ TSA′	SA
[2Ce(**L2**)]	−13.0, −12.2, −10.5, −10.1, −6.1, −5.9 (2×), −5.3	–[a]
[2Pr(**L2**)]	−36.6, −36.2, −35.3, −34.5, −31.3, −30.5, −29.6, −20.3, −19.6, −18.9, −18.2, −17.8[b]	−53.2, −52.6 −49.4, −48.6, −48.0, −47.0, −35.3 (2×)[b]
[2Nd(**L2**)]	−14.7, −14.5, −13.9, −13.2, −11.6, −11.5, −11.3, −10.7, −8.0, −7.7, −7.4, −7.0, −5.9, −5.7, −5.2, −5.0	−27.5, −27.2 (4×), −26.5, −25.5, −25.0, −24.5, −21.0, −20.7, −20.5
[2Eu(**L2**)]	8.6, 9.1, 9.5, 9.6, 10.7, 11.1, 11.7, 11.8, 16.6, 17.1, 17.7, 17.8, 20.0, 20.7, 21.7 (2×)	27.9, 28.3, 28.4, 28.8, 35.2, 35.8, 36.2, 36.5, 37.8, 38.2, 38.8, 39.0, 40.0, 40.5 (3×)
[2Yb(**L2**)]	51.4, 52.1, 52.8, 56.3, 68.6, 70.6, 71.2, 72.4, 96.5 (2×), 98.5 (2×), 120.2, 120.8, 121.1, 125.6	111.0, 113.3, 116.9, 146.7, 149.0, 151.9, 152.9, 153.6, 154.6, 158.0, 160.7, 165.0, 169.3, 170.6, 174.2[b]

[a]SA diastereoisomer is not present. [b]Some signals of minor diastereoisomers could not be distinguished.

Table S10. The ^{31}P NMR chemical shifts of the [2Ln(**L2**)] complexes at 5 °C, and their assignment to the TSA/SA diastereoisomers. For a more specific assignment of the signals to diastereoisomers in some complexes, see Figure S5.

<table>
<tr><th rowspan="2">Complex</th><th colspan="3">δ_P [ppm]</th></tr>
<tr><th colspan="2">TSA/ TSA′</th><th>SA</th></tr>
<tr><td>[2La(L2)]</td><td colspan="2">34.5, 35.3</td><td>–</td></tr>
<tr><td>[2Ce(L2)]</td><td colspan="2">25, 26</td><td>–</td></tr>
<tr><td>[2Pr(L2)]</td><td>25.2, 35.4</td><td colspan="2">27.5, 31.6, 30.0, 34.2, 37.2[a]</td></tr>
<tr><td>[2Nd(L2)]</td><td>–3.0, –0.9</td><td colspan="2">–1.8, –1.4, 0.3, 0.5, 1.6[a]</td></tr>
<tr><td>[2Sm(L2)]</td><td colspan="2">43.5, 43.8, 44.1[b]</td><td>49.3, 49.6, 59.8[b]</td></tr>
<tr><td>[2Eu(L2)]</td><td colspan="2">65.2, 65.9, 69.0 (2×)</td><td>77.1, 77.9, 78.5, 79.6</td></tr>
<tr><td>[2Tb(L2)]</td><td colspan="2">350–424</td><td>467–534</td></tr>
<tr><td>[2Dy(L2)]</td><td colspan="2">424–500</td><td>551–627</td></tr>
<tr><td>[2Ho(L2)]</td><td colspan="2">204–270</td><td>291–353</td></tr>
<tr><td>[2Er(L2)]</td><td colspan="2">–184, –143, –131, –109</td><td>–90, –85, –76, –68</td></tr>
<tr><td>[2Tm(L2)]</td><td colspan="2">–449, –400, –389, –385</td><td>–311(2×), –267, –250</td></tr>
<tr><td>[2Yb(L2)]</td><td colspan="2">–69.0, –67.9 (2×), –53.9</td><td>–105.9, –105.3, –97.9, –91.7</td></tr>
<tr><td>[2Lu(L2)]</td><td colspan="2">36.8, 37.0, 37.6, 38.1</td><td>38.6, 38.8, 39.4, 39.9</td></tr>
</table>

[a]Due to the low LIS, signals could not be assigned to the TSA/SA subunit. [b]Due to a low abundance, signals of one diastereoisomer could not be distinguished.

Mass spectra

Table S11. Values of *m/z* corresponding to the most intensive signals in mass spectra of the [Ln(**L1**)] complexes, their assignment and theoretical composition of the corresponding species. The **L1** denotes the fully deprotonated ligand. The Li^+ counter cations are present due to the precipitation of excess lanthanide(III) as corresponding hydroxides by LiOH addition during the purification procedure.

Complex	*m/z* (ESI+)			*m/z* (ESI−)		
[Ln(**L1**)]	Experimental	Theoretical	Composition	Experimental	Theoretical	Composition
[La(**L1**)]	657.14	657.1	[^{139}La(**L1**)+3Li]$^+$	637.13	637.0	[^{139}La(**L1**)−H]$^-$
[Ce(**L1**)]	640.25	640.0	[^{140}Ce(**L1**)+H]$^+$	638.14	638.0	[^{140}Ce(**L1**)−H]$^-$
[Pr(**L1**)]	659.04	659.1	[^{141}Pr(**L1**)+3Li]$^+$	639.16	639.0	[^{141}Pr(**L1**)−H]−
[Nd(**L1**)]	659.99	660.1	[^{142}Nd(**L1**)+3Li]$^+$	640.07	642.0	[^{142}Nd(**L1**)−H]$^-$
[Nd(**L1**)]	662.56	662.1	[^{144}Nd(**L1**)+3Li]$^+$	642.09	644.0	[^{144}Nd(**L1**)−H]$^-$
[Sm(**L1**)]	664.30	664.1	[^{152}Nd(**L1**)+2Li]$^+$	650.04	650.0	[^{152}Nd(**L1**)−H]$^-$
[Sm(**L1**)]	665.9	666.1	[^{154}Nd(**L1**)+2Li]$^+$	652.23	652.1	[^{154}Nd(**L1**)−H]$^-$
[Eu(**L1**)]	657.59	657.1	[^{151}Eu(**L1**)+Li]$^+$	649.08	649.0	[^{151}Eu(**L1**)−H]$^-$
[Eu(**L1**)]	659.04	659.1	[^{153}Eu(**L1**)+Li]$^+$	651.24	651.0	[^{153}Eu(**L1**)−H]$^-$
[Gd(**L1**)]	656.14	656.1	[^{156}Gd(**L1**)+H]$^+$	654.18	654.1	[^{156}Gd(**L1**)−H]$^-$
[Gd(**L1**)]	658.13	658.1	[^{158}Gd(**L1**)+H]$^+$	656.13	656.1	[^{158}Gd(**L1**)−H]$^-$
[Gd(**L1**)]	659.84	660.1	[^{160}Gd(**L1**)+H]$^+$	658.18	658.1	[^{160}Gd(**L1**)−H]$^-$
[Tb(**L1**)]	677.08	677.1	[^{159}Tb(**L1**)+3Li]$^+$	657.12	657.1	[^{159}Tb(**L1**)−H]$^-$
[Dy(**L1**)]	678.79	679.1	[^{161}Dy(**L1**)+3Li]$^+$	659.09	659.1	[^{161}Dy(**L1**)−H]$^-$
[Dy(**L1**)]	680.17	680.1	[^{162}Dy(**L1**)+3Li]$^+$	660.07	660.1	[^{162}Dy(**L1**)−H]$^-$
[Dy(**L1**)]	681.11	681.1	[^{163}Dy(**L1**)+3Li]$^+$	661.18	661.1	[^{163}Dy(**L1**)−H]$^-$
[Dy(**L1**)]	682.03	682.1	[^{164}Dy(**L1**)+3Li]$^+$	662.17	662.1	[^{164}Dy(**L1**)−H]$^-$
[Ho(**L1**)]	683.03	683.1	[^{165}Ho(**L1**)+3Li]$^+$	663.15	663.1	[^{165}Ho(**L1**)−H]$^-$
[Er(**L1**)]	684.46	684.1	[^{166}Er(**L1**)+3Li]$^+$	664.18	664.1	[^{166}Er(**L1**)−H]$^-$
[Er(**L1**)]	685.27	685.1	[^{167}Er(**L1**)+3Li]$^+$	665.14	665.1	[^{167}Er(**L1**)−H]$^-$
[Er(**L1**)]	686.13	686.1	[^{168}Er(**L1**)+3Li]$^+$	666.17	666.1	[^{168}Er(**L1**)−H]$^-$
[Tm(**L1**)]	687.18	687.1	[^{169}Tm(**L1**)+3Li]$^+$	667.10	667.1	[^{169}Tm(**L1**)−H]$^-$
[Yb(**L1**)]	689.91	690.1	[^{172}Yb(**L1**)+3Li]$^+$	670.10	670.1	[^{172}Yb(**L1**)−H]$^-$
[Yb(**L1**)]	691.16	691.1	[^{173}Yb(**L1**)+3Li]$^+$	671.21	671.1	[^{173}Yb(**L1**)−H]$^-$
[Yb(**L1**)]	692.15	692.1	[^{174}Yb(**L1**)+3Li]$^+$	672.08	672.1	[^{174}Yb(**L1**)−H]$^-$
[Lu(**L1**)]	692.88	693.1	[^{175}Lu(**L1**)+3Li]$^+$	673.20	673.1	[^{175}Lu(**L1**)−H]$^-$
[Y(**L1**)]	606.88	607.1	[^{89}Y(**L1**)+3Li]$^+$	587.10	587.0	[^{89}Y(**L1**)−H]$^-$

Table S12. Values of *m/z* corresponding to the most intensive signals in mass spectra of the [2Ln(**L2**)] complexes, their assignment and theoretical composition of the corresponding species. The **L2** denotes the fully deprotonated ligand. Only single-charged species are included.

Complex	*m/z* (ESI+)		*m/z* (ESI−)	
[2Ln(**L2**)]	Exper. (theor.)	Composition	Exper. (theor.)	Composition
[2La(**L2**)]	1132.82 (1133.12)	$[^{139}La^{139}La($**L2**$)+3H]^+$	1131.11 (1131.10)	$[^{139}La^{139}La\ ($**L2**$)+H]^-$
[2Ce(**L2**)]	1135.01 (1135.12)	$[^{140}Ce^{141}Ce($**L2**$)+3H]^+$	1133.39 (1133.10)	$[^{140}Ce^{141}Ce($**L2**$)+H]^-$
[2Pr(**L2**)]	1137.33 (1137.1)	$[^{141}Pr^{141}Pr($**L2**$)+3H]^+$	1135.17 (1135.1)	$[^{141}Pr^{141}Pr\ ($**L2**$)+H]^-$
[2Nd(**L2**)]	1143.39 (1143.13)	$[^{144}Nd^{144}Nd($**L2**$)+3H]^+$ /$[^{142}Nd^{146}Nd($**L2**$)+3H]^+$	1141.60 (1411.11)	$[^{144}Nd^{144}Nd($**L2**$)+H]^-$ / $[^{142}Nd^{146}Nd($**L2**$)+H]^-$
[2Nd(**L2**)]	1140.81 (1141.12)	$[^{142}Nd^{144}Nd($**L2**$)+3H]^+$/ $[^{143}Nd^{143}Nd($**L2**$)+3H]^+$	1139.31 (1139.11)	$[^{142}Nd^{144}Nd($**L2**$)+H]^-$ / $[^{143}Nd^{143}Nd($**L2**$)+H]^-$
[2Nd(**L2**)]	1142.43 (1142.13)	$[^{143}Nd^{144}Nd($**L2**$)+3H]^+$	1140.58 (1140.11)	$[^{143}Nd^{144}Nd($**L2**$)+H]^-$
[2Nd(**L2**)]	1144.28 (1144.13)	$[^{143}Nd^{146}Nd($**L2**$)+3H]^+$	1142.31 (1142.11)	$[^{143}Nd^{146}Nd($**L2**$)+H]^-$
[2Nd(**L2**)]	1145.18 (1145.13)	$[^{144}Nd^{146}Nd($**L2**$)+3H]^{3+}$	1142.90 (1143.11)	$[^{144}Nd^{146}Nd($**L2**$)+H]^-$
[2Nd(**L2**)]	1146.74 (1147.13)	$[^{146}Nd^{146}Nd($**L2**$)+3H]^+$	1445.54 (1445.12)	$[^{146}Nd^{146}Nd($**L2**$)+H]^-$
[2Sm(**L2**)]	1155.68 (1156.14)	$[^{154}Sm^{147}Sm($**L2**$)+3H]^+$/ $[^{152}Sm^{149}Sm($**L2**$)+3H]^+$	1154.10 (1154.13)	$[^{154}Sm^{147}Sm($**L2**$)+H]^-$/ $[^{152}Sm^{149}Sm($**L2**$)+H]^-$
[2Sm(**L2**)]	1157.12 (1157.14)	$[^{154}Sm^{148}Sm($**L2**$)+3H]^+$	1154.89 (1155.13)	$[^{154}Sm^{148}Sm($**L2**$)+H]^-$
[2Sm(**L2**)]	1159.20 (1159.14)	$[^{152}Sm^{152}Sm($**L2**$)+3H]^+$	1157.05 (1157.13)	$[^{152}Sm^{152}Sm($**L2**$)+H]^-$
[2Sm(**L2**)]	1161.41 (1161.15)	$[^{154}Sm^{152}Sm($**L2**$)+3H]^+$	1158.60 (1159.13)	$[^{154}Sm^{152}Sm($**L2**$)+H]^-$
[2Sm(**L2**)]	1158.25 (1158.14)	$[^{154}Sm^{149}Sm($**L2**$)+3H]^+$	1155.88 (1156.13)	$[^{154}Sm^{149}Sm($**L2**$)+H]^-$
[2Sm(**L2**)]	1154.10 (1154.14)	$[^{152}Sm^{147}Sm($**L2**$)+3H]^+$	1152.37 (1152.12)	$[^{152}Sm^{147}Sm($**L2**$)+H]^-$

[2Eu(**L2**)]	1159.07 (1159.15)	$[^{151}Eu^{153}Eu(\mathbf{L2})+3H]^+$	1157.32 (1157.13)	$[^{151}Eu^{153}Eu(\mathbf{L2})+H]^-$
[2Eu(**L2**)]	1161.64 (1161.15)	$[^{153}Eu^{153}Eu(\mathbf{L2})+3H]^+$	1159.10 (1159.13)	$[^{153}Eu^{153}Eu(\mathbf{L2})+H]^-$
[2Eu(**L2**)]	1157.35 (1157.15)	$[^{151}Eu^{151}Eu(\mathbf{L2})+3H]^+$	1155.31 (1155.13)	$[^{151}Eu^{151}Eu(\mathbf{L2})+H]^-$
[2Gd(**L2**)]	1171.08 (1171.15)	$[^{156}Gd^{160}Gd(\mathbf{L2})+3H]^+$/ $[^{158}Gd^{158}Gd(\mathbf{L2})+3H]^+$	1169.25 (1169.14)	$[^{156}Gd^{160}Gd(\mathbf{L2})+H]^-$/ $[^{158}Gd^{158}Gd(\mathbf{L2})+H]^-$
[2Gd(**L2**)]	1170.36 (1170.16)	$[^{157}Gd^{158}Gd(\mathbf{L2})+3H]^+$/ $[^{155}Gd^{160}Gd(\mathbf{L2})+3H]^+$	1167.89 (1168.14)	$[^{157}Gd^{158}Gd(\mathbf{L2})+H]^-$/ $[^{155}Gd^{160}Gd(\mathbf{L2})+H]^-$
[2Gd(**L2**)]	1168.86 (1168.15)	$[^{157}Gd^{157}Gd(\mathbf{L2})+3H]^+$	1167.12 (1167.14)	$[^{157}Gd^{157}Gd\ (\mathbf{L2})+H]^-$
[2Gd(**L2**)]	1168.00 (1168.14)	$[^{156}Gd^{157}Gd\ (\mathbf{L2})+3H]^+$	1166.15 (1166.14)	$[^{156}Gd^{157}Gd\ (\mathbf{L2})+H]^-$
[2Gd(**L2**)]	1173.10 (1173.14)	$[^{158}Gd^{160}Gd\ (\mathbf{L2})+3H]^+$	1170.86 (1171.14)	$[^{158}Gd^{160}Gd\ (\mathbf{L2})+H]^-$
[2Gd(**L2**)]	1172.00 (1172.14)	$[^{157}Gd^{160}Gd\ (\mathbf{L2})+3H]^+$	1169.96 (1170.14)	$[^{157}Gd^{160}Gd\ (\mathbf{L2})+H]^-$
[2Tb(**L2**)]	1173.49 (1173.16)	$[^{159}Tb^{159}Tb(\mathbf{L2})+3H]^+$	1171.11 (1171.14)	$[^{159}Tb^{159}Tb(\mathbf{L2})+H]^-$
[2Dy(**L2**)]	1180.85 (1181.16)	$[^{164}Dy^{162}Dy(\mathbf{L2})+3H]^+$/ $[^{163}Dy^{163}Dy(\mathbf{L2})+3H]^+$	1179.45 (1179.15)	$[^{164}Dy^{162}Dy(\mathbf{L2})+H]^-$/ $[^{163}Dy^{163}Dy(\mathbf{L2})+H]^-$
[2Dy(**L2**)]	1180.17 (1180.16)	$[^{163}Dy^{162}Dy(\mathbf{L2})+3H]^+$/ $[^{164}Dy^{161}Dy(\mathbf{L2})+3H]^+$	1177.99 (1178.15)	$[^{163}Dy^{162}Dy(\mathbf{L2})+H]^-$/ $[^{164}Dy^{161}Dy(\mathbf{L2})+H]^-$
[2Dy(**L2**)]	1181.78 (1181.16)	$[^{164}Dy^{163}Dy(\mathbf{L2})+3H]^+$	1180.24 (1180.15)	$[^{164}Dy^{163}Dy(\mathbf{L2})+H]^-$
[2Dy(**L2**)]	1183.13 (1183.16)	$[^{164}Dy^{164}Dy(\mathbf{L2})+3H]^+$	1181.16 (1181.15)	$[^{164}Dy^{164}Dy(\mathbf{L2})+H]^-$
[2Dy(**L2**)]	1179.04 (1179.16)	$[^{163}Dy^{161}Dy(\mathbf{L2})+3H]^+$/ $[^{162}Dy^{162}Dy(\mathbf{L2})+3H]^+$	1177.04 (1177.15)	$[^{163}Dy^{161}Dy(\mathbf{L2})+H]^-$/ $[^{162}Dy^{162}Dy(\mathbf{L2})+H]^-$
[2Dy(**L2**)]	1178.00 (1178.16)	$[^{162}Dy^{161}Dy(\mathbf{L2})+3H]^+$	1175.97 (1176.14)	$[^{162}Dy^{161}Dy(\mathbf{L2})+H]^-$
[2Ho(**L2**)]	1185.32 (1185.17)	$[^{165}Ho^{165}Ho(\mathbf{L2})+3H]^+$	1183.27 (1183.15)	$[^{165}Ho^{165}Ho(\mathbf{L2})+H]^-$
[2Er(**L2**)]	1189.22 (1189.17)	$[^{168}Er^{166}Er(\mathbf{L2})+3H]^+$	1187.64 (1187.15)	$[^{168}Er^{166}Er(\mathbf{L2})+H]^-$
[2Er(**L2**)]	1187.63 (1188.17)	$[^{167}Er^{166}Er(\mathbf{L2})+3H]^+$	1185.97 (1186.15)	$[^{167}Er^{166}Er(\mathbf{L2})+H]^-$

[2Er(**L2**)]	1191.34 (1191.17)	$[^{168}Er^{168}Er(\mathbf{L2})+3H]^{+}$/ $[^{170}Er^{166}Er(\mathbf{L2})+3H]^{+}$/	1189.08 (1189.16)	$[^{168}Er^{168}Er(\mathbf{L2})+H]^{-}$/ $[^{170}Er^{166}Er(\mathbf{L2})+H]^{-}$/
[2Er(**L2**)]	1192.24 (1192.17)	$[^{170}Er^{167}Er(\mathbf{L2})+3H]^{+}$	1189.94 (1190.16)	$[^{170}Er^{167}Er(\mathbf{L2})+H]^{-}$
[2Er(**L2**)]	1190.09 (1190.17)	$[^{168}Er^{167}Er(\mathbf{L2})+3H]^{+}$	1187.64 (1188.15)	$[^{168}Er^{167}Er(\mathbf{L2})+H]^{-}$
[2Er(**L2**)]	1187.63 (1187.17)	$[^{166}Er^{166}Er(\mathbf{L2})+3H]^{+}$	1184.88 (1185.15)	$[^{166}Er^{166}Er(\mathbf{L2})+H]^{-}$
[2Er(**L2**)]	1193.36 (1193.17)	$[^{170}Er^{168}Er(\mathbf{L2})+3H]^{+}$	1190.97 (1191.16)	$[^{170}Er^{168}Er(\mathbf{L2})+H]^{-}$
[2Tm(**L2**)]	1193.75 (1193.17)	$[^{169}Tm^{169}Tm(\mathbf{L2})+3H]^{+}$	1191.17 (1191.16)	$[^{169}Tm^{169}Tm(\mathbf{L2})+H]^{-}$
[2Yb(**L2**)]	1200.81 (1201.18)	$[^{174}Yb^{172}Yb(\mathbf{L2})+3H]^{+}$/ $[^{173}Yb^{173}Yb(\mathbf{L2})+3H]^{+}$	1198.88 (1199.17)	$[^{174}Yb^{172}Yb(\mathbf{L2})+H]^{-}$/ $[^{173}Yb^{173}Yb(\mathbf{L2})+H]^{-}$
[2Yb(**L2**)]	1202.88 (1203.18)	$[^{174}Yb^{173}Yb(\mathbf{L2})+3H]^{+}$/ $[^{176}Yb^{171}Yb(\mathbf{L2})+3H]^{+}$	1201.19 (1201.17)	$[^{174}Yb^{173}Yb(\mathbf{L2})+H]^{-}$/ $[^{176}Yb^{171}Yb(\mathbf{L2})+H]^{-}$
[2Yb(**L2**)]	1201.94 (1202.18)	$[^{174}Yb^{174}Yb(\mathbf{L2})+3H]^{+}$/ $[^{176}Yb^{172}Yb(\mathbf{L2})+3H]^{+}$	1200.03 (1200.17)	$[^{174}Yb^{174}Yb(\mathbf{L2})+H]^{-}$/ $[^{176}Yb^{172}Yb(\mathbf{L2})+H]^{-}$
[2Yb(**L2**)]	1200.01 (1200.18)	$[^{174}Yb^{171}Yb(\mathbf{L2})+3H]^{+}$/ $[^{176}Yb^{172}Yb(\mathbf{L2})+3H]^{+}$	1198.08 (1198.17)	$[^{174}Yb^{171}Yb(\mathbf{L2})+H]^{-}$/ $[^{176}Yb^{172}Yb(\mathbf{L2})+H]^{-}$
[2Yb(**L2**)]	1199.16 (1199.17)	$[^{172}Yb^{172}Yb(\mathbf{L2})+3H]^{+}$/ $[^{173}Yb^{171}Yb(\mathbf{L2})+3H]^{+}$	1197.53 (1197.16)	$[^{172}Yb^{172}Yb(\mathbf{L2})+H]^{-}$/ $[^{173}Yb^{171}Yb(\mathbf{L2})+H]^{-}$
[2Yb(**L2**)]	1204.37 (1204.18)	$[^{176}Yb^{173}Yb(\mathbf{L2})+3H]^{+}$	1201.87 (1202.17)	$[^{176}Yb^{173}Yb(\mathbf{L2})+H]^{-}$
[2Yb(**L2**)]	1204.86 (1205.18)	$[^{176}Yb^{174}Yb(\mathbf{L2})+3H]^{+}$	1203.79 (1203.17)	$[^{176}Yb^{174}Yb(\mathbf{L2})+H]^{-}$
[2Lu(**L2**)]	1204.87 (1205.19)	$[^{175}Lu^{175}Lu(\mathbf{L2})+3H]^{+}$	1203.21 (1203.17)	$[^{175}Lu^{175}Lu(\mathbf{L2})+H]^{-}$
[2Y(**L2**)]	1033.20 (1033.11)	$[^{89}Y^{89}Y(\mathbf{L2})+3H]^{+}$	1031.06 (1031.10)	$[^{89}Y^{89}Y(\mathbf{L2})+H]^{-}$

References

(1) Svítok, A.; Blahut, J.; Urbanovský, P.; Hermann, P. Dynamics of Coordinated Phosphonate Group Directly Observed by ^{17}O NMR in Lanthanide(III) Complexes of a Mono(Ethyl Phosphonate) DOTA Analogue. *Chem.–Eur. J.* **2024**, *30*, e202400970. https://doi.org/10.1002/chem.202400970

(2) Svítok, A.; Platas-Iglesias, C.; Kotek, J.; Hermann, P. Phosphorus Chirality Assignment and Solution Dynamics of Lanthanide(III) Complexes of a Monomethylphosphinate Analogue of H_4DOTA: A Multinuclear NMR and DFT Study. *Inorg. Chem. Front.* **2026**, *13*, 637–653. https://doi.org/10.1039/D5QI01885D

(3) Albin, M.; Horrocks, W. D. W.; Liotta, F. J. Characterization of a Potentially Axially Symmetric Europium(III) Complex of a Tetraacetate Tetraaza Macrocyclic Ligand by Luminescence Excitation, Emission and Lifetime Spectroscopy. *Chem. Phys. Lett.* **1982**, *85*, 61–64. https://doi.org/10.1016/0009-2614(82)83461-1

(4) Lebdušková, P.; Hermann, P.; Helm, L.; Tóth, É.; Kotek, J.; Binnemans, K.; Rudovský, J.; Lukeš, I.; Merbach, A. E. Gadolinium(III) Complexes of Mono- and Diethyl Esters of Monophosphonic Acid Analogue of DOTA as Potential MRI Contrast Agents: Solution Structures and Relaxometric Studies. *Dalton Trans.* **2007**, 493–501. https://doi.org/10.1039/b612876a

(5) Aime, S.; Botta, M.; Dickins, R. S.; Maupin, C. L.; Parker, D.; Riehl, J. P.; Williams, J. A. G. Synthesis, NMR, Relaxometry and Circularly Polarised Luminescence Studies of Macrocyclic Monoamidetris(Phosphinate) Complexes Bearing a Remote Chiral Centre. *J. Chem. Soc., Dalton Trans.* **1998**, 881–892. https://doi.org/10.1039/A708667I

(6) Frey, S. T.; Pounds, K. L.; Horrocks, W. D. W.; Chang, C. A.; Carvalho, J. F.; Varadarajan, A.; Schultze, L. M. Characterization of Lanthanide Complexes with a Series of Amide-Based Macrocycles, Potential MRI Contrast Agents, Using Eu^{3+} Luminescence Spectroscopy and Molecular Mechanics. *Inorg. Chem.* **1994**, *33*, 2882–2889. https://doi.org/10.1021/IC00091A032

(7) Aime, S.; Barbero, L.; Botta, M.; Ermondi, G. Determination of Metal-Proton Distances and Electronic Relaxation Times in Lanthanide Complexes by Nuclear Magnetic Resonance Spectroscopy. *J. Chem. Soc., Dalton Trans.* **1992**, 225–228. https://doi.org/10.1039/DT9920000225

(8) Aime, S.; Barge, A.; Botta, M.; Fasano, M.; Danilo Ayala, J.; Bombieri, G. Crystal Structure and Solution Dynamics of the Lutetium(III) Chelate of DOTA. *Inorg. Chim. Acta* **1996**, *246*, 423–429. https://doi.org/10.1016/0020-1693(96)05130-4

(9) Momma, K.; Izumi, F. VESTA 3 for Three-Dimensional Visualization of Crystal, Volumetric and Morphology Data. *J. Appl. Crystallogr.* **2011**, *44*, 1272–1276. https://doi.org/10.1107/S0021889811038970

(10) Evans, D. F. The Determination of the Paramagnetic Susceptibility of Substances in Solution by Nuclear Magnetic Resonance. *J. Chem. Soc.* **1959**, 2003–2005. https://doi.org/10.1039/jr9590002003

(11) Chu, S. C.-K.; Xu, Y.; Balschi, J. A.; Springer, C. S. Bulk Magnetic Susceptibility Shifts in NMR Studies of Compartmentalized Samples: Use of Paramagnetic Reagents. *Magn. Reson. Med.* **1990**, *13*, 239–262. https://doi.org/10.1002/mrm.1910130207

(12) Viswanathan, S.; Kovacs, Z.; Green, K. N.; Ratnakar, S. J.; Sherry, A. D. Alternatives to Gadolinium-Based Metal Chelates for Magnetic Resonance Imaging. *Chem. Rev.* **2010**, *110*, 2960–3018. https://doi.org/10.1021/cr900284a

(13) Procházková, S.; Kubíček, V.; Kotek, J.; Vágner, A.; Notni, J.; Hermann, P. Lanthanide(III) Complexes of Monophosphinate/Monophosphonate DOTA-Analogues: Effects of the Substituents on the Formation Rate and Radiolabelling Yield. *Dalton Trans.* **2018**, *47*, 13006–13015. https://doi.org/10.1039/c8dt02608d
